# The global prevalence of interstitial lung disease in patients with rheumatoid arthritis: a systematic review and meta-analysis

**DOI:** 10.1007/s00296-025-05789-4

**Published:** 2025-01-18

**Authors:** Hari Prasanna, Charles A. Inderjeeth, Johannes C. Nossent, Khalid B. Almutairi

**Affiliations:** 1 School of Medicine, The University of Western Australia, 35 Stirling Highway, Perth, WA 6009 Australia; 2 Pharmacy Department, King Fahd Specialist Hospital, Burydah, Al Qassim, Saudi Arabia; 3 Geronto-Rheumatology, Sir Charles Gairdner and Osborne Park Health Care Group, Perth, WA Australia

**Keywords:** Arthritis, rheumatoid, Connective tissue diseases, Humans, Lung diseases, interstitial, Prevalence, Risk factors

## Abstract

This study aims to review the literature and estimate the global pooled prevalence of interstitial lung disease among patients with rheumatoid arthritis (RA-ILD). The influence of risk factors like geography, socioeconomic status, smoking and DMARD use will be explored. A systematic review was performed according to the PRISMA and JBI guidelines. Studies published between January 1980 and February 2024 were sourced from 7 electronic databases and screened for eligibility. A random-effects meta-analysis model was used to produce pooled prevalences and the potential between-study heterogeneity was identified using sensitivity, subgroup, meta-regression and correlation analyses. 33 studies were included in this meta-analysis containing 14,281 RA patients. The global pooled prevalence of RA-ILD was 21.38% (CI: 0.1542–0.2886), with a high heterogeneity (I^2^) of 98%. The prevalence of usual interstitial pneumonia and non-specific interstitial pneumonia among RA patients were 11.01% and 6.86% respectively. Africa had the highest RA-ILD prevalence with an imprecise estimate of 38.15% (95% Confidence Interval [CI]: 2.29–94.2) and Europe had the lowest prevalence of 10.15% (CI: 2.86–30.23). Other risk factors associated with a higher prevalence of RA-ILD included living in low-income countries, smoking and DMARD use. The biggest limitation of this study is the high heterogeneity of results and underrepresentation of Oceania and low-income countries. This study has clarified the global prevalence of RA-ILD. The risk factors identified in this study can aid clinicians in identifying high-risk populations and highlight the need for screening these populations. Smoking cessation should also be encouraged.

**Supplementary Information:**

The online version contains supplementary material available at 10.1007/s00296-025-05789-4.

## Introduction

Rheumatoid arthritis (RA) is a chronic, autoimmune disease that affects approximately 460 per 100,000 people worldwide [1]. RA primarily targets the joints and is characterised by chronic synovial inflammation leading to joint destruction but it can also have extra-articular manifestations in organs such as the heart, lungs, eyes, skin and kidneys [2, 3]. Interstitial lung disease (ILD) can be a manifestation of RA and is characterised by inflammation and fibrosis of the lungs [4, 5]. It severely impairs lung function and is associated with high morbidity and mortality [6, 7].

A growing body of research suggests a substantial link between RA and ILD, with studies demonstrating that patients with RA are 9 times more likely to develop ILD compared to the general population [8, 9]. Furthermore, being diagnosed with ILD is associated with a threefold increase in mortality compared to RA patients without ILD [10]. As a result, the rising prevalence of ILD and the increased healthcare needs of these patients pose a significant health and financial burden to the world [11, 12].

Despite this impact, the exact prevalence of ILD among patients with RA (RA-ILD) is still unclear with estimates ranging from 2 to 60% of RA patients go on to develop ILD [13, 14]. This heterogeneity results from varying study designs, lifestyle habits, exposures to environmental toxins, differences in genetic predisposition, diagnostic tools used and many more risk factors [15, 16]. There are also different subtypes and radiological patterns of ILD each with varying prevalences, prognosis and management implications [17]. The most prevalent patterns include usual interstitial pneumonia (UIP), non-specific pneumonia (NSIP) and organising pneumonia (OP), with the former having the worst prognosis [17, 18]. Therefore, to understand the underlying aetiology and pathogenesis, it’s important to identify the prevalence of RA-ILD and its patterns and elucidate the risk factors associated with RA-ILD.

There are 2 meta-analyses that have attempted to estimate the global prevalence of RA-ILD so far [14, 19]. These studies had several limitations. The study by Joy et al. focused on multiple connective tissue diseases and therefore had to limit their literature search to just 2 databases and a smaller time period [14]. Additionally, they did not sufficiently analyse sources of heterogeneity in their results [14]. The study by Wang et al. was limited by a poor case definition for ILD, introducing further heterogeneity and reducing the reliability of its prevalence estimate [19].

Therefore, the aim of this systematic review and meta-analysis is to identify the global prevalence of RA-ILD based on a comprehensive search of published population-based studies and to analyse possible risk factors that could explain the heterogeneity of results.

## Materials and methods

### Study design

A systematic literature review was performed according to the Joanna Briggs Institute guidelines for conducting a systematic review of prevalence data and Preferred Reporting Items for Systematic Reviews and Meta-Analyses (PRISMA) 2020 [20, 21]. (Supplementary appendix 1).

### Study selection

Studies published between January 1, 1980, and February 1, 2024, were included in our search.

### Inclusion criteria

Our search strategy consisted of case-control studies, cross-sectional studies, and prospective or retrospective cohort studies. Studies were included if (1) participants had clinically diagnosed RA according to internationally recognised criteria, (2) participants were diagnosed with ILD using a high-resolution computed tomography (HRCT) scan according to the American Thoracic Society/European Respiratory Society’s (ATS/ESR) guidelines on classifying Idiopathic Interstitial Pneumonias, (3) participants were aged 18 years or older, (4) a period or point prevalence was included as one of the outcomes of the study [18, 22].

Inclusion criteria 1 was added because several studies show that the reliability of self-reported RA diagnosis is very low and highly variable ranging around 16–42% [23, 24]. In comparison, the positive predictive value (PPV) of a clinical diagnosis using internationally recognised RA criteria is much higher around 93–95% [25]. Further information on the exact criteria used for diagnosing RA can be found in Supplementary appendix 2.

Previous studies conducted on this topic showed that the use of different diagnostic tools introduced significant heterogeneity [19, 26]. Therefore, we decided to standardise the case definition of ILD with an HRCT scan which has one of the highest sensitivity and PPV for diagnosing RA-ILD [27]. We screened studies to ensure they are consistent with the radiological features as defined by the ATS/ESR guidelines when classifying ILD and its patterns (Supplementary Appendix 3) [18, 22]. Studies using secondary diagnostic tools like a chest x-ray, pulmonary function tests and surgical lung biopsy in addition to an HRCT scan are still included.

### Exclusion criteria

Studies were excluded if (1) participants were diagnosed with ILD before a diagnosis of RA, (2) participants were diagnosed with other causes of ILD such as sarcoidosis, systemic sclerosis, myositis, systemic lupus erythematosus, sjögren’s syndrome, hypersensitivity pneumonitis, pneumoconiosis and radiation pneumonitis (3) participants were diagnosed with ILD without the use of a HRCT scan, (4) comprised non-primary literature like editorials, narratives, systematic reviews, case studies/series, (5) published in a language other than English [27].

### Search strategy

A literature search was conducted according to the PRISMA 2020 recommendations to locate studies in relevant databases, including MEDLINE (Ovid), Embase, Google Scholar, Scopus, Web of Science, Proquest Central and Cinahl. The first author (HP) constructed the search strategy with the help of a UWA health sciences librarian (JW). This strategy was peer-reviewed by the supervising author (KA). The search strategy was first developed in Medline using MeSH subject headings and free-text terms around the four search components ‘Prevalence’, ‘Rheumatoid Arthritis’, ‘Interstitial Lung Disease’ and ‘X-ray Computed Tomography’ as shown in Table 1. These terms were then combined using ‘AND’ and ‘OR’ operators to conduct the search. This strategy was adapted to other databases’ syntax and subject headings (Supplementary appendix 4).

**Table 1:** MEDLINE search strategy

1. exp Arthritis, Rheumatoid/
2. Rheumatoid Arthritis.tw.
3. RA.tw.
4. 1 or 2 or 3
5. exp Lung Diseases, Interstitial/
6. diffuse parenchymal lung.mp.
7. pulmonary fibrosis.mp.
8. Interstitial lung disease*.mp.
9. Rheumatoid lung.mp.
10. RA-ILD.mp.
11. ILD.mp.
12. UIP.mp.
13. NSIP.mp.
14. Alveolitis.mp.
15. Organi$ing pneumon*.mp.
16. 5 or 6 or 7 or 8 or 9 or 10 or 11 or 12 or 13 or 14 or 15
17. exp Prevalence/
18. Prevalence*.mp.
19. Population-based study.mp.
20. Epidemiology.mp.
21. Trends.mp.
22. Rate.mp.
23. 17 or 18 or 19 or 20 or 21 or 22
24. exp tomography, x-ray computed/ or exp computed tomography angiography/ or exp tomography, spiral computed/
25. HRCT.mp.
26. CT scan.mp.
27. Computed Tomography.mp.
28. 24 or 25 or 26 or 27
29. 4 and 16 and 23 and 28
30. Limit 29 to yr="1980–2024” and human and English language

### Data management

Literature search results were first uploaded to Endnote version 21 in order to discard duplicate and abstract-only publications. The remaining studies were uploaded to the ‘Rayyan AI’ software to begin screening. HP and KA independently reviewed the titles and abstracts identified through the search against the inclusion and exclusion criteria. Full reports were obtained for studies that aligned with the inclusion criteria and in cases of uncertainty. The authors then assessed the full-text reports to determine whether they conformed with the inclusion and exclusion criteria. Any disagreements were resolved by discussion with the consultation of a third senior author (CI). Cohen’s kappa statistic will be used to assess inter-rate agreement.

### Data extraction and quality assessment

The data was extracted by HP and displayed in a standardised form using a spreadsheet software (Microsoft Excel). The following data were extracted from all studies: the first author’s name, year of publication, study design, country of study, RA classification criteria used, sample size, period of recruitment and prevalence of RA-ILD (the primary outcome of this study).

If available, the participant’s baseline characteristics were also collected: mean age, mean disease duration, ratio of males to females, prevalence of smokers, disease-modifying anti-rheumatic drugs (DMARD) use prevalence and the prevalence of different ILD patterns. This data was only collected if it represented the entire sample.

The quality of included studies was appraised by HP using the risk of bias tool by Hoy et al. [28] (Supplementary appendix 5). This tool classifies prevalence studies into low, moderate or high risk based on its 10-question criteria. To minimise errors, the extracted data and quality assessment results were double-checked for each study and the results were reviewed by KA. Any disagreements were resolved with discussion.

### Data synthesis

The prevalence of RA-ILD was calculated by dividing the number of RA-ILD cases by the total number of participants with RA. All statistical analyses were performed using the R programming language with the ‘meta’ and ‘dmetar’ packages [29].

Pooled estimates of the prevalence of RA-ILD and the prevalence of RA-ILD patterns were calculated using a random effects meta-analysis model to account for the anticipated heterogeneity between studies [30]. The logit transformation was used for variance stabilisation of proportions before pooling the data with the random-effects model. The meta-prop command generated forest plots of pooled prevalence with 95% confidence intervals. The Cochran’s Q test and I^2^ test were used to assess the magnitude of heterogeneity where thresholds of ≥ 25%, ≥ 50% and ≥ 75% indicate low, moderate and high heterogeneity respectively [31].

To assess the robustness of our results, sensitivity analysis was performed by leaving one study out at a time, excluding studies with moderate risk of bias and excluding studies with potential outliers. Extreme outliers were identified using a Baujat plot [32].

Subgroup analyses were performed if two or more studies can be found for each of the following subgroups:-.

1. **Geography** – Classified by the country and continent where the study participants were recruited.
2. **Time periods** – Grouped as participants recruited before or after 1st June 2014.
3. **RA classification criteria** – Stratified by the 2010 American College of Rheumatology/European League Against Rheumatism (ACR/EULAR) criteria and/or the 1987 revised American Rheumatism Association (ARA) criteria.
4. **Socioeconomic status** – Classified according to the World Bank’s income classification.
5. **Sampling methodology** – Participants were either sampled or patient records were reviewed retrospectively.
6. **Risk of Bias** – Classified as low, moderate or high risk of bias.

Univariate and multivariate meta-regression was conducted with a mixed-effects model to assess the impact of these subgroups on pooled prevalence estimates. The Pearson and Spearman-rank’s correlations were also used to investigate any correlations between age, gender, disease duration, smoking status or DMARD use and the prevalence of RA-ILD.

Lastly, publication bias was assessed qualitatively using funnel plots and quantitatively with Begg-Mazumdar and Egger’s linear regression tests. A p-value of less than 0.05 was used as the threshold for bias [33, 34].

## Results

### Study selection

**Fig. 1:**
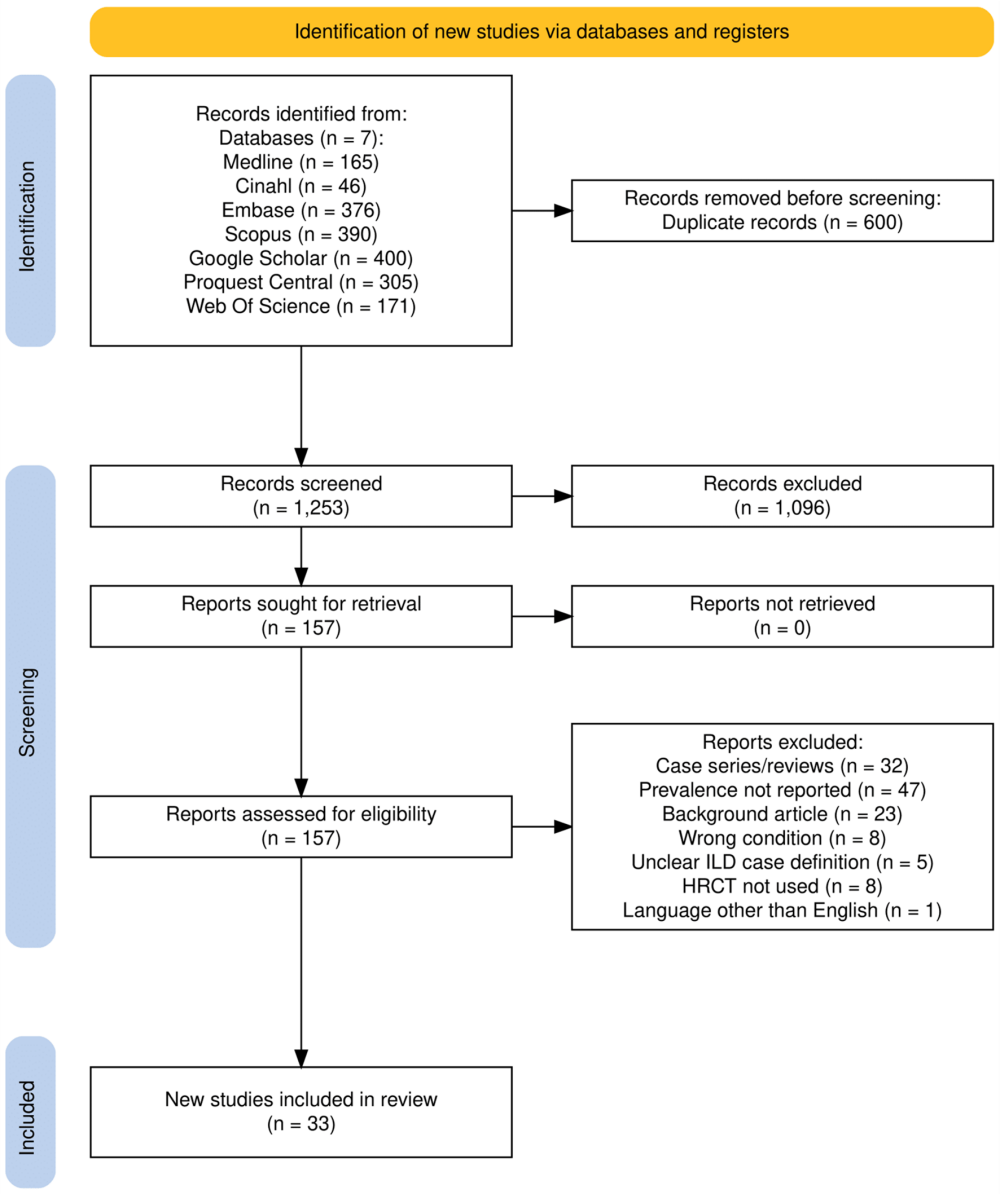
PRISMA flow diagram of the search and screening process

Our systematic search provided a total of 1853 studies from Medline (*n* = 165), Cinahl (*n* = 46), Embase (*n* = 376), Scopus (*n* = 390), Google Scholar (*n* = 400), Proquest Central (*n* = 305) and Web of Science (*n* = 171) as shown in Fig. 1 [35]. Firstly, 600 duplicate publications were found and discarded. After an abstract and title screen, 157 studies were selected for further evaluation. Following full study screening for the eligibility criteria, 33 studies were selected for inclusion. 2 of these studies had multiple cohorts, and each cohort was recognised separately during analysis. The total number of cohorts analysed was 35.

Cohen’s kappa statistic for inter-rater agreement was 0.15 with an agreement percentage of 87.95% [36].

### Study characteristics

The characteristics of the included 33 population-based studies are displayed in Table 2. Here is a summary of the characteristics:-.

• Study participants: Our study had a total of 14,281 RA patients in 35 cohorts consisting of 2371 patients with RA-ILD and 11,910 RA patients without ILD. The sample sizes of the studies varied from 30 to 2729 people (Mean = 408, SD = 574).

• Geographical distribution: The 33 studies were conducted across 16 different countries. Approximately half of the cohorts were in Asia (*n* = 16, 45.7%). This was followed by Europe (*n* = 6, 17.1%), North America (*n* = 5, 14.3%), Africa (*n* = 4, 11.4%) and South America (*n* = 3, 8.57%) all being represented by a similar proportion of studies. Only 1 study was performed in Oceania (2.86%).

• Prevalence methods and sampling methodology: The point prevalence was reported in 9 cohorts (25.7%) and period prevalence was reported in 26 cohorts (74.3%). The point prevalence was more common in prospective studies where patients were sampled from a representative RA population (*n* = 19, 54.3%), whereas the period prevalence is more commonly used in retrospective studies that analyse large databases of patient records (*n* = 16, 45.7%).

• Recruitment time-period: Participants were recruited over different time periods spanning from 1986 to 2021. 9 cohorts (25.7%) recruited participants within the last 10 years (after June 2014) and 13 cohorts (37.1%) before, with 6 cohorts (17.1%) recruiting participants across both time periods. Only 2 studies recruited participants between 1986 and 1998 but none recruited participants between 1998 and October 2004.

• Risk of bias assessment: The risk of bias was low in 28 studies (84.8%) and moderate in 5 studies (15.2%). There were no studies with a high risk of bias. Some reasons for a moderate risk of bias included: a long prevalence period due to participants being recruited over many years, selection bias due to a lack of randomisation and recruitment from a single hospital/site, and the collection of data by proxy in retrospective studies. The exact scoring of each study can be found in Supplementary Appendix 7.

• Classification criteria: The most commonly used diagnostic criteria for RA was the revised 1987 ARA criteria (*n* = 21, 60%) followed by the 2010 ACR/EULAR criteria (*n* = 20, 57.1%). 6 cohorts used a combination of the ACR/EULAR 2010 and the 1987 ARA criteria (17.1%).

• Socioeconomic status: The World Bank Income’s classification was used to classify the socioeconomic status of countries where these studies took place [37]. 16 cohorts (45.7%) were recruited from countries classified as ‘High income’, while 13 and 6 cohorts were recruited from countries classified as ‘upper-middle income’ and ‘lower-middle income’ countries respectively. No studies were conducted in ‘low income’ countries.

**Table 2:** Study characteristics of the 33 included studies

Author	Year of study	Study Design		Country of study	RA Classification criteria	Sampling Methodology	Sample size	Prevalence method		Prevalence of ILD	Risk of bias assessment
			Socioeconomic status						Time period of recruitment		
Gabbay et al. [43]	1997	Prospective cohort	High Income	Australia	1987 ARA	Sampled	36	PointP	N/A	0.3333	Low
Koduri et al. [52]	2010	Retrospective cohort	High Income	England	1987 ARA	Patient records	1460	PeriodP	1986–1998	0.0356	Low
Mori et al. [53]	2011	Prospective cohort	High Income	Japan	1987 ARA	Sampled	189	PeriodP	Jan 2009 - May 2009	0.1005	Low
Zou et al. [54]	2012	Prospective cohort	Upper Middle Income	China	1987 ARA	Sampled	110	PeriodP	Dec 2008 - Nov 2009	0.4273	Low
Giles et al. [55]	2014	Prospective cohort	High Income	USA	1987 ARA	Sampled	176	PeriodP	Oct 2004 - May 2006	0.3295	Low
Wang et al. [56]	2015	Retrospective cohort	Upper Middle Income	China	1987 ARA	Patient records	544	PeriodP	July 2006 - June 2011	0.1526	Low
Chen et al. USA [57]	2015	Prospective cohort	High Income	USA	1987 ARA	Sampled	86	PeriodP	October 2010–June 2013	0.5698	Low
Chen et al. China [57]	2015	Prospective cohort	Upper Middle Income	China	1987 ARA	Sampled	133	PeriodP	July 2012 - March 2013	0.3083	Low
Okada et al. [58]	2016	Retrospective cohort	High Income	Japan	1987 ARA	Patient records	499	PointP	Dec 2010	0.1680	Moderate
Song et al. [59]	2016	Prospective cohort	High Income	South Korea	1987 ARA	Sampled	116	PeriodP	Sep 2013 - May 2014	0.0690	Low
Kim et al. [45]	2017	Retrospective cohort	High Income	South Korea	1987 ARA	Patient records	244	PeriodP	July 2009 - Dec 2012	0.098	Low
Fadda et al. [40]	2018	Cross-sectional study	Lower Middle Income	Egypt	2010 ACR/EULAR	Sampled	88	PointP	N/A	0.716	Low
Salaffi et al. [60]	2019	Retrospective cohort	High Income	Italy	2010 ACR/EULAR	Patient records	151	PeriodP	Jan 2014 - Jun 2018	0.1921	Moderate
Li et al. [61]	2019	Retrospective cohort	Upper Middle Income	China	1987 ARA or 2010 ACR/EULAR	Patient records	1096	PeriodP	Oct 2008 - Oct 2017	0.3841	Moderate
Sherin et al. [62]	2019	Cross-sectional study	Lower Middle Income	Egypt	2010 ACR/EULAR	Sampled	100	PointP	N/A	0.36	Low
Manfredi et al. [63]	2019	Prospective cohort	High Income	Italy	1987 ARA or 2010 ACR/EULAR	Sampled	137	PointP	N/A	0.4307	Low
Fu et al. [64]	2019	Retrospective cohort	Upper Middle Income	China	1987 ARA or 2010 ACR/EULAR	Patient records	791	PeriodP	May 2008 - Jan 2014	0.3881	Low
England et al. [65]	2019	Retrospective cohort	High Income	USA	1987 ACR	Patient records	1823	PeriodP	N/A	0.0494	Low
Gautam et al. [66]	2020	Prospective cohort	Lower Middle Income	India	2010 ACR/EULAR	Sampled	54	PeriodP	Jun 2014 - Jun 2015	0.3704	Low
Castellanos-moreira et al. [67]	2020	Cross-sectional study	High Income	Spain	2010 ACR/EULAR	Sampled	148	PeriodP	July 2017 - July 2018	0.0405	Low
Li L et al. [68]	2020	Retrospective cohort	Upper Middle Income	China	1987 ARA or 2010 ACR/EULAR	Patient records	923	PeriodP	May 2008 - Oct 2017	0.3012	Low
Wickrematilake et al. [69]	2021	Prospective cohort	Lower Middle Income	Srilanka	1987 ARA or 2010 ACR/EULAR	Sampled	384	PointP	N/A	0.1458	Low
Paulin et al. [70]	2021	Prospective cohort	Upper Middle Income	Argentina	2010 ACR/EULAR	Sampled	79	PeriodP	Dec 2017 - Feb 2020	0.0759	Low
Liang et al. Discovery cohort [71]	2021	Prospective	Upper Middle Income	China	2010 ACR/EULAR	Sampled	70	PointP	Jan 2020 - Sep 2020	0.2	Low
Liang et al. Identification cohort [71]	2021	Retrospective	Upper Middle Income	China	2010 ACR/EULAR	Sampled	98	PointP	Jan 2020 - Oct 2020	0.3469	Low
Samhouri et al. [72]	2022	Retrospective cohort	High Income	USA	1987 ACR	Patient records	623	PeriodP	1999–2014	0.0819	Low
Gutierrez et al. [73]	2022	Prospective cohort	Upper Middle Income	Mexico and Argentina	2010 ACR/EULAR	Sampled	74	PointP	N/A	0.3649	Low
Bonilla Hernan et al. [74]	2022	Prospective cohort	High Income	Spain	1987 ARA or 2010 ACR/EULAR	Sampled	2729	PeriodP	2007–2018	0.0330	Low
Denis A et al. [75]	2022	Retrospective cohort	High Income	Belgium	2010 ACR/EULAR	Patient records	523	PeriodP	Jan 2010 - Jan 2020	0.1702	Low
Severo et al. [76]	2022	Retrospective cross-sectional	Upper Middle Income	Brazil	2010 ACR/EULAR	Patient records	134	PeriodP	Mar 2019 - Dec 2019	0.3657	Low
Abdelwahab et al. [42]	2022	Cross-sectional study	Lower Middle Income	Egypt	2010 ACR/EULAR	Sampled	30	PeriodP	Nov 2018 - Nov 2019	0.733	Low
Sanaa et al. [41]	2023	Prospective cohort	Lower Middle Income	Tanzania	2010 ACR/EULAR	Sampled	132	PeriodP	N/A	0.0303	Low
Razmjou et al. [77]	2023	Retrospective cohort	High Income	USA	1987 ARA	Patient records	108	PeriodP	May 2021	0.1481	Low
Ren et al. [47]	2023	Retrospective cohort	Upper Middle Income	China	2010 ACR/EULAR	Patient records	154	PeriodP	Jan 2012 - Aug 2021	0.435	Moderate
Yu et al. [78]	2023	Retrospective cohort	Upper Middle Income	China	1987 ARA	Patient records	239	PeriodP	2019–2021	0.251	Moderate

### Prevalence of RA-ILD

**Fig. 2:**
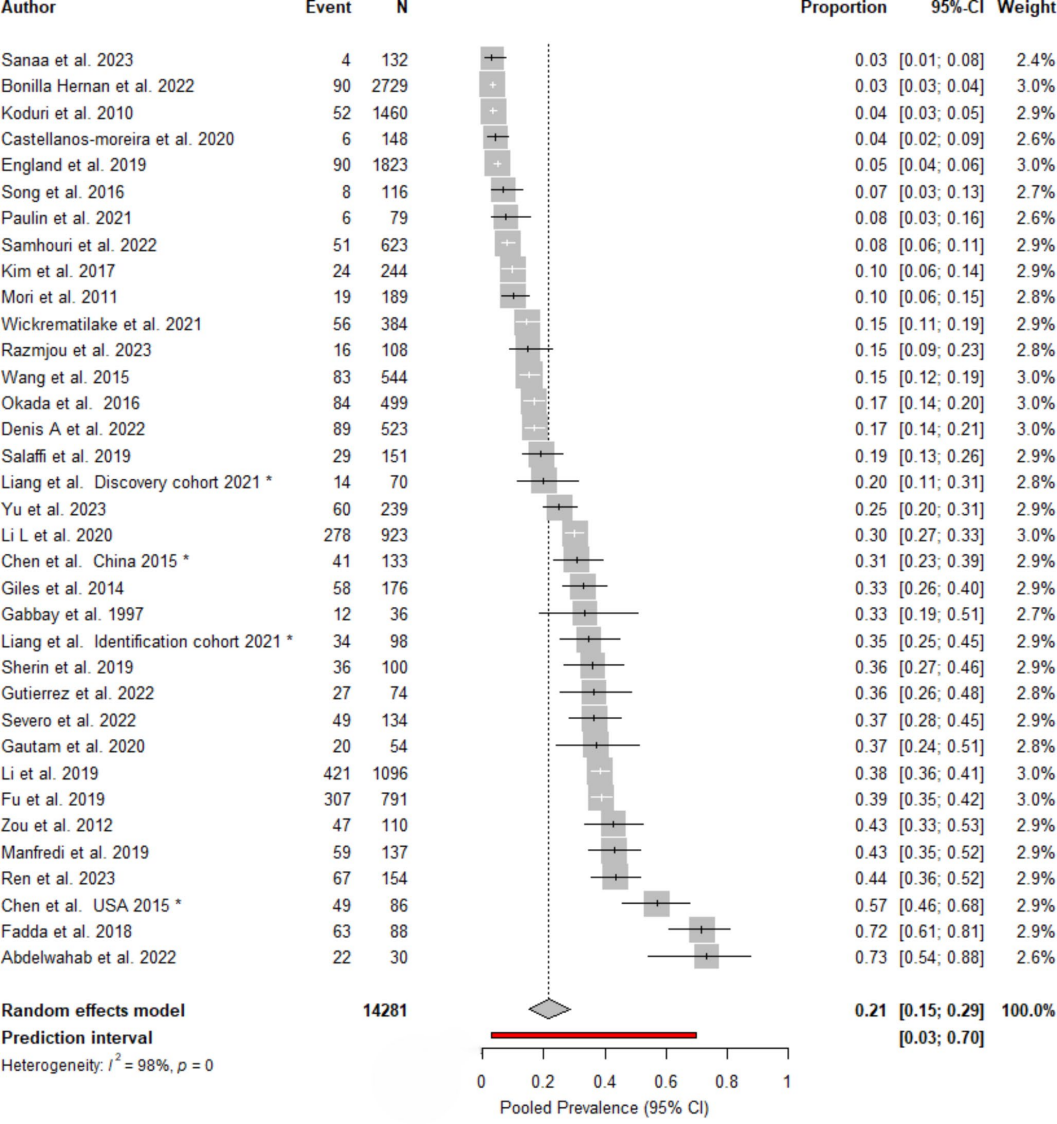
Forest plot of the 35 cohorts included in this systematic review

The prevalence of RA-ILD in the 35 cohorts ranged from 3.03% to 73.3%. The global pooled prevalence of RA-ILD by random effects model was 21.38% (95% CI: 15.42–28.86) as seen in the forest plot in Fig. 2. The between-study heterogeneity was high (I^2^ = 98%).

The pooled prevalence of ILD patterns was also calculated using data from 10 studies that reported this information. The pooled prevalence of RA-UIP was 11.01% (95% CI: 4.85–23.10, I^2^ = 97.2%), the prevalence of RA-NSIP was 6.86% (95% CI: 2.52–17.36, I^2^ = 95.5%) and the prevalence of RA-OP was 1.64% (95% CI: 0.54–4.84, I^2^ = 93.7%).

### Sensitivity analysis

According to our 3 sensitivity analyses, the pooled prevalence findings were robust. Based on a ‘leave-one-out’ method, there were no significant differences in the global pooled prevalence or heterogeneity. As shown in Supplementary appendix 13, the RA-ILD pooled prevalence varied between 20% and 23%, while the heterogeneity varied from 97% to 98%. Similarly, when excluding studies with a moderate risk of bias, there was no statistically significant difference in the pooled prevalence of RA-ILD (prevalence = 20.41%, *p* = 0.867). Lastly, 5 influential outliers were identified and confirmed using the Baujat plot (Supplementary appendix 13). When these outliers were removed, the prevalence of RA-ILD was 24.04% and the heterogeneity reduced to 94.8% but this difference was not statistically significant either (*p* = 0.598).

### Subgroup analysis

The subgroups that we analysed in relation to RA-ILD consisted of geographical locations, RA classification criteria used, risk of bias, sampling methodology, socioeconomic status and time period of recruitment as seen in Supplementary appendix 9. There was still high heterogeneity (I^2^ > 75%) in all subgroups. Similarly, there were no statistically significant differences in RA pooled prevalences between subgroups except for socioeconomic status (*p* = 0.0042).

### Pooled prevalence by continents and countries

The highest RA-ILD pooled prevalence was in Africa at 38.15% (CI = 2.29–94.2), followed by South America at 24.64% (CI = 2.18–82.78) and Asia at 24.01% (CI = 17.56–31.92). The lowest pooled estimates were found in Europe with a prevalence of 10.15% (CI = 2.86–30.23) and in North America with a prevalence of 18% (CI = 4.15–52.67). Oceania had only 1 study from Australia with a prevalence of 33.3%. These estimates are very imprecise as there is high heterogeneity and variation in prevalences between countries within the same continent. For instance, on the country level, the study with the lowest prevalence of RA-ILD was in Tanzania with an estimate of 3.03%, and the study with the highest prevalence was in Egypt with an estimate of 73.3%, which are both countries in Africa.

### Pooled prevalence by classification criteria

The highest RA-ILD pooled prevalence was found in studies using the 2010 ACR/EULAR criteria with an estimate of 25.60% (CI = 16.24–37.92). The 1987 revised ARA criteria had a lower pooled prevalence estimate of 18.43% (CI = 12.20–26.86).

### Pooled prevalence by risk of bias

The pooled prevalence estimate of studies with low risk of bias was 20.41% (CI = 13.91–28,92) which is comparable to the overall pooled prevalence of RA-ILD but studies with moderate risk of bias seem to overestimate the prevalence of RA-ILD (27.56% CI = [15.51–44.09]).

### Pooled prevalence by sampling methodology

The pooled prevalence was higher in prospective studies where participants were sampled with an estimate of 23.54% (CI = 13.77–37.24%) compared to studies using databases with patient health records which had an estimate of 18.95% (CI = 12.5–27.67). Retrospective studies tended to have larger sample sizes resulting in a more precise pooled prevalence estimate.

### Pooled prevalence by socioeconomic status

The highest RA-ILD pooled prevalences were found in lower-middle and upper-middle income countries with estimates of 32.99% (CI = 7.92–73.81) and 30.41% (CI = 23.60–38.20) respectively. However, high-income countries had a significantly lower estimate of 13.33% (CI = 7.84–21.74%).

### Pooled prevalence by recruitment time-periods

The studies were separated into the following 3 subgroups. Studies before June 2014, after June 2014 and studies that recruited participants across both time periods. This was done to minimise the number of studies that recruited participants across both time periods. The number of studies published on this topic have also increased dramatically over the last decade compared to before 2014 which is reflected in the number of studies included past 2014. Therefore choosing 2014 as a checkpoint allowed enough studies before and after to do a fair comparison.

The lowest prevalence of RA-ILD was estimated in studies that took place before June 2014, with a prevalence of 19.19% (CI = 11.06–31.21) and the highest prevalence was after June 2014 with a prevalence of 24.04% (CI = 11.41–43.74). Studies that overlap had a pooled prevalence of 20.93% (CI = 7.46–46.48). However, these subgroups had the largest p-value of 0.945 when testing for differences between subgroups.

### Meta-regression

Both univariate and multivariate regression analyses were performed to identify potential sources of between-study heterogeneity (Supplementary appendix 10). According to univariate analysis, the biggest sources of inter-study heterogeneity include socioeconomic status and geographical location accounting for 39.36% and 30.97% respectively. Using the multivariable meta-regression model the subgroups together can explain 46.38% of the inter-study heterogeneity.

### Correlation analysis

**Fig. 3:**
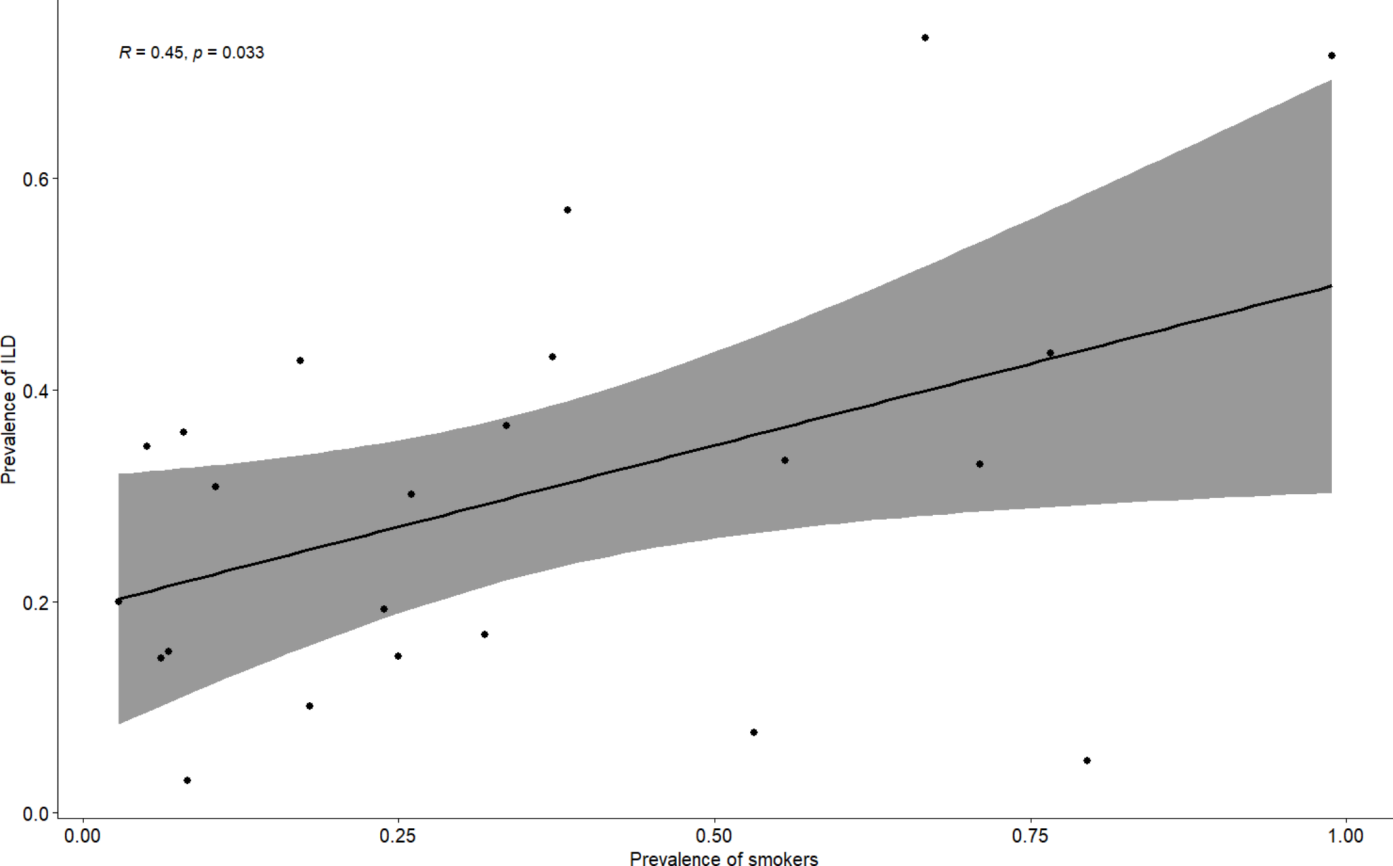
Pearson correlation analysis of the prevalence of smoking and RA-ILD

Pearson correlation was used to assess for associations between risk factors like smoking, DMARD use (Methotrexate or leflunomide), age, gender and RA disease duration as seen in Fig. 3 and Supplementary appendix 11.

Samples with higher smoking rates were correlated with a higher prevalence of RA-ILD according to Pearson correlation (*R* = 0.45, p-value = 0.033).

None of the other risk factors showed a statistically significant correlation with the prevalence of RA-ILD except for a weak positive trend seen with the prevalence of methotrexate use (*R* = 0.31, p-value = 0.27) and leflunomide use (*R* = 0.33, p-value = 0.43) according to Pearson correlation.

### Publication bias

**Fig. 4:**
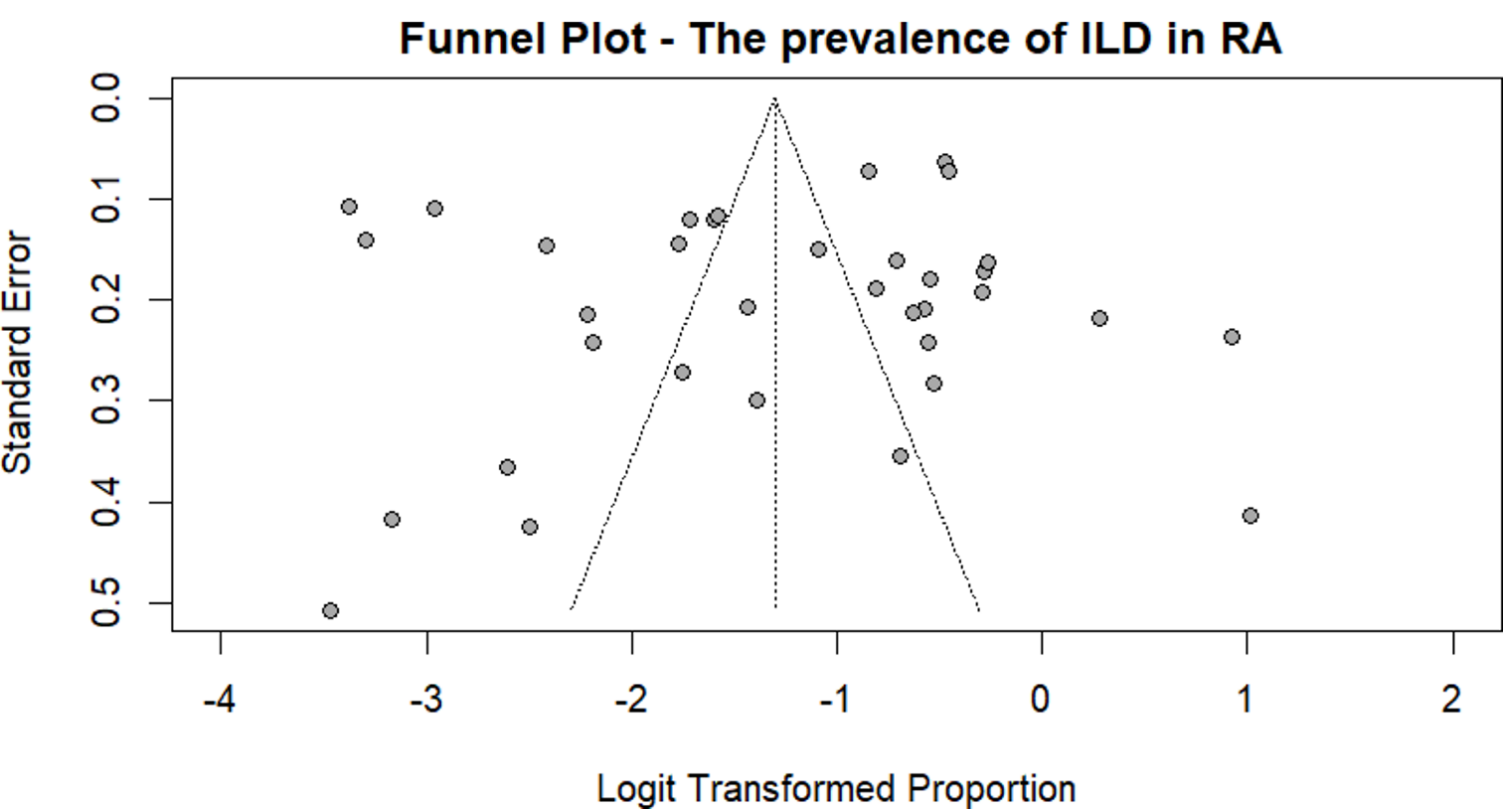
Funnel plot of the prevalence of RA-ILD

The publication bias was not statistically significant according to the Begg-Mazumdar (*p* = 0.7014) and Egger’s (*p* = 0.5209) linear regression test, which primarily tests for symmetry [33, 34]. Although the studies are distributed symmetrically in the funnel plot, many studies lie outside the 95% CI lines as seen in Fig. 4. This discrepancy in the quantitative and qualitative analysis of publication bias is due to the high heterogeneity of results and is a limitation of these regression tests [38].

## Discussion

This comprehensive systematic review confirms that ILD is a common extra-articular manifestation of RA and is significantly more prevalent in RA patients compared to the general population. As seen with previous studies conducted on this topic, there is high variability in the RA-ILD prevalences across different subsets of the global population [14, 19, 26].

Across 33 included studies, the global pooled prevalence of RA-ILD was 21.38% from 1980 to 2024. This is almost 2 times higher than the estimated RA-ILD prevalence of 11% by Joy et al. and aligns better with a recently published systematic review by Wang et al. with an estimate of 18.7% [14, 19].

The differences in the case definitions for RA-ILD may account for the differences in results between our study and Wang et al. [19]. Our review only included studies where participants were diagnosed with ILD with an HRCT scan. However, the review by Wang et al. also accepted other diagnostic tools like chest x-rays, standard CT scans, pulmonary function tests, lung biopsies and some studies even relying on international classification of diseases (ICD) codes without confirmation with a diagnostic tool [19]. This seems to have underestimated the prevalence of RA-ILD and reduced the reliability of the diagnosis. When limiting the analysis to studies using only HRCT scans, they found that their pooled prevalence estimate increased to 22.7% [19].

The meta-analysis by Joy et al. was limited by a wider focus on different types of connective tissue disorders instead of focusing on rheumatoid arthritis [14]. Their search strategy was constrained to a smaller time period and just 2 databases resulting in fewer studies being included. As a result, studies with higher prevalences were missed in their review despite meeting their eligibility criteria, underestimating the prevalence of RA-ILD.

In addition to the prevalence of RA-ILD, we identified the pooled prevalence of the 3 most common RA-ILD patterns (UIP, NSIP and OP) which has not been done in prior reviews. UIP was the most prevalent radiological pattern in patients with RA-ILD at 11.01% followed by NSIP at 6.86% and OP at 1.64%. This is important to distinguish because UIP has a significantly worse prognosis compared to NSIP and OP [17]. Therefore, these results highlight the importance of screening and stratifying patients with RA-UIP earlier to provide treatment earlier and improve their health outcomes [39].

Information regarding risk factors of RA-ILD was sparse despite the big impact it has on the prevalence estimates. Geography, for instance, plays a significant role in the variability of the prevalence of RA-ILD. The continents can be classified into 3 broad categories based on prevalence. Europe and North America had the lowest pooled prevalences (10.15%, 18%), Asia and South America had a moderate pooled prevalence (24.01%, 24.64%), and Africa and Oceania had the highest pooled prevalences (38.15%, 33.3%). However, these estimates were very imprecise with large confidence intervals due to the significant variations between countries of the same continent. For instance, a study by Sanaa et al. in Tanzania had a significantly lower prevalence (3.03%) compared to studies by Fadaa et al. (71.6%) and Abdelwahab et al. (73.3%) in Egypt [40–42]. This is despite both countries being in Africa, having similar sample sizes and a low risk of bias scoring. These differences in RA-ILD prevalence based on geography may suggest that genetics or the environment may be important factors in disease development. The high prevalence of RA-ILD in Oceania may be an outlier as it is based on a single outdated Australian study by Gabbay et al. conducted in 1997 [43].

Variations between continents may also be better explained by differences in the socioeconomic status of their respective countries. Countries in Africa were more likely to be classified as ‘lower-middle income’ countries and countries in Europe and North America were classified as ‘high-income’ countries according to the World Bank’s income classifications [37]. When pooling countries according to this classification we found that the prevalence of RA-ILD was lowest in high-income countries like the USA, England and Belgium and highest in ‘upper-middle income’ and ‘lower-middle income’ countries like Egypt, India and China.

The type of RA classification criteria used had a significant impact on the pooled prevalence of RA-ILD. The ACR/EULAR criteria produced a significantly higher prevalence (25.60%) than the 1987 ARA criteria (18.43%). This could be related to the increased sensitivity and decreased specificity of the 2010 ACR/EULAR criteria in comparison to the revised 1987 ARA criteria [44]. It may also be correlated with the fact that newer studies tend to use the 2010 ACR/EULAR criteria and studies that recruited participants after June 2014 had a higher prevalence (24.04%) than studies before (19.19%).

There was also a notable difference in pooled prevalence based on sampling methodology. Retrospective studies using patient records were found to estimate a lower RA-ILD prevalence (18.95%) than prospective studies (23.54%). A potential cause for these differences could be due to misclassification of RA-ILD patients when data is collected by proxy in retrospective studies. For example, studies like Kim et al. search for relevant keywords in radiology reports rather than analysing the HRCT scans directly [45]. This could result in a greater number of errors, due to missing/implied information.

Smoking status seems to be a significant risk factor associated with the prevalence of RA-ILD [46]. Based on correlation analyses there was a statistically significant correlation between the prevalence of smokers in a study and the prevalence of RA-ILD. This is in line with other studies like Kronzer et al. which found that smoking 30-pack years had a strong association with RA-ILD [46, 47]. The use of DMARDS like leflunomide and methotrexate had a weak correlation with the prevalence of RA-ILD but these results were not statistically significant. The literature on this topic is divisive with studies by Juge et al. showing that Methotrexate use can instead be a protective factor and delay the development of RA-ILD [48]. It is important to understand that these results only suggest correlations and do not imply causation. Therefore, more rigorous research like controlled trials or large cohort studies exploring the effect of DMARDs on RA-ILD is required, as this can have big implications for the pharmacological management of RA patients.

Other risk factors like age, gender, and RA disease duration did not show any correlations with the prevalence of RA-ILD. Odds/hazard ratios may be more useful to identify these associations but there wasn’t sufficient data available in the included studies to carry out these analyses.

### Implications

The prevalence of RA-ILD is high and given the poor prognosis and limited treatment options available for this condition, further research is critically required in this field [10, 49]. In the meantime, screening programmes may be beneficial to identify patients with RA-ILD and refer them to specialist services earlier to improve health outcomes [39]. This is particularly important in subsets of the global population like people from low socioeconomic areas or geographical locations associated with a higher prevalence of developing RA-ILD. Lifestyle modifications like smoking cessation should also be encouraged by physicians given its association with a lower prevalence of RA-ILD in addition to numerous other health benefits [50].

### Strengths

There are several strengths to this systematic review and meta-analysis lies. Firstly, several steps were taken during the eligibility criteria to reduce the heterogeneity of our results. The case definition of ILD was standardised across all the included studies, possible confounding causes of ILD were eliminated and internationally recognised criteria were required to include participants with RA.

This study sourced studies from more databases than any previous systematic review done on this topic. The prevalences of different radiological patterns of ILD were also identified for the first time in a meta-analysis of this topic. Causes for high heterogeneity were identified using extensive, subgroup analysis, correlation analysis, meta-regression analysis and publication bias analysis. The findings of the global pooled prevalences were robust and not influenced by potential outliers, single studies or studies with a moderate risk of bias.

### Limitations

It is important to acknowledge the limitations of this study. The biggest limitation of this study lies in the high magnitude of heterogeneity (I^2^ > 90) between studies. As a result, the prevalence estimates had large confidence intervals and imprecise estimates. Despite standardising the diagnosis of ILD with HRCT scans, there were still variations in case definitions of ILD, like the use of different methods to interpret HRCT scans and the use of secondary diagnostic tools. This may have reintroduced heterogeneity in the results.

Another limitation is that different regions of the world are not equally represented in our systematic review. Around half of the studies in this review were from Asia while only 1 study was included from Oceania. Additionally, there were no studies from a low-income country during analysis on the effects of socioeconomic status. Combined with the high heterogeneity, this limits the generalisability of our prevalence estimate to a global scale.

There may be an element of selection bias in the studies that scored as a moderate risk of bias during quality assessment. For example, in the study by Salaffi et al., participants were screened for ILD with an HRCT scan after presenting with symptoms of ILD, which may overestimate the prevalence of RA-ILD [51].

In this review, we chose not to include patients diagnosed with interstitial lung disease before the onset of rheumatoid arthritis or participants with mixed connective tissue disorders to reduce possible confounders but as a result, RA-ILD patients in this subgroup are not represented. Studies published in a language other than English and unpublished/grey literature were also not included in this review.

Lastly, it is important to understand that the risk factors identified during subgroup and correlation analyses do not imply causation.

### Future research directions

In light of these results, we recommend that:-.

1. Future systematic reviews should narrow their focus/target population to reduce heterogeneity or identify new risk factors that better explain the source of heterogeneity.
2. Given the high prevalence of RA-ILD, future research could investigate whether screening populations with the risk factors identified in this study can improve patient outcomes.
3. More population health studies investigating the prevalence of RA-ILD should be conducted in underrepresented regions like Oceania and further reviews should include underrepresented populations like patients with mixed connective tissue disorders.

## Conclusion

The global prevalence of RA-ILD was 21.38% from 1980 to 2024. UIP is the most common radiological pattern in patients with RA-ILD, making up around 11% of RA patients. Risk factors that increased the prevalence of RA-ILD include living in geographical locations like Africa and South America, living in a country with a low socioeconomic status and smoking. Using the ACR/EULAR 2010 criteria seems to be more sensitive to RA patients with ILD compared to older classification criteria and studies with a moderate risk of bias overestimate the prevalence of RA-ILD. These findings improve our understanding of RA-ILD and highlight the crucial need for screening programs and new treatment options given the poor prognosis of this condition [39].

## Electronic supplementary material

Below is the link to the electronic supplementary material.

Supplementary Material 1

Supplementary Material 10

Supplementary Material 12

Supplementary Material 13

Supplementary Material 2

Supplementary Material 3

Supplementary Material 11

Supplementary Material 4

Supplementary Material 5

Supplementary Material 6

Supplementary Material 7

Supplementary Material 8

Supplementary Material 9

Supplementary Material 14

Supplementary Material 15

Supplementary Material 16

Supplementary Material 17

## Data Availability

The authors confirm that the data supporting the findings of this study are available within the article and its supplementary materials.

## Declarations

### Registration

In compliance with the Joanna Briggs Institute guidelines for conducting a systematic review of prevalence data and Preferred Reporting Items for Systematic Reviews and Meta-Analyses (PRISMA) guidelines, we have registered our systematic review protocol with PROSPERO. PROSPERO ID = CRD42024508438. Our protocol was also published with INPLASY and can be found at https://inplasy.com/inplasy-2024-4-0079/

### Amendments

No amendments were made to the protocol since registration.

### Congress abstracts

Preliminary results from this study were presented as a poster with abstract at the 26th Asia-Pacific League of Associations for Rheumatology Congress (APLAR) held across 21–25 August 2024 and the American College of Rheumatology (ACR) convergence held across 14-19 November 2024. The references for these abstracts are as follows:

### APLAR 2024

Poster Abstracts Part A (2024). International Journal of Rheumatic Diseases. 27(S3):e15345. https://doi.org/10.1111/1756-185X.15345.

### ACR convergence 2024

Prasanna, H., Inderjeeth, C., Nossent, J., & Almutairi, K. (2024). The Global Prevalence of Interstitial Lung Disease in Patients with Rheumatoid Arthritis: A Systematic Review and Meta-analysis. Arthritis Rheumatol, 76(s9). https://acrabstracts.org/abstract/the-global-prevalence-of-interstitial-lung-disease-in-patients-with-rheumatoid-arthritis-a-systematic-review-and-meta-analysis/
