# Supplementary material for: The global prevalence of interstitial lung disease in patients with rheumatoid arthritis: a systematic review and meta-analysis
Source: Rheumatol Int. 2025 Jan 18;45(2):34. doi: 10.1007/s00296-025-05789-4 (PMC11742767; doi:10.1007/s00296-025-05789-4)
Supplement: Supplementary file 2 — Supplementary Material 10 [file 296_2025_5789_MOESM2_ESM.docx]

The global prevalence of interstitial lung disease in patients with rheumatoid arthritis: A systematic review and meta-analysis

Hari Prasanna ^1*^, Charles A Inderjeeth ^1,3^ Johannes C Nossent^1,3^, Khalid B Almutairi1 ^1,2^

**Affiliations**

1 School of Medicine, The University of Western Australia, Perth, Western Australia, Australia

2 Pharmacy Department, King Fahd Specialist Hospital, Burydah, Al Qassim, Saudi Arabia

3 Geronto-Rheumatology, Sir Charles Gairdner and Osborne Park Health Care Group, Perth, Western Australia, Australia

* First and corresponding author: Mr Hari Prasanna

* Corresponding author E-mail: [22981086@student.uwa.edu.au](mailto:22981086@student.uwa.edu.au)

**Address:**

Mr Hari Prasanna

School of Medicine

University of Western Australia

35 Stirling Highway

Perth WA 6009 Australia

**Appendix 2**

A population-based study was included if all the participants met one of the following internationally recognised RA classification criteria sets.

***Table 2: Comparison of the 1987 ACR and 2010 ACR/EULAR classification criteria for diagnosing RA***

| ARA 1987 Criteria | ACR/EULAR 2010 Criteria |
| --- | --- |
| 1. Morning stiffness lasting at least 1 hour | 1. Joint involvement :- 2. 1 large joint = 0 3. 2 – 10 large joints = 1 4. 1 – 3 small joints = 2 5. 4 – 10 small joints = 3 6. >10 joints (at least one small joint) = 5 |
| 1. Arthritis in three or more joint areas | 1. Serology 2. Negative RF and negative ACPA = 0 3. Low positive RF or low positive ACPA = 2 4. High positive RF or high positive ACPA = 3 |
| 1. Arthritis of hand joints (>= 1 swollen joints) | 1. Acute phase reactants 2. Normal CRP and normal ESR = 0 3. Abnormal CRP or abnormal ESR = 1 |
| 1. Symmetrical joint swelling | 1. Duration of symptoms 2. <6 weeks = 0 3. >= 6 weeks = 1 |
| 1. Rheumatoid nodules |  |
| 1. Positive rheumatoid factor |  |
| 1. Radiographic changes consistent with RA |  |
| Four of the seven criteria must be present and criteria 1 – 4 must have been present for at least six weeks | Scoring 6 or more points is required to diagnose RA |

From :-

1. Aletaha D, Neogi T, Silman AJ, Funovits J, Felson DT, Bingham CO, 3rd, et al. 2010 rheumatoid arthritis classification criteria: an American College of Rheumatology/European League Against Rheumatism collaborative initiative. Ann Rheum Dis. 2010;69(9):1580-8.

2. Arnett FC, Edworthy SM, Bloch DA, McShane DJ, Fries JF, Cooper NS, et al. The American Rheumatism Association 1987 revised criteria for the classification of rheumatoid arthritis. Arthritis Rheum. 1988;31(3):315-24.
