# Supplementary material for: The global prevalence of interstitial lung disease in patients with rheumatoid arthritis: a systematic review and meta-analysis
Source: Rheumatol Int. 2025 Jan 18;45(2):34. doi: 10.1007/s00296-025-05789-4 (PMC11742767; doi:10.1007/s00296-025-05789-4)
Supplement: Supplementary file 3 — Supplementary Material 12 [file 296_2025_5789_MOESM3_ESM.docx]

The global prevalence of interstitial lung disease in patients with rheumatoid arthritis: A systematic review and meta-analysis

Hari Prasanna ^1*^, Charles A Inderjeeth ^1,3^ Johannes C Nossent^1,3^, Khalid B Almutairi1 ^1,2^

**Affiliations**

1 School of Medicine, The University of Western Australia, Perth, Western Australia, Australia

2 Pharmacy Department, King Fahd Specialist Hospital, Burydah, Al Qassim, Saudi Arabia

3 Geronto-Rheumatology, Sir Charles Gairdner and Osborne Park Health Care Group, Perth, Western Australia, Australia

* First and corresponding author: Mr Hari Prasanna

* Corresponding author E-mail: [22981086@student.uwa.edu.au](mailto:22981086@student.uwa.edu.au)

**Address:**

Mr Hari Prasanna

School of Medicine

University of Western Australia

35 Stirling Highway

Perth WA 6009 Australia

**Appendix 3**

***Table 3: Radiologic features of Idiopathic Interstitial Pneumonias according to the American Thoracic Society/European Respiratory Society International Multidisciplinary Consensus Classification of the Idiopathic Interstitial Pneumonias.***

| Radiologic Pattern | Usual Radiologic Features | Typical distribution on CT | Typical CT findings |
| --- | --- | --- | --- |
| UIP | Basal-predominant reticular  abnormality with volume loss | Peripheral, subpleural, basal | Reticular, honeycombing  Traction bronchiectasis/  bronchiectasis; architectural  distortion. Focal ground glass |
| NSIP | Ground glass and reticular  opacity | Peripheral, subpleural, basal, symmetric | Ground glass attenuation  Irregular lines, Consolidation |
| OP | Patchy bilateral consolidation | Subpleural/peribronchial | Patchy consolidation and/or  nodules |

*Definition of abbreviations*: UIP = usual interstitial pneumonia; NSIP = nonspecific interstitial pneumonia; OP = organising pneumonia

From Table 3 of :-

*American Thoracic S, European Respiratory S. American Thoracic Society/European Respiratory Society International Multidisciplinary Consensus Classification of the Idiopathic Interstitial Pneumonias. This joint statement of the American Thoracic Society (ATS), and the European Respiratory Society (ERS) was adopted by the ATS board of directors, June 2001 and by the ERS Executive Committee, June 2001. Am J Respir Crit Care Med. 2002;165(2):277-304.*
