# Supplementary material for: The global prevalence of interstitial lung disease in patients with rheumatoid arthritis: a systematic review and meta-analysis
Source: Rheumatol Int. 2025 Jan 18;45(2):34. doi: 10.1007/s00296-025-05789-4 (PMC11742767; doi:10.1007/s00296-025-05789-4)
Supplement: Supplementary file 4 — Supplementary Material 13 [file 296_2025_5789_MOESM4_ESM.docx]

The global prevalence of interstitial lung disease in patients with rheumatoid arthritis: A systematic review and meta-analysis

Hari Prasanna ^1*^, Charles A Inderjeeth ^1,3^ Johannes C Nossent^1,3^, Khalid B Almutairi1 ^1,2^

**Affiliations**

1 School of Medicine, The University of Western Australia, Perth, Western Australia, Australia

2 Pharmacy Department, King Fahd Specialist Hospital, Burydah, Al Qassim, Saudi Arabia

3 Geronto-Rheumatology, Sir Charles Gairdner and Osborne Park Health Care Group, Perth, Western Australia, Australia

* First and corresponding author: Mr Hari Prasanna

* Corresponding author E-mail: [22981086@student.uwa.edu.au](mailto:22981086@student.uwa.edu.au)

**Address:**

Mr Hari Prasanna

School of Medicine

University of Western Australia

35 Stirling Highway

Perth WA 6009 Australia

**Appendix 4**

***Table 4: MEDLINE search strategy:***

| 1. exp Arthritis, Rheumatoid/ |
| --- |
| 1. Rheumatoid Arthritis.tw. |
| 1. RA.tw. |
| 1. 1 or 2 or 3 |
|  |
| 1. exp Lung Diseases, Interstitial/ |
| 1. diffuse parenchymal lung.mp. |
| 1. pulmonary fibrosis.mp. |
| 1. Interstitial lung disease*.mp. |
| 1. Rheumatoid lung.mp. |
| 1. RA-ILD.mp. |
| 1. ILD.mp. |
| 1. UIP.mp. |
| 1. NSIP.mp. |
| 1. Alveolitis.mp. |
| 1. Organi$ing pneumon*.mp. |
| 1. 5 or 6 or 7 or 8 or 9 or 10 or 11 or 12 or 13 or 14 or 15 |
|  |
| 1. exp Prevalence/ |
| 1. Prevalence*.mp. |
| 1. Population-based study.mp. |
| 1. Epidemiology.mp. |
| 1. Trends.mp. |
| 1. Rate.mp. |
| 1. 17 or 18 or 19 or 20 or 21 or 22 |
|  |
| 1. exp tomography, x-ray computed/ or exp computed tomography angiography/ or exp tomography, spiral computed/ |
| 1. HRCT.mp. |
| 1. CT scan.mp. |
| 1. Computed Tomography.mp. |
| 1. 24 or 25 or 26 or 27 |
|  |
| 1. 4 and 16 and 23 and 28 |
| 1. Limit 29 to yr="1980 - 2024" and human and English language |

***Table 5: Embase search strategy:***

| 1. exp rheumatoid arthritis/ |
| --- |
| 1. Rheumatoid Arthritis.tw. |
| 1. RA.tw. |
| 1. 1 or 2 or 3 |
|  |
| 1. exp interstitial lung disease/ |
| 1. Interstitial lung disease*.mp. |
| 1. Diffuse parenchymal lung.mp. |
| 1. Rheumatoid lung.mp. |
| 1. RA-ILD.mp. |
| 1. ILD.mp. |
| 1. UIP.mp. |
| 1. NSIP.mp. |
| 1. Alveolitis.mp. |
| 1. Organi$ing pneumon*.mp. |
| 1. (pulmonary adj2 fibrosis).mp. |
| 1. exp lung fibrosis/ |
| 1. 5 or 6 or 7 or 8 or 9 or 10 or 11 or 12 or 13 or 14 or 15 or 16 |
|  |
| 1. exp Prevalence/ |
| 1. Prevalence*.mp. |
| 1. Population-based study.mp. |
| 1. Epidemiology.mp. |
| 1. Trends.mp. |
| 1. Rate.mp. |
| 1. 18 or 19 or 20 or 21 or 22 or 23 |
|  |
| 1. Computer assisted tomography/ or exp x-ray computed tomography/ or exp high resolution computer tomography/ |
| 1. HRCT.mp. |
| 1. CT scan.mp. |
| 1. Computed Tomography.mp. |
| 1. 25 or 26 or 27 or 28 or 29 or 30 |
|  |
| 1. 4 and 17 and 24 and 31 |
| 1. Limit 30 to yr="1980 - 2024" and article and human and english language |

***Table 6: ProQuest search strategy***

| (MAINSUBJECT.EXACT("Rheumatoid arthritis") OR MESH.EXACT("Arthritis, Rheumatoid") OR ABSTRACT,TITLE(Rheumatoid Arthritis) OR ABSTRACT,TITLE("RA")) AND (MESH.EXACT("Lung Diseases, Interstitial") OR Interstitial lung disease* OR Rheumatoid lung OR RA-ILD OR "ILD" OR "UIP" OR "NSIP" OR Alveolits OR Organi$ing pneumon*) AND (MESH.EXACT("Prevalence") OR Prevalence* OR Epidemiology OR Trends OR Rate OR Population-based study) AND (MESH.EXACT("tomography, x-ray computed") OR MESH.EXACT("computed tomography angiography") OR MESH.EXACT("tomography, spiral computed") OR HRCT OR CT scan OR computed tomography) AND at.exact("Article") AND stype.exact("Scholarly Journals") AND subt.exact("studies" OR "humans" OR "population" OR "prevalence") AND pd(19800101-20240201) AND PEER(yes) AND la.exact("ENG") |
| --- |

***Table 7: Web Of Science search strategy***

| (((((AB=(Rheumatoid Arthritis OR "RA")) AND ALL=(Interstitial lung disease* OR Rheumatoid lung OR RA-ILD OR "ILD" OR "UIP" OR "NSIP" OR Alveolits OR Organi$ing pneumon* OR Pulmonary fibrosis)) AND ALL=(Epidemiology OR Trends OR Rate OR Population-based study OR prevalence)) AND ALL=(HRCT OR CT scan OR computed tomography)) AND DT=(Article)) AND LA=(English)  Limit Publication date to 1980-01-01 and 2024-02-01 |
| --- |

***Table 8: Cinahl search strategy***

| (AB (rheumatoid arthritis or ra ) OR TI (rheumatoid arthritis or ra )) AND TX ( Interstitial lung disease* OR Rheumatoid lung OR RA-ILD OR "ILD" OR "UIP" OR "NSIP" OR Alveolits OR pulmonary fibrosis) AND TX ( Epidemiology OR Trends OR Rate OR Population-based study OR prevalence ) AND TX ( HRCT OR CT scan OR computed tomography ) AND LA english  Publication Date: 19800101-20240201 |
| --- |

***Table 9: Scopus search strategy***

| TITLE-ABS((rheumatoid PRE/2 arthritis) OR ra) AND ALL ( (interstitial PRE/1 lung PRE/1 disease*) OR (rheumatoid PRE/1 lung) OR ra-ild OR "ILD" OR "UIP" OR "NSIP" OR alveolits OR (organi$ing PRE/1 pneumon*) OR (pulmonary AND fibrosis) ) AND ALL ( epidemiology OR trends OR rate OR (population-based PRE/1 study) OR prevalence ) AND ALL ( hrct OR ct AND scan OR (computed PRE/1 tomography) ) AND DOCTYPE ( ar ) AND LANGUAGE(English) |
| --- |

***Table 10: Google Scholar search strategy***

| Find Articles  **With all the words**: Rheumatoid arthritis Interstitial lung disease CT scan  **With at least one of the words**: prevalence rate epidemiology trends population-based  **Return articles dated between**: 1980 – 2024 |
| --- |
