# Supplementary material for: The global prevalence of interstitial lung disease in patients with rheumatoid arthritis: a systematic review and meta-analysis
Source: Rheumatol Int. 2025 Jan 18;45(2):34. doi: 10.1007/s00296-025-05789-4 (PMC11742767; doi:10.1007/s00296-025-05789-4)
Supplement: Supplementary file 5 — Supplementary Material 2 [file 296_2025_5789_MOESM5_ESM.docx]

The global prevalence of interstitial lung disease in patients with rheumatoid arthritis: A systematic review and meta-analysis

Hari Prasanna ^1*^, Charles A Inderjeeth ^1,3^ Johannes C Nossent^1,3^, Khalid B Almutairi1 ^1,2^

**Affiliations**

1 School of Medicine, The University of Western Australia, Perth, Western Australia, Australia

2 Pharmacy Department, King Fahd Specialist Hospital, Burydah, Al Qassim, Saudi Arabia

3 Geronto-Rheumatology, Sir Charles Gairdner and Osborne Park Health Care Group, Perth, Western Australia, Australia

* First and corresponding author: Mr Hari Prasanna

* Corresponding author E-mail: [22981086@student.uwa.edu.au](mailto:22981086@student.uwa.edu.au)

**Address:**

Mr Hari Prasanna

School of Medicine

University of Western Australia

35 Stirling Highway

Perth WA 6009 Australia

**Appendix 5**

***Table 11: Hoy et al. risk of bias assessment tool***


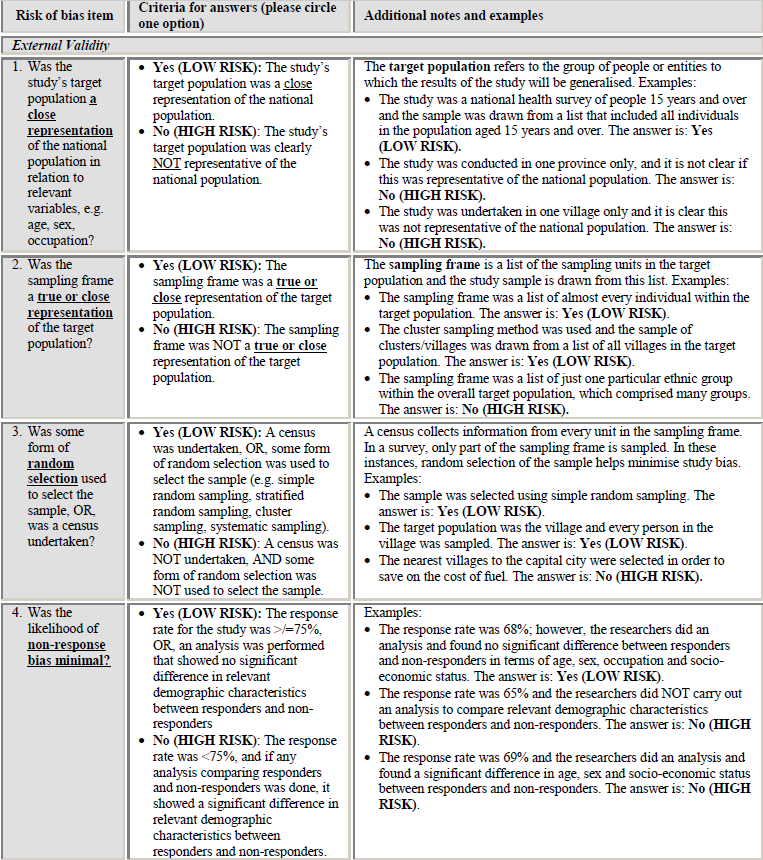

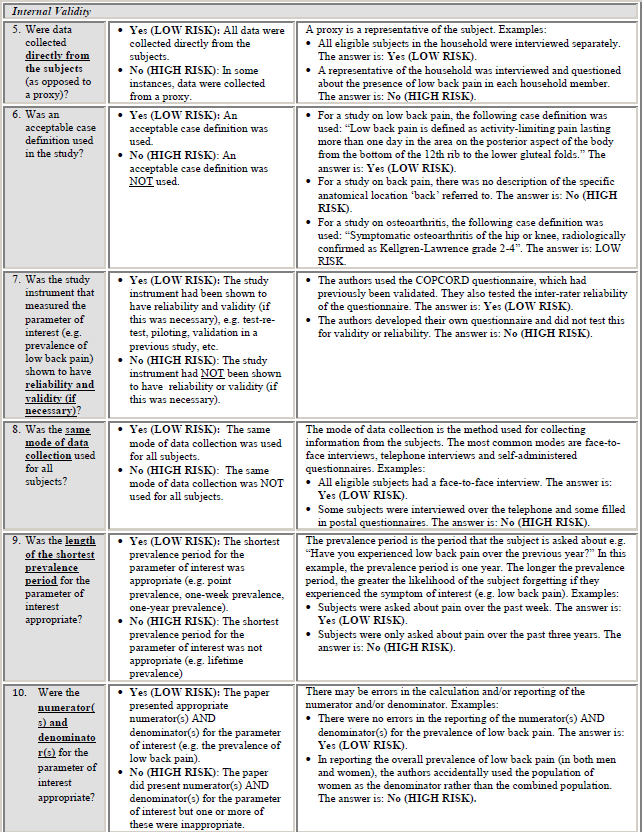


Note: If there is insufficient information in the article to answer a particular item, answer ‘NO’ for that item.

Each item that had a ‘NO’ as an answer is tallied up. If the total number of points are:

- 0 – 3 points = LOW RISK OF BIAS: Further research is very unlikely to change our confidence in the estimate.
- 4 – 6 points = MODERATE RISK OF BIAS: Further research is likely to have an important impact on our confidence in the estimate and may change the estimate.
- 7 – 10 points = HIGH RISK OF BIAS: Further research is very likely to have an important impact on our confidence in the estimate and is likely to change the estimate.

*From: Hoy D, Brooks P, Woolf A, Blyth F, March L, Bain C, et al. Assessing risk of bias in prevalence studies: modification of an existing tool and evidence of interrater agreement. J Clin Epidemiol. 2012;65(9):934-9*
