# Supplementary material for: The global prevalence of interstitial lung disease in patients with rheumatoid arthritis: a systematic review and meta-analysis
Source: Rheumatol Int. 2025 Jan 18;45(2):34. doi: 10.1007/s00296-025-05789-4 (PMC11742767; doi:10.1007/s00296-025-05789-4)
Supplement: Supplementary file 6 — Supplementary Material 3 [file 296_2025_5789_MOESM6_ESM.docx]

The global prevalence of interstitial lung disease in patients with rheumatoid arthritis: A systematic review and meta-analysis

Hari Prasanna ^1*^, Charles A Inderjeeth ^1,3^ Johannes C Nossent^1,3^, Khalid B Almutairi1 ^1,2^

**Affiliations**

1 School of Medicine, The University of Western Australia, Perth, Western Australia, Australia

2 Pharmacy Department, King Fahd Specialist Hospital, Burydah, Al Qassim, Saudi Arabia

3 Geronto-Rheumatology, Sir Charles Gairdner and Osborne Park Health Care Group, Perth, Western Australia, Australia

* First and corresponding author: Mr Hari Prasanna

* Corresponding author E-mail: [22981086@student.uwa.edu.au](mailto:22981086@student.uwa.edu.au)

**Address:**

Mr Hari Prasanna

School of Medicine

University of Western Australia

35 Stirling Highway

Perth WA 6009 Australia

**Appendix 6**

**Table 12: Study characteristics of the 33 included studies**

| Author | Year of study | Study Design |  | Country of study | RA Classification criteria | Sampling Methodology | Sample size | Prevalence method |  | Prevalence of ILD | Risk of bias assessment |
| --- | --- | --- | --- | --- | --- | --- | --- | --- | --- | --- | --- |
|  |  |  | Socioeconomic status |  |  |  |  |  | Time period of recruitment |  |  |
| Gabbay et al. [1] | 1997 | Prospective cohort | High Income | Australia | 1987 ARA | Sampled | 36 | PointP | N/A | 0.3333 | Low |
| Koduri et al. [2] | 2010 | Retrospective cohort | High Income | England | 1987 ARA | Patient records | 1460 | PeriodP | 1986 - 1998 | 0.0356 | Low |
| Mori et al. [3] | 2011 | Prospective cohort | High Income | Japan | 1987 ARA | Sampled | 189 | PeriodP | Jan 2009 - May 2009 | 0.1005 | Low |
| Zou et al. [4] | 2012 | Prospective cohort | Upper Middle Income | China | 1987 ARA | Sampled | 110 | PeriodP | Dec 2008 - Nov 2009 | 0.4273 | Low |
| Giles et al. [5] | 2014 | Prospective cohort | High Income | USA | 1987 ARA | Sampled | 176 | PeriodP | Oct 2004 - May 2006 | 0.3295 | Low |
| Wang et al. [6] | 2015 | Retrospective cohort | Upper Middle Income | China | 1987 ARA | Patient records | 544 | PeriodP | Juy 2006 - June 2011 | 0.1526 | Low |
| Chen et al. USA [7] | 2015 | Prospective cohort | High Income | USA | 1987 ARA | Sampled | 86 | PeriodP | October 2010–June 2013 | 0.5698 | Low |
| Chen et al. China [7] | 2015 | Prospective cohort | Upper Middle Income | China | 1987 ARA | Sampled | 133 | PeriodP | July 2012 - March 2013 | 0.3083 | Low |
| Okada et al. [8] | 2016 | Retrospective cohort | High Income | Japan | 1987 ARA | Patient records | 499 | PointP | Dec 2010 | 0.1680 | Moderate |
| Song et al. [9] | 2016 | Prospective cohort | High Income | South Korea | 1987 ARA | Sampled | 116 | PeriodP | Sep 2013 - May 2014 | 0.0690 | Low |
| Kim et al. [10] | 2017 | Retrospective cohort | High Income | South Korea | 1987 ARA | Patient records | 244 | PeriodP | July 2009 - Dec 2012 | 0.098 | Low |
| Fadda et al. [11] | 2018 | Cross-sectional study | Lower Middle Income | Egypt | 2010 ACR/EULAR | Sampled | 88 | PointP | N/A | 0.716 | Low |
| Salaffi et al. [12] | 2019 | Retrospective cohort | High Income | Italy | 2010 ACR/EULAR | Patient records | 151 | PeriodP | Jan 2014 - Jun 2018 | 0.1921 | Moderate |
| Li et al. [13] | 2019 | Retrospective cohort | Upper Middle Income | China | 1987 ARA or 2010 ACR/EULAR | Patient records | 1096 | PeriodP | Oct 2008 - Oct 2017 | 0.3841 | Moderate |
| Sherin et al. [14] | 2019 | Cross-sectional study | Lower Middle Income | Egypt | 2010 ACR/EULAR | Sampled | 100 | PointP | N/A | 0.36 | Low |
| Manfredi et al. [15] | 2019 | Prospective cohort | High Income | Italy | 1987 ARA or 2010 ACR/EULAR | Sampled | 137 | PointP | N/A | 0.4307 | Low |
| Fu et al. [16] | 2019 | Retrospective cohort | Upper Middle Income | China | 1987 ARA or 2010 ACR/EULAR | Patient records | 791 | PeriodP | May 2008 - Jan 2014 | 0.3881 | Low |
| England et al. [17] | 2019 | Retrospective cohort | High Income | USA | 1987 ACR | Patient records | 1823 | PeriodP | N/A | 0.0494 | Low |
| Gautam et al. [18] | 2020 | Prospective cohort | Lower Middle Income | India | 2010 ACR/EULAR | Sampled | 54 | PeriodP | Jun 2014 - Jun 2015 | 0.3704 | Low |
| Castellanos-moreira et al. [19] | 2020 | Cross-sectional study | High Income | Spain | 2010 ACR/EULAR | Sampled | 148 | PeriodP | July 2017 - july 2018 | 0.0405 | Low |
| Li L et al. [20] | 2020 | Retrospective cohort | Upper Middle Income | China | 1987 ARA or 2010 ACR/EULAR | Patient records | 923 | PeriodP | May 2008 - Oct 2017 | 0.3012 | Low |
| Wickrematilake et al. [21] | 2021 | Prospective cohort | Lower Middle Income | Srilanka | 1987 ARA or 2010 ACR/EULAR | Sampled | 384 | PointP | N/A | 0.1458 | Low |
| Paulin et al. [22] | 2021 | Prospective cohort | Upper Middle Income | Argentina | 2010 ACR/EULAR | Sampled | 79 | PeriodP | Dec 2017 - Feb 2020 | 0.0759 | Low |
| Liang et al. Discovery cohort [23] | 2021 | Prospective | Upper Middle Income | China | 2010 ACR/EULAR | Sampled | 70 | PointP | Jan 2020 - Sep 2020 | 0.2 | Low |
| Liang et al. Identification cohort [23] | 2021 | Retrospective | Upper Middle Income | China | 2010 ACR/EULAR | Sampled | 98 | PointP | Jan 2020 - Oct 2020 | 0.3469 | Low |
| Samhouri et al. [24] | 2022 | Retrospective cohort | High Income | USA | 1987 ACR | Patient records | 623 | PeriodP | 1999 - 2014 | 0.0819 | Low |
| Gutierrez et al. [25] | 2022 | Prospective cohort | Upper Middle Income | Mexico and Argentina | 2010 ACR/EULAR | Sampled | 74 | PointP | N/A | 0.3649 | Low |
| Bonilla Hernan et al. [26] | 2022 | Prospective cohort | High Income | Spain | 1987 ARA or 2010 ACR/EULAR | Sampled | 2729 | PeriodP | 2007 - 2018 | 0.0330 | Low |
| Denis A et al.[27] | 2022 | Retrospective cohort | High Income | Belgium | 2010 ACR/EULAR | Patient records | 523 | PeriodP | Jan 2010 - Jan 2020 | 0.1702 | Low |
| Severo et al. [28] | 2022 | Retrospective cross-sectional | Upper Middle Income | Brazil | 2010 ACR/EULAR | Patient records | 134 | PeriodP | Mar 2019 - Dec 2019 | 0.3657 | Low |
| Abdelwahab et al. [29] | 2022 | Cross-sectional study | Lower Middle Income | Egypt | 2010 ACR/EULAR | Sampled | 30 | PeriodP | Nov 2018 - Nov 2019 | 0.733 | Low |
| Sanaa et al. [30] | 2023 | Prospective cohort | Lower Middle Income | Tanzania | 2010 ACR/EULAR | Sampled | 132 | PeriodP | N/A | 0.0303 | Low |
| Razmjou et al. [31] | 2023 | Retrospective cohort | High Income | USA | 1987 ARA | Patient records | 108 | PeriodP | May 2021 | 0.1481 | Low |
| Ren et al. [32] | 2023 | Retrospective cohort | Upper Middle Income | China | 2010 ACR/EULAR | Patient records | 154 | PeriodP | Jan 2012 - Aug 2021 | 0.435 | Moderate |
| Yu et al. [33] | 2023 | Retrospective cohort | Upper Middle Income | China | 1987 ARA | Patient records | 239 | PeriodP | 2019 - 2021 | 0.251 | Moderate |

*PeriodP = Period Prevalence, PointP = Point Prevalence*

1. Gabbay E, Tarala R, Will R, Carroll C, Adler B, Cameron D, Lake FR. Interstitial lung disease in recent onset rheumatoid arthritis. AMERICAN JOURNAL OF RESPIRATORY AND CRITICAL CARE MEDICINE. 1997;156(2):528-35.

2. Koduri G, Norton S, Young A, Cox N, Davies P, Devlin J, et al. Interstitial lung disease has a poor prognosis in rheumatoid arthritis: results from an inception cohort. RHEUMATOLOGY. 2010;49(8):1483-9.

3. Mori S, Koga Y, Sugimoto M. Small airway obstruction in patients with rheumatoid arthritis. Modern Rheumatology. 2011;21(2):164-73.

4. Zou YQ, Li YS, Ding XN, Ying ZH. The clinical significance of HRCT in evaluation of patients with rheumatoid arthritis-associated interstitial lung disease: A report from China. Rheumatology International. 2012;32(3):669-73.

5. Giles JT, Darrah E, Danoff S, Johnson C, Andrade F, Rosen A, Bathon JM. Association of cross-reactive antibodies targeting peptidyl-arginine deiminase 3 and 4 with rheumatoid arthritis-associated interstitial lung disease. PLoS ONE. 2014;9(6):e98794.

6. Wang JX, Du CG. A retrospective study of clinical characteristics of interstitial lung disease associated with rheumatoid arthritis in Chinese patients. Medical Science Monitor. 2015;21:708-15.

7. Chen J, Doyle TJ, Liu Y, Aggarwal R, Wang X, Shi Y, et al. Biomarkers of rheumatoid arthritis-associated interstitial lung disease. Arthritis and Rheumatology. 2015;67(1):28-38.

8. Okada H, Kurasawa K, Yamazaki R, Tanaka A, Arai S, Owada T, et al. Clinical features of organizing pneumonia associated with rheumatoid arthritis. Modern rheumatology. 2016;26(6):863-8.

9. Song ST, Kim SS, Kim JY, Lee SY, Kim K, Kwon IS, et al. Association of Single Nucleotide Polymorphisms of PADI4 and HLA-DRB1 Alleles with Susceptibility to Rheumatoid Arthritis-Related Lung Diseases. Lung. 2016;194(5):745-53.

10. Kim D, Cho S-K, Choi C-B, Choe J-Y, Chung WT, Hong S-J, et al. Impact of interstitial lung disease on mortality of patients with rheumatoid arthritis. Rheumatology international. 2017;37(10):1735-45.

11. Fadda S, Khairy N, Fayed H, Mousa H, Taha R. Interstitial lung disease in Egyptian patients with rheumatoid arthritis: Frequency, pattern and correlation with clinical manifestations and anti-citrullinated peptide antibodies level. Egyptian Rheumatologist. 2018;40(3):155-60.

12. Salaffi F, Carotti M, Di Carlo M, Tardella M, Giovagnoni A. High-resolution computed tomography of the lung in patients with rheumatoid arthritis: Prevalence of interstitial lung disease involvement and determinants of abnormalities. Medicine. 2019;98(38):e17088.

13. Li L, Gao S, Fu Q, Liu R, Zhang Y, Dong X, et al. A preliminary study of lung abnormalities on HRCT in patients of rheumatoid arthritis–associated interstitial lung disease with progressive fibrosis. Clinical Rheumatology. 2019;38(11):3169-78.

14. Sherin H, Dalia E, Haytham D, Takwa Y. Vitamin D deficiency and pulmonary affection in rheumatoid arthritis. Egyptian Journal of Chest Diseases and Tuberculosis. 2019;68(4):614-23.

15. Manfredi A, Cassone G, Cerri S, Venerito V, Fedele AL, Trevisani M, et al. Diagnostic accuracy of a velcro sound detector (VECTOR) for interstitial lung disease in rheumatoid arthritis patients: The InSPIRAtE validation study (INterStitial pneumonia in rheumatoid ArThritis with an electronic device). BMC Pulmonary Medicine. 2019;19(1).

16. Fu Q, Wang L, Li LL, Li YF, Liu R, Zheng Y. Risk factors for progression and prognosis of rheumatoid arthritis-associated interstitial lung disease: single center study with a large sample of Chinese population. CLINICAL RHEUMATOLOGY. 2019;38(4):1109-16.

17. England BR, Duryee MJ, Roul P, Mahajan TD, Singh N, Poole JA, et al. Malondialdehyde–Acetaldehyde Adducts and Antibody Responses in Rheumatoid Arthritis–Associated Interstitial Lung Disease. Arthritis and Rheumatology. 2019;71(9):1483-93.

18. Gautam M, Masood MJ, Arooj S, Mahmud MEH, Mukhtar MU. Rheumatoid Arthritis Related Interstitial Lung Disease: Patterns of High-resolution Computed Tomography. CUREUS JOURNAL OF MEDICAL SCIENCE. 2020;12(2).

19. Castellanos-Moreira R, Rodríguez-García SC, Gomara MJ, Ruiz-Esquide V, Cuervo A, Casafont-Solé I, et al. Anti-carbamylated proteins antibody repertoire in rheumatoid arthritis: evidence of a new autoantibody linked to interstitial lung disease. Annals of the Rheumatic Diseases. 2020;79(5):587-94.

20. Li L, Liu R, Zhang Y, Zhou J, Li Y, Xu Y, et al. A retrospective study on the predictive implications of clinical characteristics and therapeutic management in patients with rheumatoid arthritis-associated interstitial lung disease. Clinical rheumatology. 2020;39(5):1457-70.

21. Wickrematilake G. Interstitial Lung Disease and its Associations in Rheumatoid Arthritis: Data from a District General Hospital in Sri Lanka. Clinical Medicine Insights: Arthritis and Musculoskeletal Disorders. 2021;14.

22. Paulin F, Secco A, Benavidez F, Moncalvo JJR, Carballo OG, Ingenito F, et al. Lung involvement prevalence in patients with early rheumatoid arthritis without known pulmonary disease: a multicentric cross sectional study. ADVANCES IN RHEUMATOLOGY. 2021;61(1).

23. Liang L, Chen JL, Di C, Zhan MH, Bao HZ, Xia CS, et al. Serum Human Epididymis Protein 4 as a Novel Biomarker in Identifying Patients With Interstitial Lung Disease in Rheumatoid Arthritis. FRONTIERS IN MEDICINE. 2021;8.

24. Samhouri BF, Vassallo R, Achenbach SJ, Kronzer VL, Davis JM, Myasoedova E, Crowson CS. Incidence, Risk Factors, and Mortality of Clinical and Subclinical Rheumatoid Arthritis–Associated Interstitial Lung Disease: A Population-Based Cohort. Arthritis Care and Research. 2022;74(12):2042-9.

25. Gutierrez M, Ruta S, Clavijo-Cornejo D, Fuentes-Moreno G, Reyes-Long S, Bertolazzi C. The emerging role of ultrasound in detecting interstitial lung disease in patients with rheumatoid arthritis. Joint Bone Spine. 2022;89(6).

26. Bonilla Hernan MG, Gomez-Carrera L, Fernandez-Velilla Pena M, Alvarez-Sala Walther R, Balsa A. Prevalence and clinical characteristics of symptomatic diffuse interstitial lung disease in rheumatoid arthritis in a Spanish population. Revista Clinica Espanola. 2022;222(5):281-7.

27. Denis A, Henket M, Ernst M, Maes N, Thys M, Regnier C, et al. Progressive fibrosing interstitial lung disease in rheumatoid arthritis: A retrospective study. Frontiers in Medicine. 2022;9:1024298.

28. Severo CR, Chomiski C, do Valle MB, Escuissato DL, Paiva ED, Storrer KM. Assessment of risk factors in patients with rheumatoid arthritis-associated interstitial lung disease. JORNAL BRASILEIRO DE PNEUMOLOGIA. 2022;48(6).

29. Abdelwahab HW, Shalabi NM, Ghoneim MMR, Farrag NS, Hamdy F, Elhoseiny F, Ali RE. Screening for Subclinical Interstitial Lung Disease in Rheumatoid Arthritis Patients: Functional and Radiological Methods. Turkish Thoracic Journal. 2022;23(4):261-7.

30. Sanaa S, Noorein O, Faiza J, Clive K. Clinical, serological and radiological findings in patients with rheumatoid arthritis from Zanzibar comparing those with and without interstitial lung disease. Trends in Immunotherapy. 2023;7(2):2716.

31. Razmjou AA, Wang JM, Shahbazian A, Reddy S, Charles-Schoeman C. Suppressed paraoxonase-1 activity associates with elevated oxylipins and the presence of small airways disease in patients with rheumatoid arthritis. Clinical Rheumatology. 2023;42(1):75-82.

32. Ren JQ, Ding YL, Zhao JX, Sun YC. Impact of cigarette smoking on rheumatoid arthritis-associated lung diseases: a retrospective case control study on clinical and radiological features and prognosis. RHEUMATOLOGY INTERNATIONAL. 2023;43(2):293-301.

33. Yu R, Liu XM, Deng XY, Li ST, Wang YF, Zhang Y, et al. Serum CHI3L1 as a biomarker of interstitial lung disease in rheumatoid arthritis. FRONTIERS IN IMMUNOLOGY. 2023;14.
