# Supplementary material for: The global prevalence of interstitial lung disease in patients with rheumatoid arthritis: a systematic review and meta-analysis
Source: Rheumatol Int. 2025 Jan 18;45(2):34. doi: 10.1007/s00296-025-05789-4 (PMC11742767; doi:10.1007/s00296-025-05789-4)
Supplement: Supplementary file 7 — Supplementary Material 11 [file 296_2025_5789_MOESM7_ESM.docx]

The global prevalence of interstitial lung disease in patients with rheumatoid arthritis: A systematic review and meta-analysis

Hari Prasanna ^1*^, Charles A Inderjeeth ^1,3^ Johannes C Nossent^1,3^, Khalid B Almutairi1 ^1,2^

**Affiliations**

1 School of Medicine, The University of Western Australia, Perth, Western Australia, Australia

2 Pharmacy Department, King Fahd Specialist Hospital, Burydah, Al Qassim, Saudi Arabia

3 Geronto-Rheumatology, Sir Charles Gairdner and Osborne Park Health Care Group, Perth, Western Australia, Australia

* First and corresponding author: Mr Hari Prasanna

* Corresponding author E-mail: [22981086@student.uwa.edu.au](mailto:22981086@student.uwa.edu.au)

**Address:**

Mr Hari Prasanna

School of Medicine

University of Western Australia

35 Stirling Highway

Perth WA 6009 Australia

**Appendix 7**

***Table 13: The risk of bias assessment scoring for the 33 included studies***

| Citations | Q1 | Q2 | Q3 | Q4 | Q5 | Q6 | Q7 | Q8 | Q9 | Q10 | Risk of bias score |
| --- | --- | --- | --- | --- | --- | --- | --- | --- | --- | --- | --- |
| Gabbay et al. [1] | N | Y | N | Y | Y | Y | Y | Y | Y | Y | Low |
| Mori et al. [2] | N | Y | N | Y | Y | Y | Y | Y | Y | Y | Low |
| Zou et al. [3] | N | Y | N | Y | Y | Y | Y | Y | Y | Y | Low |
| Giles et al. [4] | N | Y | N | Y | Y | Y | Y | Y | N | Y | Low |
| Wang et al. [5] | N | Y | N | Y | N | Y | Y | Y | N | Y | Low |
| Okada et al. [6] | N | Y | N | Y | N | Y | Y | N | Y | Y | Moderate |
| Kim et al. [7] | Y | Y | Y | Y | N | Y | Y | N | N | Y | Low |
| Salaffi et al. [8] | N | Y | N | Y | N | Y | Y | Y | N | Y | Moderate |
| Li et al. [9] | N | Y | N | Y | N | Y | Y | Y | N | Y | Moderate |
| Sherin et al. [10] | N | Y | N | Y | Y | Y | Y | Y | Y | Y | Low |
| Wickrematilake et al. [11] | N | Y | N | Y | Y | Y | Y | Y | Y | Y | Low |
| Paulin et al. [12] | N | Y | N | Y | Y | Y | Y | Y | N | Y | Low |
| Liang et al. [13] | N | Y | N | Y | Y | Y | Y | Y | Y | Y | Low |
| Samhouri et al. [14] | N | Y | Y | Y | N | Y | Y | Y | N | Y | Low |
| Gutierrez et al. [15] | N | Y | N | Y | Y | Y | Y | Y | Y | Y | Low |
| Bonilla Hernan et al. [16] | N | Y | Y | Y | Y | Y | Y | Y | N | Y | Low |
| Denis A et al. [17] | N | Y | Y | Y | N | Y | Y | Y | N | Y | Low |
| Severo et al. [18] | N | Y | Y | Y | N | Y | Y | Y | Y | Y | Low |
| Sanaa et al. [19] | N | Y | Y | N | Y | Y | Y | Y | Y | Y | Low |
| Abdelwahab et al. [20] | N | Y | N | Y | Y | Y | Y | Y | Y | Y | Low |
| Manfredi et al. [21] | N | Y | N | Y | Y | Y | Y | Y | Y | Y | Low |
| Song et al. [22] | N | Y | N | Y | Y | Y | Y | Y | Y | Y | Low |
| Ren et al. [23] | N | Y | N | Y | N | Y | Y | Y | N | Y | Moderate |
| Yu et al. [24] | N | Y | N | Y | N | Y | Y | Y | N | Y | Moderate |
| Castellanos-moreira et al. [25] | N | Y | N | Y | Y | Y | Y | Y | Y | Y | Low |
| Fadda et al. [26] | N | Y | N | Y | Y | Y | Y | Y | Y | Y | Low |
| Li L et al. [27] | N | Y | Y | Y | N | Y | Y | Y | N | Y | Low |
| Chen et al. [28] | N | Y | N | Y | Y | Y | Y | Y | N | Y | Low |
| Fu et al. [29] | N | Y | Y | Y | Y | Y | Y | Y | N | Y | Low |
| Gautam et al. [30] | N | Y | N | Y | Y | Y | Y | Y | Y | Y | Low |
| Koduri et al. [31] | Y | Y | N | Y | N | Y | Y | Y | N | Y | Low |
| England et al. [32] | Y | Y | N | Y | N | Y | Y | Y | N | Y | Low |
| Razmjou et al. [33] | N | Y | N | Y | Y | Y | Y | Y | N | Y | Low |

**References :-**

1. Gabbay E, Tarala R, Will R, Carroll C, Adler B, Cameron D, Lake FR. Interstitial lung disease in recent onset rheumatoid arthritis. AMERICAN JOURNAL OF RESPIRATORY AND CRITICAL CARE MEDICINE. 1997;156(2):528-35.

2. Mori S, Koga Y, Sugimoto M. Small airway obstruction in patients with rheumatoid arthritis. Modern Rheumatology. 2011;21(2):164-73.

3. Zou YQ, Li YS, Ding XN, Ying ZH. The clinical significance of HRCT in evaluation of patients with rheumatoid arthritis-associated interstitial lung disease: A report from China. Rheumatology International. 2012;32(3):669-73.

4. Giles JT, Darrah E, Danoff S, Johnson C, Andrade F, Rosen A, Bathon JM. Association of cross-reactive antibodies targeting peptidyl-arginine deiminase 3 and 4 with rheumatoid arthritis-associated interstitial lung disease. PLoS ONE. 2014;9(6):e98794.

5. Wang JX, Du CG. A retrospective study of clinical characteristics of interstitial lung disease associated with rheumatoid arthritis in Chinese patients. Medical Science Monitor. 2015;21:708-15.

6. Okada H, Kurasawa K, Yamazaki R, Tanaka A, Arai S, Owada T, et al. Clinical features of organizing pneumonia associated with rheumatoid arthritis. Modern rheumatology. 2016;26(6):863-8.

7. Kim D, Cho S-K, Choi C-B, Choe J-Y, Chung WT, Hong S-J, et al. Impact of interstitial lung disease on mortality of patients with rheumatoid arthritis. Rheumatology international. 2017;37(10):1735-45.

8. Salaffi F, Carotti M, Di Carlo M, Tardella M, Giovagnoni A. High-resolution computed tomography of the lung in patients with rheumatoid arthritis: Prevalence of interstitial lung disease involvement and determinants of abnormalities. Medicine. 2019;98(38):e17088.

9. Li L, Gao S, Fu Q, Liu R, Zhang Y, Dong X, et al. A preliminary study of lung abnormalities on HRCT in patients of rheumatoid arthritis–associated interstitial lung disease with progressive fibrosis. Clinical Rheumatology. 2019;38(11):3169-78.

10. Sherin H, Dalia E, Haytham D, Takwa Y. Vitamin D deficiency and pulmonary affection in rheumatoid arthritis. Egyptian Journal of Chest Diseases and Tuberculosis. 2019;68(4):614-23.

11. Wickrematilake G. Interstitial Lung Disease and its Associations in Rheumatoid Arthritis: Data from a District General Hospital in Sri Lanka. Clinical Medicine Insights: Arthritis and Musculoskeletal Disorders. 2021;14.

12. Paulin F, Secco A, Benavidez F, Moncalvo JJR, Carballo OG, Ingenito F, et al. Lung involvement prevalence in patients with early rheumatoid arthritis without known pulmonary disease: a multicentric cross sectional study. ADVANCES IN RHEUMATOLOGY. 2021;61(1).

13. Liang L, Chen JL, Di C, Zhan MH, Bao HZ, Xia CS, et al. Serum Human Epididymis Protein 4 as a Novel Biomarker in Identifying Patients With Interstitial Lung Disease in Rheumatoid Arthritis. FRONTIERS IN MEDICINE. 2021;8.

14. Samhouri BF, Vassallo R, Achenbach SJ, Kronzer VL, Davis JM, Myasoedova E, Crowson CS. Incidence, Risk Factors, and Mortality of Clinical and Subclinical Rheumatoid Arthritis–Associated Interstitial Lung Disease: A Population-Based Cohort. Arthritis Care and Research. 2022;74(12):2042-9.

15. Gutierrez M, Ruta S, Clavijo-Cornejo D, Fuentes-Moreno G, Reyes-Long S, Bertolazzi C. The emerging role of ultrasound in detecting interstitial lung disease in patients with rheumatoid arthritis. Joint Bone Spine. 2022;89(6).

16. Bonilla Hernan MG, Gomez-Carrera L, Fernandez-Velilla Pena M, Alvarez-Sala Walther R, Balsa A. Prevalence and clinical characteristics of symptomatic diffuse interstitial lung disease in rheumatoid arthritis in a Spanish population. Revista Clinica Espanola. 2022;222(5):281-7.

17. Denis A, Henket M, Ernst M, Maes N, Thys M, Regnier C, et al. Progressive fibrosing interstitial lung disease in rheumatoid arthritis: A retrospective study. Frontiers in Medicine. 2022;9:1024298.

18. Severo CR, Chomiski C, do Valle MB, Escuissato DL, Paiva ED, Storrer KM. Assessment of risk factors in patients with rheumatoid arthritis-associated interstitial lung disease. JORNAL BRASILEIRO DE PNEUMOLOGIA. 2022;48(6).

19. Sanaa S, Noorein O, Faiza J, Clive K. Clinical, serological and radiological findings in patients with rheumatoid arthritis from Zanzibar comparing those with and without interstitial lung disease. Trends in Immunotherapy. 2023;7(2):2716.

20. Abdelwahab HW, Shalabi NM, Ghoneim MMR, Farrag NS, Hamdy F, Elhoseiny F, Ali RE. Screening for Subclinical Interstitial Lung Disease in Rheumatoid Arthritis Patients: Functional and Radiological Methods. Turkish Thoracic Journal. 2022;23(4):261-7.

21. Manfredi A, Cassone G, Cerri S, Venerito V, Fedele AL, Trevisani M, et al. Diagnostic accuracy of a velcro sound detector (VECTOR) for interstitial lung disease in rheumatoid arthritis patients: The InSPIRAtE validation study (INterStitial pneumonia in rheumatoid ArThritis with an electronic device). BMC Pulmonary Medicine. 2019;19(1).

22. Song ST, Kim SS, Kim JY, Lee SY, Kim K, Kwon IS, et al. Association of Single Nucleotide Polymorphisms of PADI4 and HLA-DRB1 Alleles with Susceptibility to Rheumatoid Arthritis-Related Lung Diseases. Lung. 2016;194(5):745-53.

23. Ren JQ, Ding YL, Zhao JX, Sun YC. Impact of cigarette smoking on rheumatoid arthritis-associated lung diseases: a retrospective case control study on clinical and radiological features and prognosis. RHEUMATOLOGY INTERNATIONAL. 2023;43(2):293-301.

24. Yu R, Liu XM, Deng XY, Li ST, Wang YF, Zhang Y, et al. Serum CHI3L1 as a biomarker of interstitial lung disease in rheumatoid arthritis. FRONTIERS IN IMMUNOLOGY. 2023;14.

25. Castellanos-Moreira R, Rodríguez-García SC, Gomara MJ, Ruiz-Esquide V, Cuervo A, Casafont-Solé I, et al. Anti-carbamylated proteins antibody repertoire in rheumatoid arthritis: evidence of a new autoantibody linked to interstitial lung disease. Annals of the Rheumatic Diseases. 2020;79(5):587-94.

26. Fadda S, Khairy N, Fayed H, Mousa H, Taha R. Interstitial lung disease in Egyptian patients with rheumatoid arthritis: Frequency, pattern and correlation with clinical manifestations and anti-citrullinated peptide antibodies level. Egyptian Rheumatologist. 2018;40(3):155-60.

27. Li L, Liu R, Zhang Y, Zhou J, Li Y, Xu Y, et al. A retrospective study on the predictive implications of clinical characteristics and therapeutic management in patients with rheumatoid arthritis-associated interstitial lung disease. Clinical rheumatology. 2020;39(5):1457-70.

28. Chen J, Doyle TJ, Liu Y, Aggarwal R, Wang X, Shi Y, et al. Biomarkers of rheumatoid arthritis-associated interstitial lung disease. Arthritis and Rheumatology. 2015;67(1):28-38.

29. Fu Q, Wang L, Li LL, Li YF, Liu R, Zheng Y. Risk factors for progression and prognosis of rheumatoid arthritis-associated interstitial lung disease: single center study with a large sample of Chinese population. CLINICAL RHEUMATOLOGY. 2019;38(4):1109-16.

30. Gautam M, Masood MJ, Arooj S, Mahmud MEH, Mukhtar MU. Rheumatoid Arthritis Related Interstitial Lung Disease: Patterns of High-resolution Computed Tomography. CUREUS JOURNAL OF MEDICAL SCIENCE. 2020;12(2).

31. Koduri G, Norton S, Young A, Cox N, Davies P, Devlin J, et al. Interstitial lung disease has a poor prognosis in rheumatoid arthritis: results from an inception cohort. RHEUMATOLOGY. 2010;49(8):1483-9.

32. England BR, Duryee MJ, Roul P, Mahajan TD, Singh N, Poole JA, et al. Malondialdehyde–Acetaldehyde Adducts and Antibody Responses in Rheumatoid Arthritis–Associated Interstitial Lung Disease. Arthritis and Rheumatology. 2019;71(9):1483-93.

33. Razmjou AA, Wang JM, Shahbazian A, Reddy S, Charles-Schoeman C. Suppressed paraoxonase-1 activity associates with elevated oxylipins and the presence of small airways disease in patients with rheumatoid arthritis. Clinical Rheumatology. 2023;42(1):75-82.
