# Supplementary material for: The global prevalence of interstitial lung disease in patients with rheumatoid arthritis: a systematic review and meta-analysis
Source: Rheumatol Int. 2025 Jan 18;45(2):34. doi: 10.1007/s00296-025-05789-4 (PMC11742767; doi:10.1007/s00296-025-05789-4)
Supplement: Supplementary file 8 — Supplementary Material 4 [file 296_2025_5789_MOESM8_ESM.docx]

The global prevalence of interstitial lung disease in patients with rheumatoid arthritis: A systematic review and meta-analysis

Hari Prasanna ^1*^, Charles A Inderjeeth ^1,3^ Johannes C Nossent^1,3^, Khalid B Almutairi1 ^1,2^

**Affiliations**

1 School of Medicine, The University of Western Australia, Perth, Western Australia, Australia

2 Pharmacy Department, King Fahd Specialist Hospital, Burydah, Al Qassim, Saudi Arabia

3 Geronto-Rheumatology, Sir Charles Gairdner and Osborne Park Health Care Group, Perth, Western Australia, Australia

* First and corresponding author: Mr Hari Prasanna

* Corresponding author E-mail: [22981086@student.uwa.edu.au](mailto:22981086@student.uwa.edu.au)

**Address:**

Mr Hari Prasanna

School of Medicine

University of Western Australia

35 Stirling Highway

Perth WA 6009 Australia

**Appendix 8**

**Excluded studies (n=124) and reason for exclusion**

1. Abel A, Lazaro E, Ralazamahaleo M, Pierrisnard E, Suzon B, Bonnet F, et al. Phenotypic Profiles Among 72 Caucasian and Afro-Caribbean Patients with Antisynthetase Syndrome Involving Anti-PL7 or Anti-PL12 Autoantibodies. European Journal of Internal Medicine. 2023;115:104-13.

**Reason for exclusion**: - Wrong Condition

2. Affara NK, Refaat AM, Elgawish MH, Zakaria MA, Dashti KA. High-resolution CT and pulmonary function tests in rheumatoid arthritis patients with subclinical interstitial lung disease in Kuwait. The Egyptian Rheumatologist. 2016;38(2):77-83.

**Reason for exclusion**: - Background Article

3. Akiyama M, Kaneko Y, Yamaoka K, Kondo H, Takeuchi T. Association of disease activity with acute exacerbation of interstitial lung disease during tocilizumab treatment in patients with rheumatoid arthritis: a retrospective, case–control study. Rheumatology international. 2016;36(6):881-9.

**Reason for exclusion:** - Background Article

4. Alamoudi O, Attar S. Pleuropulmonary manifestation in patients with rheumatoid arthritis in Saudi Arabia. Annals of Thoracic Medicine. 2017;12(4).

**Reason for exclusion:** - Prevalence not reported

5. Allam AH, Youssef SMA, Moussa HH, Ezzat Y. Anti-citrullinated peptide antibodies with interstitial lung disease in patients with rheumatoid arthritis. Egyptian Journal of Chest Diseases and Tuberculosis. 2020;69(1):171-7.

**Reason for exclusion:** - Case series/reviews

6. Amital A, Shitrit D, Adir Y. The lung in rheumatoid arthritis. La Presse Médicale. 2011;40(1):e53-e70.

**Reason for exclusion:** - Case series/reviews

7. Anand R, Jacobi BS, Rao BJ. A STUDY ON PULMONARY INVOLVEMENT IN CHRONIC RHEUMATOID ARTHRITIS WITH CHEST X-RAY AND SPIROMETRY IN A TEACHING HOSPITAL. JOURNAL OF EVOLUTION OF MEDICAL AND DENTAL SCIENCES-JEMDS. 2018;7(4):495-8.

**Reason for exclusion:** - HRCT not used

8. Aoust L, Berteloot L, Drabent P, Garcelon N, Bodemer C, Molina TJ, et al. Unclassifiable interstitial lung disease and autoimmunity: Towards IPAF in children? Pediatric Pulmonology. 2023;58(11):3303-13.

**Reason for exclusion:** - Prevalence not reported

9. Aubart F, Crestani B, Nicaise-Roland P, Tubach F, Bollet C, Dawidowicz K, et al. High levels of anti-cyclic citrullinated peptide autoantibodies are associated with co-occurrence of pulmonary diseases with rheumatoid arthritis. Journal of Rheumatology. 2011;38(6):979-82.

**Reason for exclusion:** - Background article

10. Avouac J, Cauvet A, Steelandt A, Shirai Y, Elhai M, Kuwana M, et al. Improving risk-stratification of rheumatoid arthritis patients for interstitial lung disease. PLoS One. 2020;15(5):e0232978.

**Reason for exclusion:** - Background article

11. Ayhan-Ardic FF, Oken O, Yorgancioglu ZR, Ustun N, Gokharman FD. Pulmonary involvement in lifelong non-smoking patients with rheumatoid arthritis and ankylosing spondylitis without respiratory symptoms. Clinical Rheumatology. 2006;25:213-8.

**Reason for exclusion:** - Background article

12. Bartels CM, Bell CL, Shinki K, Rosenthal A, Bridges AJ. Changing trends in serious extra-articular manifestations of rheumatoid arthritis among united state veterans over 20 years. Rheumatology. 2010;49(9):1670-5.

**Reason for exclusion:** - Case series/reviews

13. Bernstein EJ, Barr RG, Austin JHM, Kawut SM, Raghu G, Sell JL, et al. Rheumatoid arthritis-associated autoantibodies and subclinical interstitial lung disease: the Multi-Ethnic Study of Atherosclerosis. THORAX. 2016;71(12):1082-90.

**Reason for exclusion:** - HRCT not used

14. Bonfiglioli KR, Ribeiro ACdM, Carnieletto AP, Pereira I, Domiciano DS, Silva HCd, et al. Extra-articular manifestations of rheumatoid arthritis remain a major challenge: data from a large, multi-centric cohort. Advances in Rheumatology. 2023;63(1):34.

**Reason for exclusion:** - Prevalence not reported

15. Cassone G, Sebastiani M, Vacchi C, Cerri S, Salvarani C, Manfredi A. Pirfenidone for the treatment of interstitial lung disease associated to rheumatoid arthritis: a new scenario is coming? Respiratory Medicine Case Reports. 2020;30:101051.

**Reason for exclusion:** - Case series/reviews

16. Cavagna L, Meloni F, Meyer A, Sambataro G, Belliato M, De Langhe E, et al. Clinical spectrum time course in non-Asian patients positive for anti-MDA5 antibodies. Clinical and Experimental Rheumatology. 2022;40(2):274-83.

**Reason for exclusion:** - Case series/reviews

17. Cerro Chiang G, Lee C, Marchevsky A, Lewis MI. Severe Nonspecific Interstitial Pneumonia (NSIP) in an Adolescent. Case Reports in Pulmonology. 2022;2022:7757776.

**Reason for exclusion:** - Case series/reviews

18. Chai D, Sun D, Wang Y, Song Y, Wu N, Ye Q. Progression of radiographic fibrosis in rheumatoid arthritis-associated interstitial lung disease. Frontiers in Medicine. 2023;10:1265355.

**Reason for exclusion:** - Case series/reviews

19. Chandrashekara S, Shobha V, Dharmanand BG, Jois R, Kumar S, Mahendranath KM, et al. Reduced incidence of extra-articular manifestations of RA through effective disease control: Karnataka Rheumatoid Arthritis Comorbidity (KRAC) study. International Journal of Rheumatic Diseases. 2017;20(11):1694-703.

**Reason for exclusion:** - Prevalence not reported

20. Chen J, Chen Y, Liu D, Lin Y, Zhu L, Song S, et al. Predictors of long-term prognosis in rheumatoid arthritis-related interstitial lung disease. Scientific Reports. 2022;12(1).

**Reason for exclusion:** - Prevalence not reported

21. Chen J, Shi Y, Wang X, Huang H, Ascherman D. Asymptomatic Preclinical Rheumatoid Arthritis-Associated Interstitial Lung Disease. Clinical & Developmental Immunology. 2013;2013:406927.

**Reason for exclusion:** - Prevalence not reported

22. Chen RX, Zhao LD, Xiao XY, Song L, Du HY, Xu ZJ, et al. Distinctive Clinical Characteristics and Outcome of ILD-Onset Rheumatoid Arthritis and ACPA-Positive ILD: a Longitudinal Cohort of 282 Cases. Clinical Reviews in Allergy and Immunology. 2021;60(1):46-54.

**Reason for exclusion:** - Prevalence not reported

23. Cheng WC, Chang SH, Chen WC, Wu BR, Chen CH, Lin CC, et al. Application of impulse oscillometry to detect interstitial lung disease and airway disease in adults with rheumatoid arthritis. BMC Pulmonary Medicine. 2023;23(1).

**Reason for exclusion:** - Prevalence not reported

24. Chiu YH, Koops MFM, Voortman M, van Es HW, Langezaal LCM, Welsing PMJ, et al. Prognostication of progressive pulmonary fibrosis in connective tissue disease-associated interstitial lung diseases: A cohort study. Frontiers in Medicine. 2023;10:1106560.

**Reason for exclusion:** - Prevalence not reported

25. Cho S-K, Doyle TJ, Lee H, Jin Y, Tong AY, Ortiz AJS, et al. Validation of claims-based algorithms to identify interstitial lung disease in patients with rheumatoid arthritis. Seminars in arthritis and rheumatism. 2020;50(4):592-7.

**Reason for exclusion:** - Prevalence not reported

26. Chung JH, Cox CW, Montner SM, Adegunsoye A, Oldham JM, Husain AN, et al. CT features of the usual interstitial pneumonia pattern: Differentiating connective tissue disease-associated interstitial lung disease from idiopathic pulmonary fibrosis. American Journal of Roentgenology. 2018;210(2):307-13.

**Reason for exclusion:** - Case series/reviews

27. Chung JH, Montner SM, Thirkateh P, Cannon B, Barnett SD, Nathan SD. Computed Tomography Findings Suggestive of Connective Tissue Disease in the Setting of Usual Interstitial Pneumonia. Journal of Computer Assisted Tomography. 2021;45(5):776-81.

**Reason for exclusion:** - Case series/reviews

28. Cipriani NAMD, Strek MMD, Noth IMD, Gordon IOMDP, Charbeneau JMS, Krishnan JAMDP, et al. Pathologic Quantification of Connective Tissue Disease-Associated Versus Idiopathic Usual Interstitial Pneumonia. Archives of Pathology & Laboratory Medicine. 2012;136(10):1253-8.

**Reason for exclusion:** - Case series/reviews

29. Darrah E, Giles JT, Davis RL, Naik P, Wang H, Konig MF, et al. Autoantibodies to peptidylarginine deiminase 2 are associated with less severe disease in rheumatoid arthritis. Frontiers in Immunology. 2018;9:2696.

**Reason for exclusion:** - HRCT not used

30. Dawson J, Graham D, Desmond J, Fewins H, Lynch* M. Investigation of the chronic pulmonary effects of low‐dose oral methotrexate in patients with rheumatoid arthritis: a prospective study incorporating HRCT scanning and pulmonary function tests. Rheumatology. 2002;41(3):262-7.

**Reason for exclusion:** - background article

31. Dawson JK, Fewins HE, Desmond J, Lynch MP, Graham DR. Fibrosing alveolitis in patients with rheumatoid arthritis as assessed by high resolution computed tomography, chest radiography, and pulmonary function tests. Thorax. 2001;56(8):622-7.

**Reason for exclusion:** - HRCT not used

32. Dawson JK, Goodson NG, Graham DR, Lynch MP. Raised pulmonary artery pressures measured with Doppler echocardiography in rheumatoid arthritis patients. RHEUMATOLOGY. 2000;39(12):1320-5.

**Reason for exclusion:** - background article

33. de Souza SMP, Alves TSGN, Silva AF, de Oliveira IS, Laporte L, Nogueira C, et al. Lung involvement in patients with rheumatoid arthritis: a cross-sectional study. 2023.

**Reason for exclusion:** - background article

34. Detorakis EE, Magkanas E, Lasithiotaki I, Sidiropoulos P, Boumpas D, Gourtsoyiannis N, et al. Evolution of imaging findings, laboratory and functional parameters in rheumatoid arthritis patients after one year of treatment with anti-TNF-alpha agents. Clinical and Experimental Rheumatology. 2017;35(1):43-52.

**Reason for exclusion:** - Prevalence not reported

35. Dhooria S, Agarwal R, Sehgal IS, Prasad KT, Garg M, Bal A, et al. Spectrum of interstitial lung diseases at a tertiary center in a developing country: A study of 803 subjects. PloS one. 2018;13(2):e0191938.

**Reason for exclusion:** - Prevalence not reported

36. Di Carlo M, Tardella M, Filippucci E, Carotti M, Salaffi F. Lung ultrasound in patients with rheumatoid arthritis: definition of significant interstitial lung disease. Clinical and Experimental Rheumatology. 2022;40(3):495-500.

**Reason for exclusion:** - Prevalence not reported

37. Dias VL, Storrer KM. Prevalence of latent tuberculosis infection among patients with interstitial lung disease requiring immunosuppression. Jornal Brasileiro de Pneumologia. 2022;48(2):e20210382.

**Reason for exclusion:** - Wrong condition

38. Ding F, Yang L, Wang Y, Wang J, Ma Y, Jin J. Serum Rcn3 level is a potential diagnostic biomarker for connective tissue disease-associated interstitial lung disease and reflects the severity of pulmonary function. BMC Pulmonary Medicine. 2023;23(1):68.

**Reason for exclusion:** - Prevalence not reported

39. Doshi JA, Mundhra KS, Shah DS, Shah SN, Patel TV, Bhatt A. Role of High-Resolution CT Thorax in Diagnosing Interstitial Lung Disease and Its Association With Smoking and Connective Tissue Disorder. CUREUS JOURNAL OF MEDICAL SCIENCE. 2022;14(11).

**Reason for exclusion:** - Case series/reviews

40. Doyle J, Eliasson A, Argyros G, Dennis G, Finger D, Hurwitz K, Phillips Y. Prevalence of pulmonary disorders in patients with newly diagnosed rheumatoid arthritis. Clinical Rheumatology. 2000;19(3):217-21.

**Reason for exclusion:** - HRCT not used

41. Doyle TJ, Lee JS, Dellaripa PF, Lederer JA, Matteson EL, Fischer A, et al. A Roadmap to Promote Clinical and Translational Research in Rheumatoid Arthritis-Associated Interstitial Lung Disease A Dance Promote Clinical and Translational Research. CHEST. 2014;145(3):454-63.

**Reason for exclusion:** - Case series/reviews

42. Doyle TJ, Patel AS, Hatabu H, Nishino M, Wu GD, Osorio JC, et al. Detection of Rheumatoid Arthritis-Interstitial Lung Disease Is Enhanced by Serum Biomarkers. AMERICAN JOURNAL OF RESPIRATORY AND CRITICAL CARE MEDICINE. 2015;191(12):1403-12.

**Reason for exclusion:** - Background article

43. Duarte AC, Porter JC, Leandro MJ. The lung in a cohort of rheumatoid arthritis patients-an overview of different types of involvement and treatment. Rheumatology (United Kingdom). 2019;58(11):2031-8.

**Reason for exclusion:** - Unclear ILD case definition

44. Duchemann B, Annesi-Maesano I, de Naurois CJ, Sanyal S, Brillet P-Y, Brauner M, et al. Prevalence and incidence of interstitial lung diseases in a multi-ethnic county of Greater Paris. European Respiratory Journal. 2017;50(2).

**Reason for exclusion:** - Prevalence not reported

45. Elemary AMM, Elshawaf WM, Mahmoud Motawea S, Raafat HA, Metawie SA. Predictors of airway and parenchymal lung abnormalities in patients with rheumatoid arthritis. Egyptian Rheumatologist. 2021;43(2):125-30.

**Reason for exclusion:** - Prevalence not reported

46. Farquhar HJ, Beckert L, Edwards AL, Matteson EL, Frampton CMA, Ganly E, et al. Rheumatoid interstitial lung disease in Canterbury, Aotearoa New Zealand - A retrospective cohort study. Seminars in Arthritis and Rheumatism. 2024;64:152359.

**Reason for exclusion:** - Wong publication type

47. Fujita K, Ito Y, Hirai T, Kubo T, Togashi K, Ichiyama S, Mishima M. Prevalence and risk factors for chronic co-infection in pulmonary mycobacterium avium complex disease. BMJ Open Respiratory Research. 2014;1(1):e000050.

**Reason for exclusion:** - Wrong condition

48. Fuld JP, Johnson MK, Cotton MM, Carter R, et al. A longitudinal study of lung function in nonsmoking patients with rheumatoid arthritis*. Chest. 2003;124(4):1224-31.

**Reason for exclusion:** - HRCT not used

49. Furukawa H, Oka S, Shimada K, Sugii S, Ohashi J, Matsui T, et al. Association of Human Leukocyte Antigen with Interstitial Lung Disease in Rheumatoid Arthritis: A protective role for shared epitope. PLoS ONE. 2012;7(5).

**Reason for exclusion:** - Background article

50. Gaik OS, Jen DH, Hamid ZA, Aziz AA, Wong NI. Predictors and radiological characteristics of rheumatoid arthritis-associated interstitial lung disease in a multi-ethnic Malaysian cohort. Medical Journal of Malaysia. 2022;77(3):292-9.

**Reason for exclusion:** - Background article

51. Gbadamassi AG, Adjoh KS, Fianyo ANE, Adambounou TAS, Aziagbe AK, Efalou P. Rheumatoid arthritis-associated lung disease in black africans: Descriptive study of 28 cases in lomé. African Journal of Thoracic and Critical Care Medicine. 2020;26(4):129-32.

**Reason for exclusion:** - HRCT not used

52. Ge Y, Yang H, Jiang W, Tian X, Lu X, Wang G. Clinical characteristics of myositis patients with isolated anti-U1 ribonucleoprotein antibody resemble immune-mediated necrotizing myopathy. Therapeutic Advances in Musculoskeletal Disease. 2023;15.

**Reason for exclusion:** - Wrong condition

53. Gochuico BR, Avila NA, Chow CK, Novero LJ, Wu H-P, Ren P, et al. Progressive preclinical interstitial lung disease in rheumatoid arthritis. Archives of internal medicine. 2008;168(2):159-66.

**Reason for exclusion:** - Background article

54. Gouveia PA, Ferreira ECG, Neto PMC. Organizing Pneumonia Induced by Tocilizumab in a Patient with Rheumatoid Arthritis. CUREUS JOURNAL OF MEDICAL SCIENCE. 2020;12(2).

**Reason for exclusion:** - Case series/reviews

55. Hambly N, Farooqi MM, Dvorkin-Gheva A, Donohoe K, Garlick K, Scallan C, et al. Prevalence and characteristics of progressive fibrosing interstitial lung disease in a prospective registry. European Respiratory Journal. 2022;60(4).

**Reason for exclusion:** - Prevalence not reported

56. Hammoda RM, Moussa SG, Hassan RM. Prevalence of obesity in a cohort of egyptian rheumatoid arthritis patients and its implication on disease activity. Egyptian Journal of Hospital Medicine. 2021;82(3):536-41.

**Reason for exclusion:** - Prevalence not reported

57. Herrinton LJ, Harrold LR, Liu LY, Raebel MA, Taharka A, Winthrop KL, et al. Association between anti-TNF-α therapy and interstitial lung disease. PHARMACOEPIDEMIOLOGY AND DRUG SAFETY. 2013;22(4):394-402.

**Reason for exclusion:** - Unclear ILD case definition

58. Honne K, Bando M, Mieno MN, Iwamoto M, Minota S. Bronchiectasis is as crucial as interstitial lung disease in the severe pneumonia that occurs during treatment with biologic DMARDs in rheumatoid arthritis: a retrospective cohort study in a single facility. RHEUMATOLOGY INTERNATIONAL. 2022;42(8):1341-6.

**Reason for exclusion:** - Prevalence not reported

59. Huang SC, Doyle TJ, Hammer MM, Byrne SC, Huang WX, Marshall AA, et al. Rheumatoid arthritis-related lung disease detected on clinical chest computed tomography imaging: Prevalence, risk factors, and impact on mortality. SEMINARS IN ARTHRITIS AND RHEUMATISM. 2020;50(6):1216-25.

**Reason for exclusion:** - Prevalence not reported

60. Huang Y, Qiu Y, Xie Z, Zhang F, Zhang Y, Guan M, et al. Risk factors and prognosis of interstitial lung disease for primary Sjogren syndrome patients: A retrospective case-control study. Clinical rheumatology. 2023;42(11):3033-41.

**Reason for exclusion:** - Wrong condition

61. Jacob J, Song JW, Yoon H-Y, Cross G, Barnett J, Woo WL, et al. Prevalence and Effects of Emphysema in Never-Smokers with Rheumatoid Arthritis Interstitial Lung Disease. EBioMedicine. 2018;28:303-10.

**Reason for exclusion:** - Case series/reviews

62. Juge P-A, Borie R, Kannengiesser C, Gazal S, Revy P, Wemeau-Stervinou L, et al. Shared genetic predisposition in rheumatoid arthritis-interstitial lung disease and familial pulmonary fibrosis. European Respiratory Journal. 2017;49(5).

**Reason for exclusion:** - Prevalence not reported

63. Kakutani T, Hashimoto A, Tominaga A, Kodama K, Nogi S, Tsuno H, et al. Related factors, increased mortality and causes of death in patients with rheumatoid arthritis-associated interstitial lung disease. Modern Rheumatology. 2020;30(3):458-64.

**Reason for exclusion:** - Case series/reviews

64. Kang J, Seo WJ, Lee EY, Chang SH, Choe J, Hong S, Song JW. Pleuroparenchymal fibroelastosis in rheumatoid arthritis-associated interstitial lung disease. Respiratory Research. 2022;23(1):1-10.

**Reason for exclusion:** - Prevalence not reported

65. Kelly CA, Saravanan V, Nisar M, Arthanari S, Woodhead FA, Price-Forbes AN, et al. Rheumatoid arthritis-related interstitial lung disease: associations, prognostic factors and physiological and radiological characteristics-a large multicentre UK study. RHEUMATOLOGY. 2014;53(9):1676-82.

**Reason for exclusion:** - Prevalence not reported

66. Kim H, Cho SK, Song YJ, Kang J, Jeong SA, Kim HW, et al. Clinical characteristics of rheumatoid arthritis patients with interstitial lung disease: baseline data of a single-center prospective cohort. Arthritis Research and Therapy. 2023;25(1).

**Reason for exclusion:** - Prevalence not reported

67. Kreuter M, Herth FJ, Wacker M, Leidl R, Hellmann A, Pfeifer M, et al. Exploring clinical and epidemiological characteristics of interstitial lung diseases: rationale, aims, and design of a nationwide prospective registry—the EXCITING-ILD registry. BioMed research international. 2015;2015:123876.

**Reason for exclusion**: - Case series/reviews

68. Kur-Zalewska J, Kisiel B, Kania-Pudlo M, Tlustochowicz M, Chcialowski A, Tlustochowicz W. A dose-dependent beneficial effect of methotrexate on the risk of interstitial lung disease in rheumatoid arthritis patients. PLoS ONE. 2021;16(4):e0250339.

**Reason for exclusion:** - Case series/reviews

69. Kurata I, Tsuboi H, Terasaki M, Shimizu M, Toko H, Honda F, et al. Effect of Biological Disease-modifying Anti-rheumatic Drugs on Airway and Interstitial Lung Disease in Patients with Rheumatoid Arthritis. Internal medicine (Tokyo, Japan). 2019;58(12):1703-12.

**Reason for exclusion:** - Prevalence not reported

70. Kwon BS, Choe J, Chae EJ, Hwang HS, Kim YG, Song JW. Progressive fibrosing interstitial lung disease: prevalence and clinical outcome. Respiratory Research. 2021;22(1):282.

**Reason for exclusion:** - Case series/reviews

71. Lee JS, Lee EY, Ha YJ, Kang EH, Lee YJ, Song YW. Serum kl-6 levels reflect the severity of interstitial lung disease associated with connective tissue disease. Arthritis Research and Therapy. 2019;21(1):58.

**Reason for exclusion:** - Background article

72. Matsumoto T, Iwano S, Takahashi N, Asai S, Watanabe T, Asai N, et al. Association between chest computed tomography findings and respiratory adverse events in rheumatoid arthritis patients undergoing long-term biological therapy. International journal of rheumatic diseases. 2019;22(4):626-35.

**Reason for exclusion:** - Prevalence not reported

73. Matsuo T, Hashimoto M, Ito I, Kubo T, Uozumi R, Furu M, et al. Interleukin-18 is associated with the presence of interstitial lung disease in rheumatoid arthritis: a cross-sectional study. Scandinavian Journal of Rheumatology. 2019;48(2):87-94.

**Reason for exclusion:** - Background article

74. McDermott G, Gill R, Gagne S, Byrne S, Huang WX, Cui J, et al. Associations of the <i>MUC5B</i> Promoter Variant with Timing of Interstitial Lung Disease and Rheumatoid Arthritis Onset. RHEUMATOLOGY. 2022;61(12):4915-23.

**Reason for exclusion:** - Unclear ILD case definition

75. McFarlane IM, Zhaz SY, Bhamra MS, Burza A, Kolla S, Alvarez MR, et al. Assessment of interstitial lung disease among black rheumatoid arthritis patients. Clinical Rheumatology. 2019;38(12):3413-24.

**Reason for exclusion:** - Unclear ILD case definition

76. Md Yusof MY, Kabia A, Darby M, Lettieri G, Beirne P, Vital EM, et al. Effect of rituximab on the progression of rheumatoid arthritis-related interstitial lung disease: 10 years' experience at a single centre. Rheumatology (United Kingdom). 2017;56(8):1348-57.

**Reason for exclusion:** - Case series/reviews

77. Mirfeizi Z, Farrokh D, Javanbakht A, Raufi E. Chest high resolution computed tomography findings in connective tissue diseases. Tanaffos. 2013;12(3):49-52.

**Reason for exclusion:** - Case series/reviews

78. Moazedi-Fuerst F, Kielhauser S, Scheidl S, Tripolt N, Lutfi A, Yazdani-Biuki B, et al. Ultrasound screening for interstitial lung disease in rheumatoid arthritis. Clin Exp Rheumatol. 2014;32(2):199-203.

**Reason for exclusion:** - Case series/reviews

79. Moazedi-Fuerst FC, Kielhauser S, Brickmann K, Tripolt N, Meilinger M, Lutfi A, Graninger W. Sonographic assessment of interstitial lung disease in patients with rheumatoid arthritis, systemic sclerosis and systemic lupus erythematosus. Clinical and Experimental Rheumatology. 2015;33:87-91.

**Reason for exclusion: -** Background article

80. Mochizuki T, Yano K, Ikari K, Okazaki K. Radiological evaluation of interstitial lung disease in patients with rheumatoid arthritis treated with abatacept or JAK inhibitors for 1 year. Respiratory Investigation. 2023;61(3):359-63.

**Reason for exclusion:** - Prevalence not reported

81. Mohanty J, Singh M, Elluru SK. PULMONARY MANIFESTATIONS OF EARLY RHEUMATOID ARTHRITIS ON HRCT THORAX. JOURNAL OF EVOLUTION OF MEDICAL AND DENTAL SCIENCES-JEMDS. 2019;8(26):2097-100.

**Reason for exclusion:** - Prevalence not reported

82. Mohd Noor N, Mohd Shahrir MS, Shahid MS, Abdul Manap R, Shahizon Azura AM, Azhar Shah S. Clinical and high resolution computed tomography characteristics of patients with rheumatoid arthritis lung disease. International Journal of Rheumatic Diseases. 2009;12(2):136-44.

**Reason for exclusion:** **-** Prevalence not reported

83. Mori S, Cho I, Koga Y, Sugimoto M. Comparison of pulmonary abnormalities on high-resolution computed tomography in patients with early versus longstanding rheumatoid arthritis. Journal of Rheumatology. 2008;35(8):1513-21.

**Reason for exclusion:** - Case series/reviews

84. Mori S, Koga Y, Sugimoto M. Different risk factors between interstitial lung disease and airway disease in rheumatoid arthritis. Respiratory Medicine. 2012;106(11):1591-9.

**Reason for exclusion:** - Background article

85. Nashatyreva MS, Trofimenko IN, Chernyak BA, Avdeev SN. Pulmonary Fibrosis and Progressive Pulmonary Fibrosis in a Prospective Registry of Interstitial Lung Diseases in Eastern Siberia. Life. 2023;13(1).

**Reason for exclusion:** - Case series/reviews

86. Nasser M, Larrieu S, Boussel L, Si-Mohamed S, Bazin F, Marque S, et al. Estimates of epidemiology, mortality and disease burden associated with progressive fibrosing interstitial lung disease in France (the PROGRESS study). Respiratory Research. 2021;22(1):1-15.

**Reason for exclusion:** - Prevalence not reported

87. Nava-Quiroz KJ, Rojas-Serrano J, Perez-Rubio G, Buendia-Roldan I, Mejia M, Fernandez-Lopez JC, et al. Molecular Factors in PAD2 (PADI2) and PAD4 (PADI4) Are Associated with Interstitial Lung Disease Susceptibility in Rheumatoid Arthritis Patients. Cells. 2023;12(18):2235.

**Reason for exclusion**: - Prevalence not reported

88. Nurmi HM, Purokivi MK, Kärkkäinen MS, Kettunen HP, Selander TA, Kaarteenaho RL. Variable course of disease of rheumatoid arthritis-associated usual interstitial pneumonia compared to other subtypes. BMC Pulmonary Medicine. 2016;16(1):1.

**Reason for exclusion:** - Prevalence not reported

89. Okuda Y, Takasugi K, Imai A, Oyama T, Oyama H, Kawamura S. Clinical study of rheumatoid interstitial lung disease evaluated by high resolution computed tomography. Japanese Journal of Rheumatology. 1994;5(3):271-81.

**Reason for exclusion:** - Foreign language

90. Olson AL, Patnaik P, Hartmann N, Bohn RL, Garry EM, Wallace L. Prevalence and incidence of chronic fibrosing interstitial lung diseases with a progressive phenotype in the United States estimated in a large claims database analysis. Advances in Therapy. 2021;38(7):4100-14.

**Reason for exclusion:** - Prevalence not reported

91. Olson AL, Swigris JJ, Sprunger DB, Fischer A, Fernandez-Perez ER, Solomon J, et al. Rheumatoid Arthritis-Interstitial Lung Disease-associated Mortality. American Journal of Respiratory and Critical Care Medicine. 2011;183(3):372-8.

**Reason for exclusion:** - Prevalence not reported

92. Otsuka J, Yoshizawa S, Kudo K, Osoreda H, Ishimatsu A, Taguchi K, et al. Clinical features of acute exacerbation in rheumatoid arthritis–associated interstitial lung disease: Comparison with idiopathic pulmonary fibrosis. Respiratory Medicine. 2022;200.

**Reason for exclusion:** - Prevalence not reported

93. Oyama T, Kohno N, Yokoyama A, Hirasawa Y, Hiwada K, Oyama H, et al. Detection of Interstitial Pneumonitis in Patients with Rheumatoid Arthritis by Measuring Circulating Levels of KL-6, a Human MUC1 Mucin. Lung. 1997;175(6):379-85.

**Reason for exclusion:** - Background article

94. Paulin F, Doyle TJ, Fletcher EA, Ascherman DP, Rosas IO. Rheumatoid Arthritis-Associated Interstitial Lung Disease and Idiopathic Pulmonary Fibrosis: Shared Mechanistic and Phenotypic Traits Suggest Overlapping Disease Mechanisms. Revista de investigacion clinica; organo del Hospital de Enfermedades de la Nutricion. 2015;67(5):280-6.

**Reason for exclusion:** - Case series/reviews

95. Paulin F, Doyle TJ, Mercado JF, Fassola L, Fernandez M, Caro F, et al. Development of a risk indicator score for the identification of interstitial lung disease in patients with rheumatoid arthritis. Reumatologia Clinica. 2021;17(4):207-11.

**Reason for exclusion:** - Case series/reviews

96. Perez-Alvarez R, Perez-de-Lis M, Diaz-Lagares C, Pego-Reigosa JM, Retamozo S, Bove A, et al. Interstitial Lung Disease Induced or Exacerbated by TNF-Targeted Therapies: Analysis of 122 Cases. Seminars in Arthritis and Rheumatism. 2011;41(2):256-64.

**Reason for exclusion:** - Case series/reviews

97. Pierre‐Antoine J, Granger B, Marie‐Pierre D, Ebstein E, Fabienne LS, Kedra J, et al. A Risk Score to Detect Subclinical Rheumatoid Arthritis–Associated Interstitial Lung Disease. Arthritis & Rheumatology. 2022;74(11):1755-65.

**Reason for exclusion:** - Prevalence not reported

98. Raniga S, Sharma P, Kaur G, Arora A, Khalasi Y, Vohra P, Shah DS. "Interstitial Lung Disease (Ild) in Rheumatoid Arthritis (Ra)" - A study of thirty cases. Indian Journal of Radiology and Imaging. 2006;16(4):835-9.

**Reason for exclusion:** - Prevalence not reported

99. Reiseter S, Gunnarsson R, Mogens Aaløkken T, Lund MB, Mynarek G, Corander J, et al. Progression and mortality of interstitial lung disease in mixed connective tissue disease: a long-term observational nationwide cohort study. Rheumatology. 2018;57(2):255-62.

**Reason for exclusion:** - Wrong condition

100. Robles-Pérez A, Luburich P, Bolivar S, Dorca J, Nolla J, Molina-Molina M, Narváez J. A prospective study of lung disease in a cohort of early rheumatoid arthritis patients. Scientific reports. 2020;10(1):15640.

**Reason for exclusion:** - Prevalence not reported

101. Robles-Perez A, Luburich P, Rodriguez-Sanchon B, Dorca J, Nolla JM, Molina-Molina M, Narvaez-Garcia J. Preclinical lung disease in early rheumatoid arthritis. Chronic Respiratory Disease. 2016;13(1):75-81.

**Reason for exclusion:** - Background article

102. Rosas IO, Yao J, Avila NA, Chow CK, Gahl WA, Gochuico BR, et al. Automated quantification of high-resolution CT scan findings in individuals at risk for pulmonary fibrosis. CHEST. 2011;140(6):1590-7.

**Reason for exclusion:** - Prevalence not reported

103. Saadati N, Naghibzadeh B, Khalilipour A, Miri M. Association between rheumatoid arthritis and pulmonary hypertension: A clinical investigation. Acta Medica Iranica. 2020;58(11):567-71.

**Reason for exclusion:** - Wrong condition

104. Saag KG, Kolluri S, Koehnke RK, Georgou TA, Rachow JW, Hunninghake GW, Schwartz DA. Rheumatoid arthritis lung disease - Determinants of radiographic and physiologic abnormalities. ARTHRITIS AND RHEUMATISM. 1996;39(10):1711-9.

**Reason for exclusion:** - HRCT not used

105. Santos-Moreno P, Linares-Contreras MF, Rodríguez-Vargas GS, Rodríguez-Linares P, Mata-Hurtado A, Ibatá L, et al. Usefulness of Lung Ultrasound as a Method for Early Diagnosis of Interstitial Lung Disease in Patients with Rheumatoid Arthritis. Open Access Rheumatology: Research and Reviews. 2024;16:9-20.

**Reason for exclusion:** - Prevalence not reported

106. Sato M, Tabata E, Takemura T, Okuda R, Komatsu S, Okudela K, et al. A Retrospective Study of the Clinical, Radiological, and Pathological Characteristics of Patients with Interstitial Pneumonia Preceding Rheumatoid Arthritis. Internal Medicine. 2023;62(12):1723-31.

**Reason for exclusion:** - Prevalence not reported

107. Sebastiani M, Venerito V, Laurino E, Gentileschi S, Atzeni F, Canofari C, et al. Fibrosing Progressive Interstitial Lung Disease in Rheumatoid Arthritis: A Multicentre Italian Study. Journal of Clinical Medicine. 2023;12(22):7041.

**Reason for exclusion:** - Case series/reviews

108. Shen G, Yang S, Yao K, Shi W, Wang Q, Haxhiu E, Dong L. Clinical characteristics and serum levels of tumor markers of connective tissue disease-associated interstitial lung disease. International Journal of Clinical and Experimental Medicine. 2019;12(5):5497-506.

**Reason for exclusion:** - Prevalence not reported

109. Shimizu T, Nagafuchi Y, Harada H, Tsuchida Y, Tsuchiya H, Hanata N, et al. Decreased peripheral blood memory B cells are associated with the presence of interstitial lung disease in rheumatoid arthritis: a case-control study. Modern Rheumatology. 2021;31(1):127-32.

**Reason for exclusion**: - Prevalence not reported

110. Singh R, Krishnamurthy P, Deepak D, Sharma B, Prasad A. Small airway disease and its predictors in patients with rheumatoid arthritis. RESPIRATORY INVESTIGATION. 2022;60(3):379-84.

**Reason for exclusion**: - Wrong condition

111. Solomon JJ, Danoff SK, Goldberg HJ, Woodhead F, Kolb M, Chambers DC, et al. The Design and Rationale of the Trail1 Trial: A Randomized Double-Blind Phase 2 Clinical Trial of Pirfenidone in Rheumatoid Arthritis-Associated Interstitial Lung Disease. ADVANCES IN THERAPY. 2019;36(11):3279-87.

**Reason for exclusion**: - Case series/reviews

112. Sugano E, Tanaka E, Inoue E, Honda S, Abe M, Saka K, et al. Impact of interstitial lung disease on clinical remission and unfavourable events of rheumatoid arthritis: results from the IORRA cohort. Rheumatology. 2023:kead317.

**Reason for exclusion:** - Background article

113. Tanaka A, Kurasawa K, Soda S, Takamura Y, Miyao T, Hasegawa A, et al. Changing patterns of pulmonary abnormalities in rheumatoid arthritis. Respiratory Investigation. 2023;61(1):27-39.

**Reason for exclusion:** - Background article

114. Tekeli AH. Pulmonary and cardiac involvement in patients with rheumatoid arthritis and ankylosing spondylitis. European Research Journal. 2023;9(2):291-300.

**Reason for exclusion:** - Prevalence not reported

115. Wada Y, Kuroda T, Murasawa A, Nakano M, Narita I. Anti-neutrophil cytoplasmic autoantibodies against bactericidal/ permeability-increasing protein in patients with rheumatoid arthritis and their correlation with bronchial involvement. Modern Rheumatology. 2010;20(3):252-6.

**Reason for exclusion:** **-** Background article

116. Wang T, Zheng X-J, Liang B-M, Liang Z-A. Clinical features of rheumatoid arthritis-associated interstitial lung disease. Scientific Reports. 2015;5(1):14897.

**Reason for exclusion**: - Background article

117. Wilson TM, Solomon JJ, Humphries SM, Swigris JJ, Ahmed F, Wang H, et al. Serum antibodies to peptidylarginine deiminase-4 in rheumatoid arthritis associated-interstitial lung disease are associated with decreased lung fibrosis and improved survival. American Journal of the Medical Sciences. 2023;365(6):480-7.

**Reason for exclusion:** - Case series/reviews

118. Yin Y, Liang D, Zhao L, Yang L, Liu W, Ren Y, et al. Anti-Cyclic Citrullinated Peptide Antibody Is Associated with Interstitial Lung Disease in Patients with Rheumatoid Arthritis. PLoS One. 2014;9(4).

**Reason for exclusion:** - Background article

119. Youssef AA, Machaly SA, El-Dosoky ME, El-Maghraby NM. Respiratory symptoms in rheumatoid arthritis: Relation to pulmonary abnormalities detected by high-resolution CT and pulmonary functional testing. Rheumatology International. 2012;32(7):1985-95.

**Reason for exclusion:** - Case series/reviews

120. Zamora‐Legoff JA, Krause ML, Crowson CS, Ryu JH, Matteson EL. Progressive Decline of Lung Function in Rheumatoid Arthritis-Associated Interstitial Lung Disease. Arthritis & Rheumatology. 2017;69(3):542-9.

**Reason for exclusion:** - Prevalence not reported

121. Zhang YF, Li HB, Wu NW, Dong X, Zheng Y. Retrospective study of the clinical characteristics and risk factors of rheumatoid arthritis-associated interstitial lung disease. CLINICAL RHEUMATOLOGY. 2017;36(4):817-23.

**Reason for exclusion:** - Prevalence not reported

122. Ziai WC, Rehman M. Infection or inflammation and ICU seizures. Seizures in Critical Care: A Guide to Diagnosis and Therapeutics. 2010:233-161.

**Reason for exclusion:** - Case series/reviews

123. Zrour SH, Touzi M, Bejia I, Golli M, Rouatbi N, Sakly N, et al. Correlations between high-resolution computed tomography of the chest and clinical function in patients with rheumatoid arthritis - Prospective study in 75 patients. JOINT BONE SPINE. 2005;72(1):41-7.

**Reason for exclusion:** - Case series/reviews

124. Zubairi ABS, Ahmad H, Hassan M, Siddiqui F, Iqbal N, Riaz M, Irfan M. Comparative analysis of connective tissue disease-Associated interstitial lung disease and idiopathic pulmonary fibrosis from a tertiary care centre in Pakistan. Journal of the Pakistan Medical Association. 2021;71(10):2330-4.

**Reason for exclusion:** - Prevalence not reported
