# Supplementary material for: The global prevalence of interstitial lung disease in patients with rheumatoid arthritis: a systematic review and meta-analysis
Source: Rheumatol Int. 2025 Jan 18;45(2):34. doi: 10.1007/s00296-025-05789-4 (PMC11742767; doi:10.1007/s00296-025-05789-4)
Supplement: Supplementary file 9 — Supplementary Material 5 [file 296_2025_5789_MOESM9_ESM.docx]

The global prevalence of interstitial lung disease in patients with rheumatoid arthritis: A systematic review and meta-analysis

Hari Prasanna ^1*^, Charles A Inderjeeth ^1,3^ Johannes C Nossent^1,3^, Khalid B Almutairi1 ^1,2^

**Affiliations**

1 School of Medicine, The University of Western Australia, Perth, Western Australia, Australia

2 Pharmacy Department, King Fahd Specialist Hospital, Burydah, Al Qassim, Saudi Arabia

3 Geronto-Rheumatology, Sir Charles Gairdner and Osborne Park Health Care Group, Perth, Western Australia, Australia

* First and corresponding author: Mr Hari Prasanna

* Corresponding author E-mail: [22981086@student.uwa.edu.au](mailto:22981086@student.uwa.edu.au)

**Address:**

Mr Hari Prasanna

School of Medicine

University of Western Australia

35 Stirling Highway

Perth WA 6009 Australia

**Appendix 9**

***Table 14: Prevalence of RA-ILD when pooled across different subgroups***

| Subgroup | Number of cohorts | The pooled prevalence proportion [95% CI] | I2 (%) | Cochran's Q | Test for subgroup difference (p-value) |
| --- | --- | --- | --- | --- | --- |
| A) Geography |  |  |  |  | |
| Asia | 16 | 0.2401 [0.1756; 0.3192] | 95.3 | 321.6 | 0.256 |
| Europe | 6 | 0.1015 [0.0286; 0.3023] | 98.6 | 347.74 |  |
| North America | 5 | 0.1800 [0.0415; 0.5267] | 98.5 | 261.86 |  |
| Africa | 4 | 0.3815 [0.0229; 0.9420] | 96 | 75.16 |  |
| South America | 3 | 0.2464 [0.0218; 0.8278] | 89.4 | 18.84 |  |
| Oceania | 1 | 0.3333 [0.2000; 0.5000] | -- | -- |  |
| B) Classification Criteria | | | | | |
| 2010 ACR/EULAR | 20 | 0.2560 [0.1624; 0.3792] | 97.9 | 889.34 | 0.215 |
| 1987 ARA | 21 | 0.1843 [0.1220; 0.2686] | 98.6 | 1438.47 |  |
| C) Risk of Bias | | | | | |
| Low | 30 | 0.2041 [0.1391; 0.2892] | 98 | 1471.19 | 0.2546 |
| Moderate | 5 | 0.2756 [0.1551; 0.4409] | 95.8 | 95.58 |  |
| D) Sampling Methodology | | | | | |
| Sampled population studies | 19 | 0.2354 [0.1377; 0.3724] | 97.6 | 744.56 | 0.4785 |
| Population database studies | 16 | 0.1895 [0.1250; 0.2767] | 98.4 | 942.79 |  |
| E) Socioeconomic Status | | | | | |
| High Income Countries | 16 | 0.1333 [0.0784; 0.2174] | 97.7 | 661.84 | 0.0042 |
| Upper Middle Income Countries | 13 | 0.3041 [0.2360; 0.3820] | 91.7 | 144.39 |  |
| Lower Middle Income Countries | 6 | 0.3299 [0.0792; 0.7381] | 96.6 | 146.51 |  |
| F) Time period | | | | | |
| Before June 2014 | 13 | 0.1919 [0.1106; 0.3121] | 97.9 | 558.47 | 0.945 |
| After June 2014 | 9 | 0.2404 [0.1141; 0.4374] | 90.6 | 84.91 |  |
| Spans across both time periods | 6 | 0.2093 [0.0746; 0.4648] | 99.2 | 613.58 |  |
