# Supplementary material for: The global prevalence of interstitial lung disease in patients with rheumatoid arthritis: a systematic review and meta-analysis
Source: Rheumatol Int. 2025 Jan 18;45(2):34. doi: 10.1007/s00296-025-05789-4 (PMC11742767; doi:10.1007/s00296-025-05789-4)

The global prevalence of interstitial lung disease in patients with rheumatoid arthritis: A systematic review and meta-analysis

Hari Prasanna ^1*^, Charles A Inderjeeth ^1,3^ Johannes C Nossent^1,3^, Khalid B Almutairi1 ^1,2^

**Affiliations**

1 School of Medicine, The University of Western Australia, Perth, Western Australia, Australia

2 Pharmacy Department, King Fahd Specialist Hospital, Burydah, Al Qassim, Saudi Arabia

3 Geronto-Rheumatology, Sir Charles Gairdner and Osborne Park Health Care Group, Perth, Western Australia, Australia

* First and corresponding author: Mr Hari Prasanna

* Corresponding author E-mail: [22981086@student.uwa.edu.au](mailto:22981086@student.uwa.edu.au)

**Address:**

Mr Hari Prasanna

School of Medicine

University of Western Australia

35 Stirling Highway

Perth WA 6009 Australia

**Appendix 10**

***Multivariate meta-regression results***


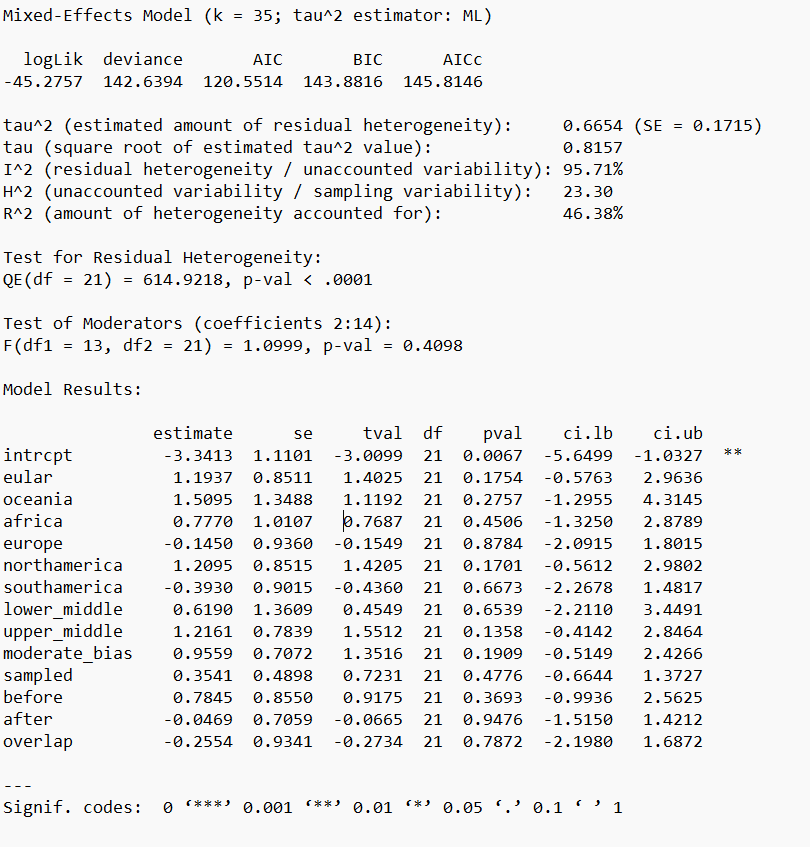


***Univariate meta-regression results***

1. Risk of bias:-


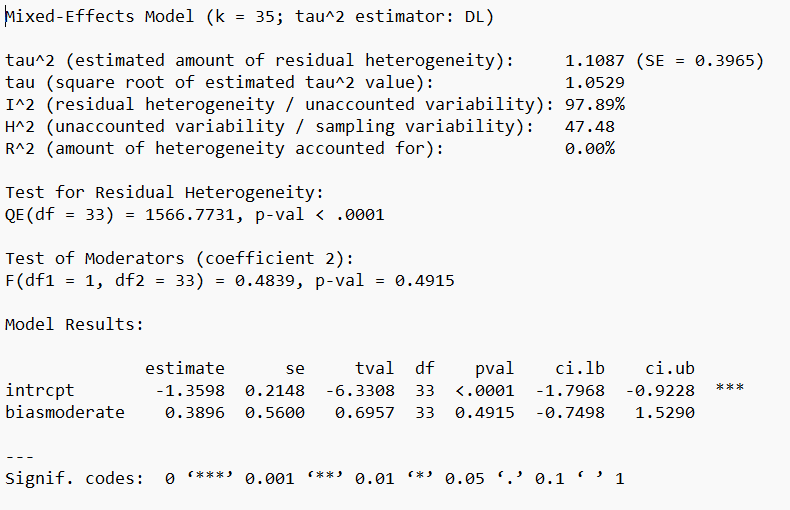


1. Geography :-


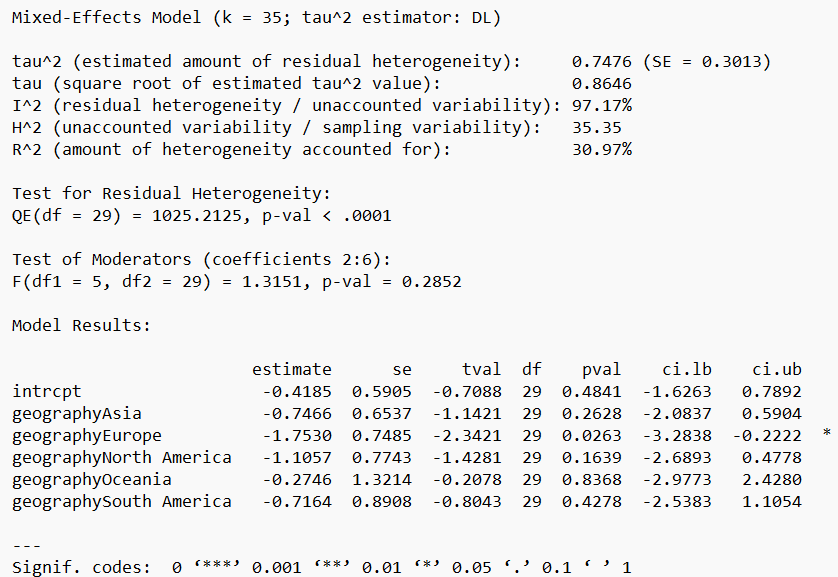


1. Socioeconomic status :-


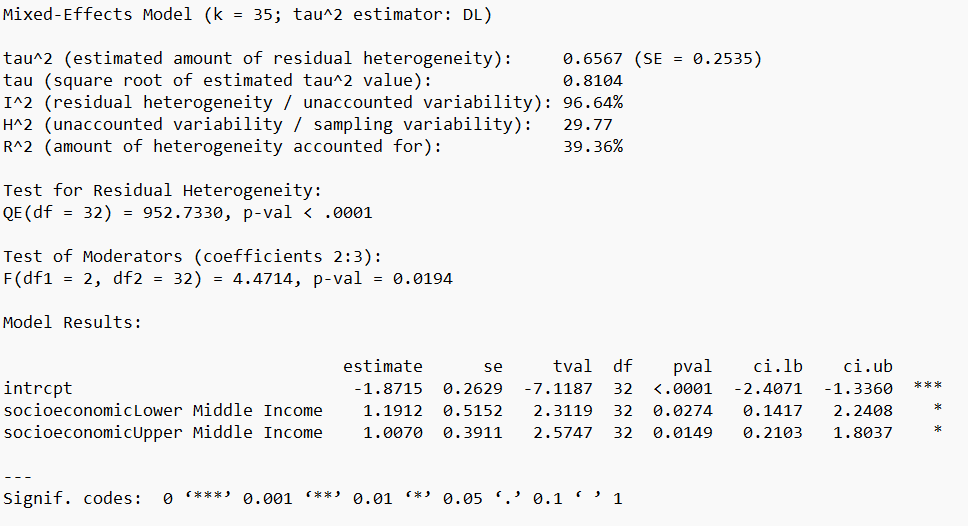


1. Sampling methodology


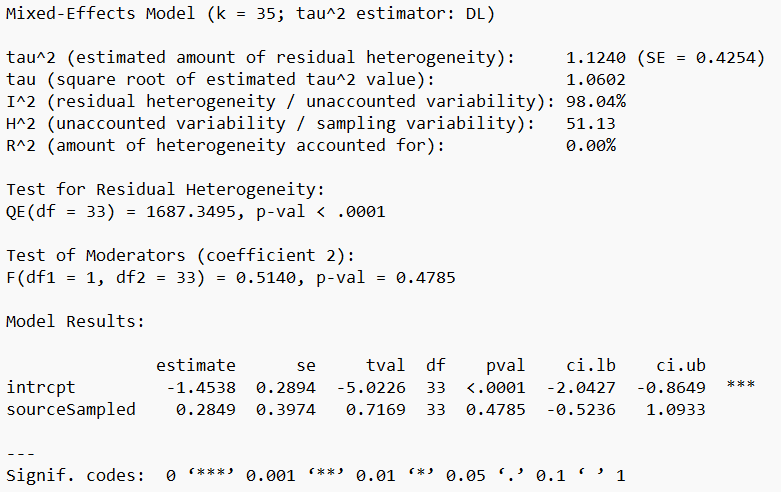


1. Time period of recruitment :-


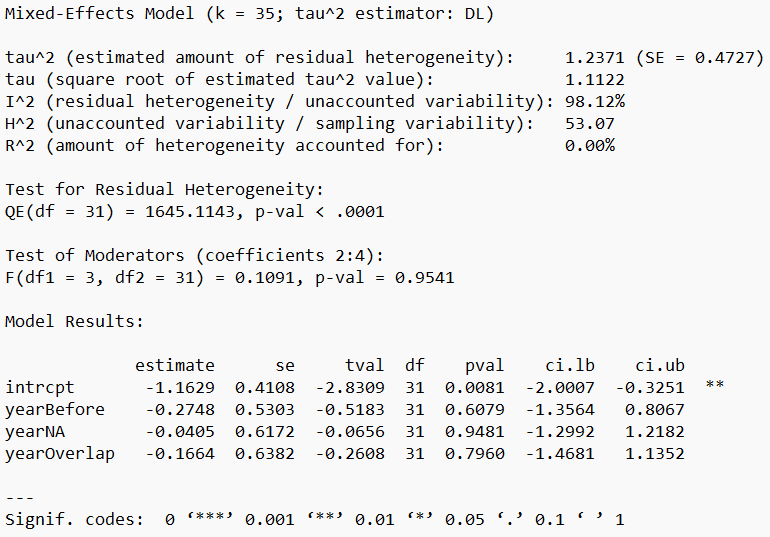


1. RA Classification criteria:-


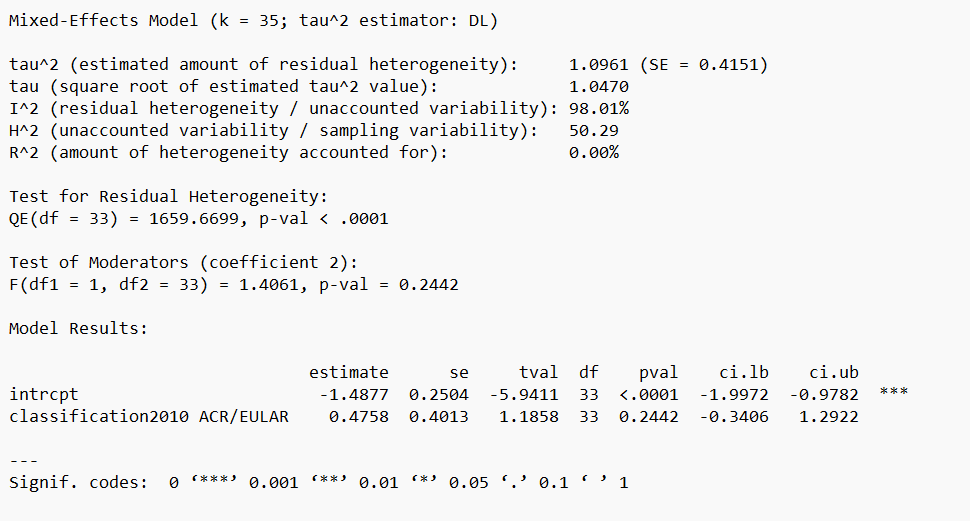

Supplement: Supplementary file 10 — Supplementary Material 6 [file 296_2025_5789_MOESM10_ESM.docx]
