# Supplementary material for: The global prevalence of interstitial lung disease in patients with rheumatoid arthritis: a systematic review and meta-analysis
Source: Rheumatol Int. 2025 Jan 18;45(2):34. doi: 10.1007/s00296-025-05789-4 (PMC11742767; doi:10.1007/s00296-025-05789-4)
Supplement: Supplementary file 11 — Supplementary Material 7 [file 296_2025_5789_MOESM11_ESM.docx]

The global prevalence of interstitial lung disease in patients with rheumatoid arthritis: A systematic review and meta-analysis

Hari Prasanna ^1*^, Charles A Inderjeeth ^1,3^ Johannes C Nossent^1,3^, Khalid B Almutairi1 ^1,2^

**Affiliations**

1 School of Medicine, The University of Western Australia, Perth, Western Australia, Australia

2 Pharmacy Department, King Fahd Specialist Hospital, Burydah, Al Qassim, Saudi Arabia

3 Geronto-Rheumatology, Sir Charles Gairdner and Osborne Park Health Care Group, Perth, Western Australia, Australia

* First and corresponding author: Mr Hari Prasanna

* Corresponding author E-mail: [22981086@student.uwa.edu.au](mailto:22981086@student.uwa.edu.au)

**Address:**

Mr Hari Prasanna

School of Medicine

University of Western Australia

35 Stirling Highway

Perth WA 6009 Australia

**Appendix 11**

**Pearson correlation analyses**

1. Smoking


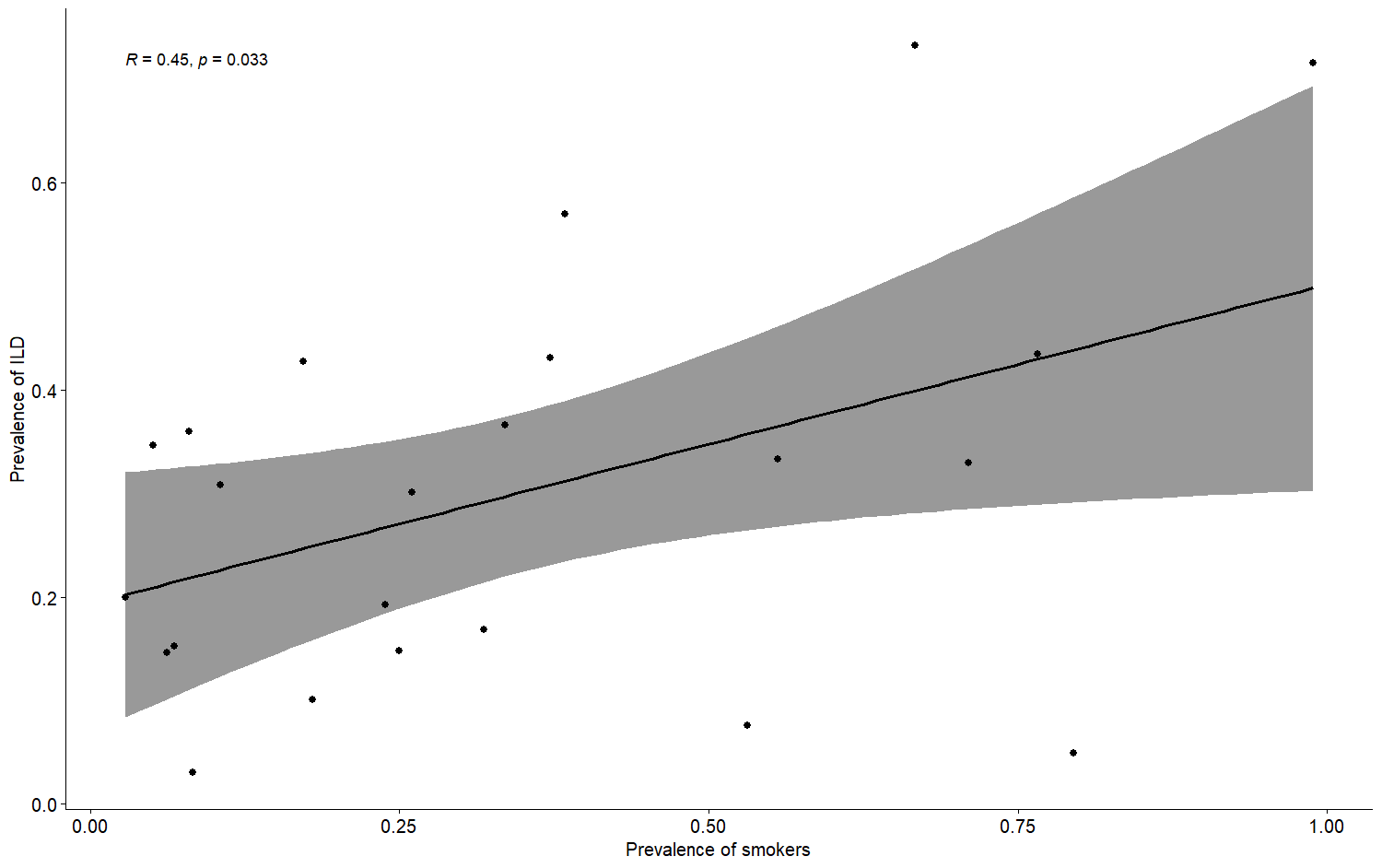


1. Methotrexate use


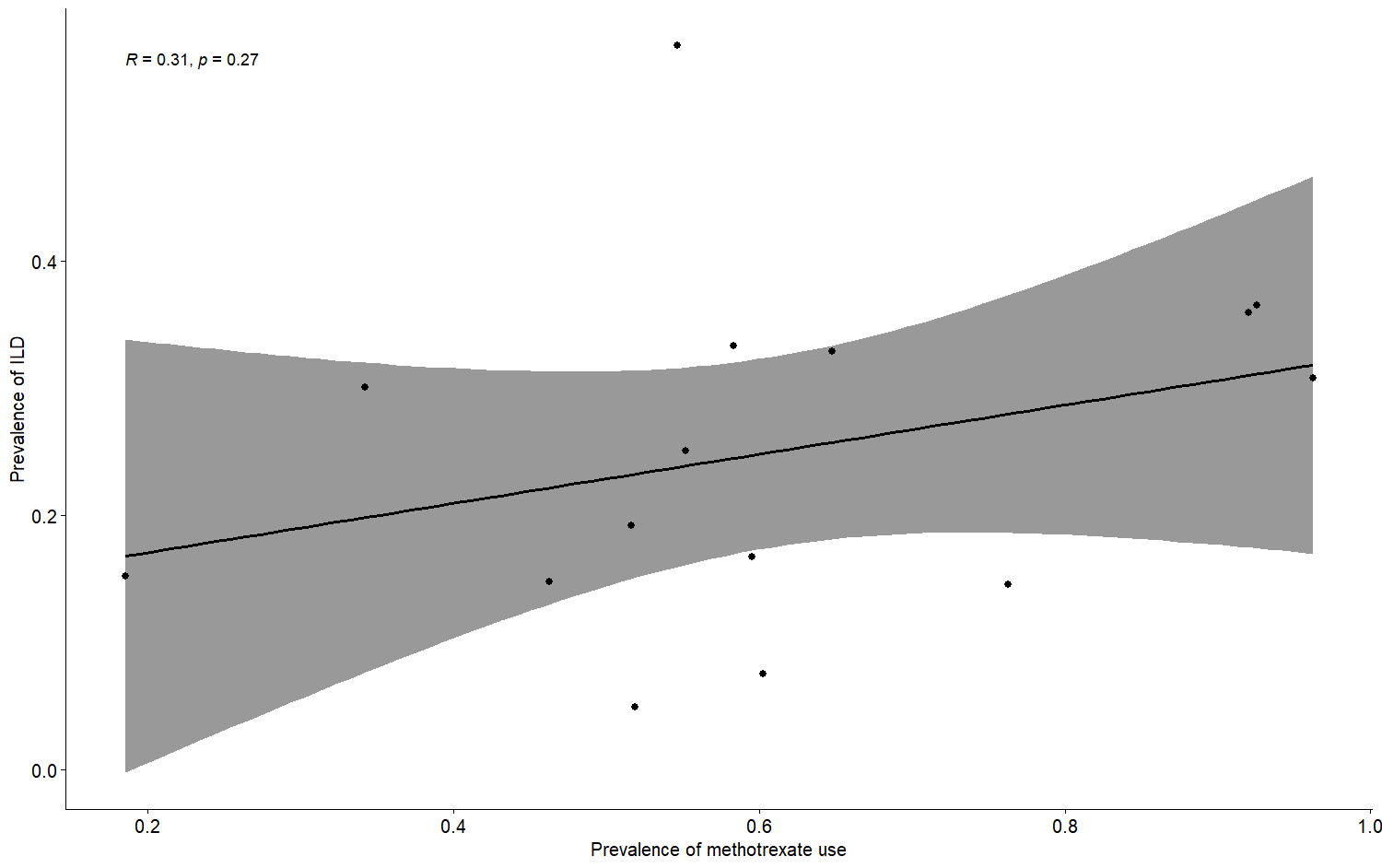


1. Leflunomide use


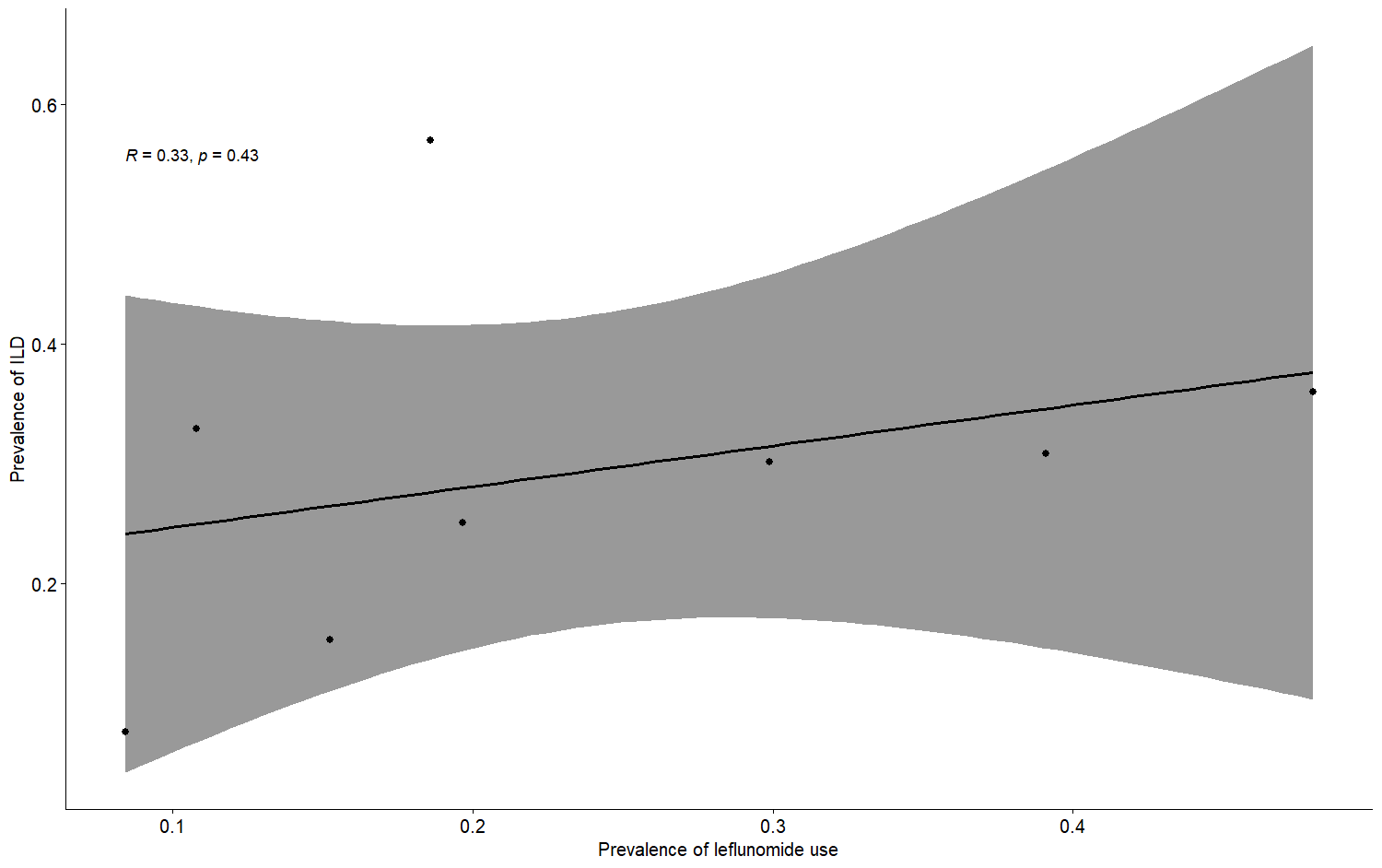


1. Mean age


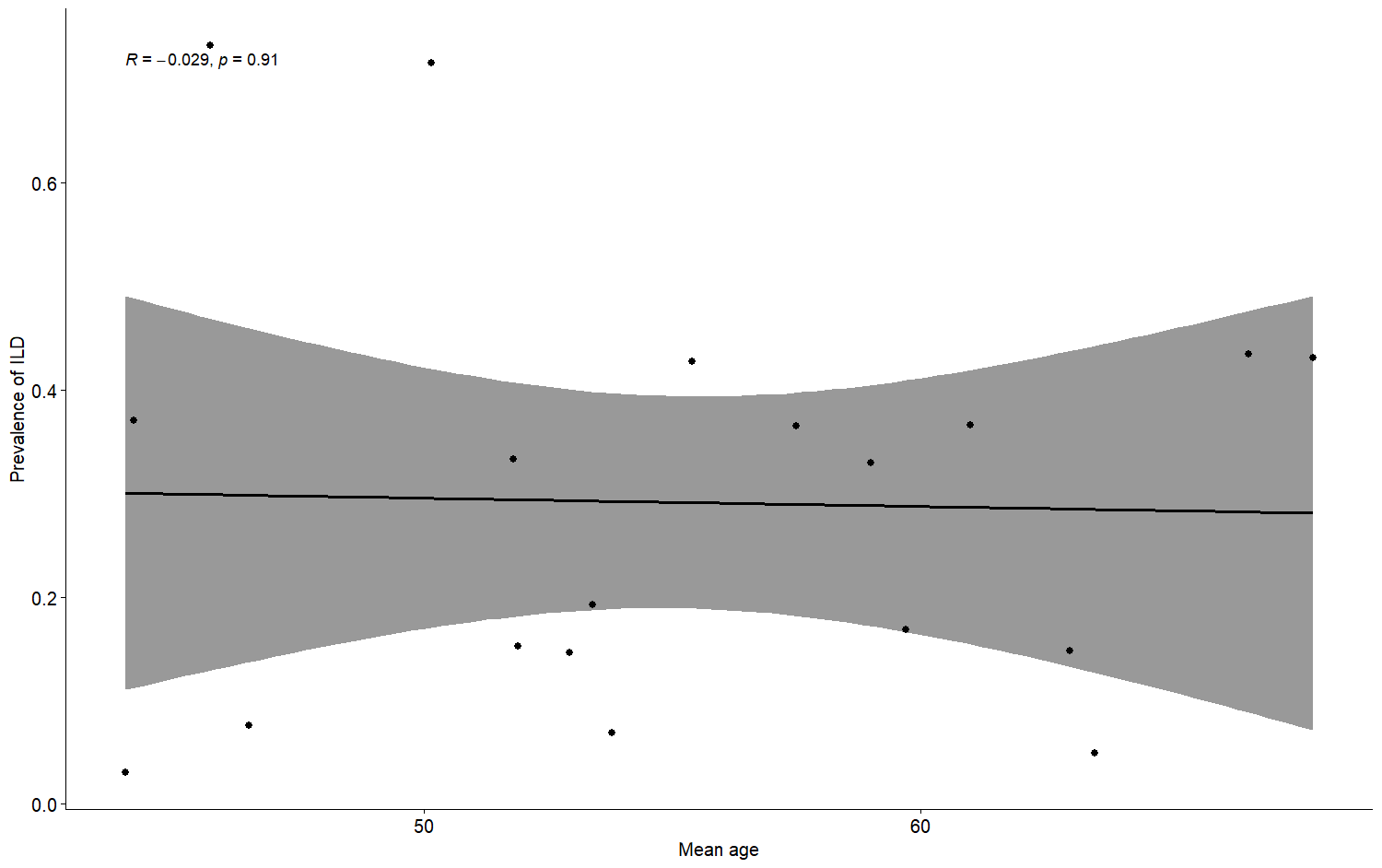


1. Prevalence of male participants


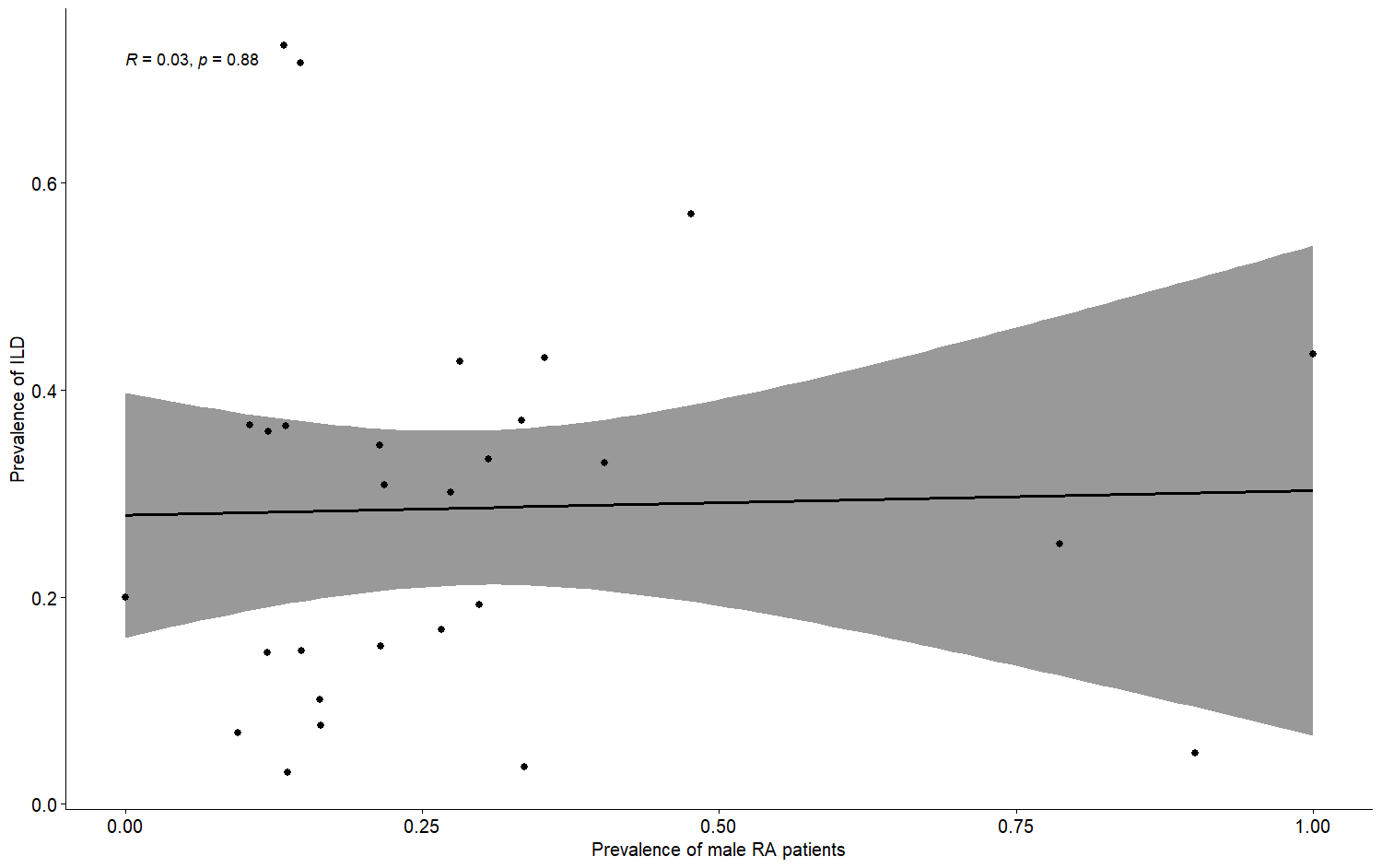


1. RA disease duration


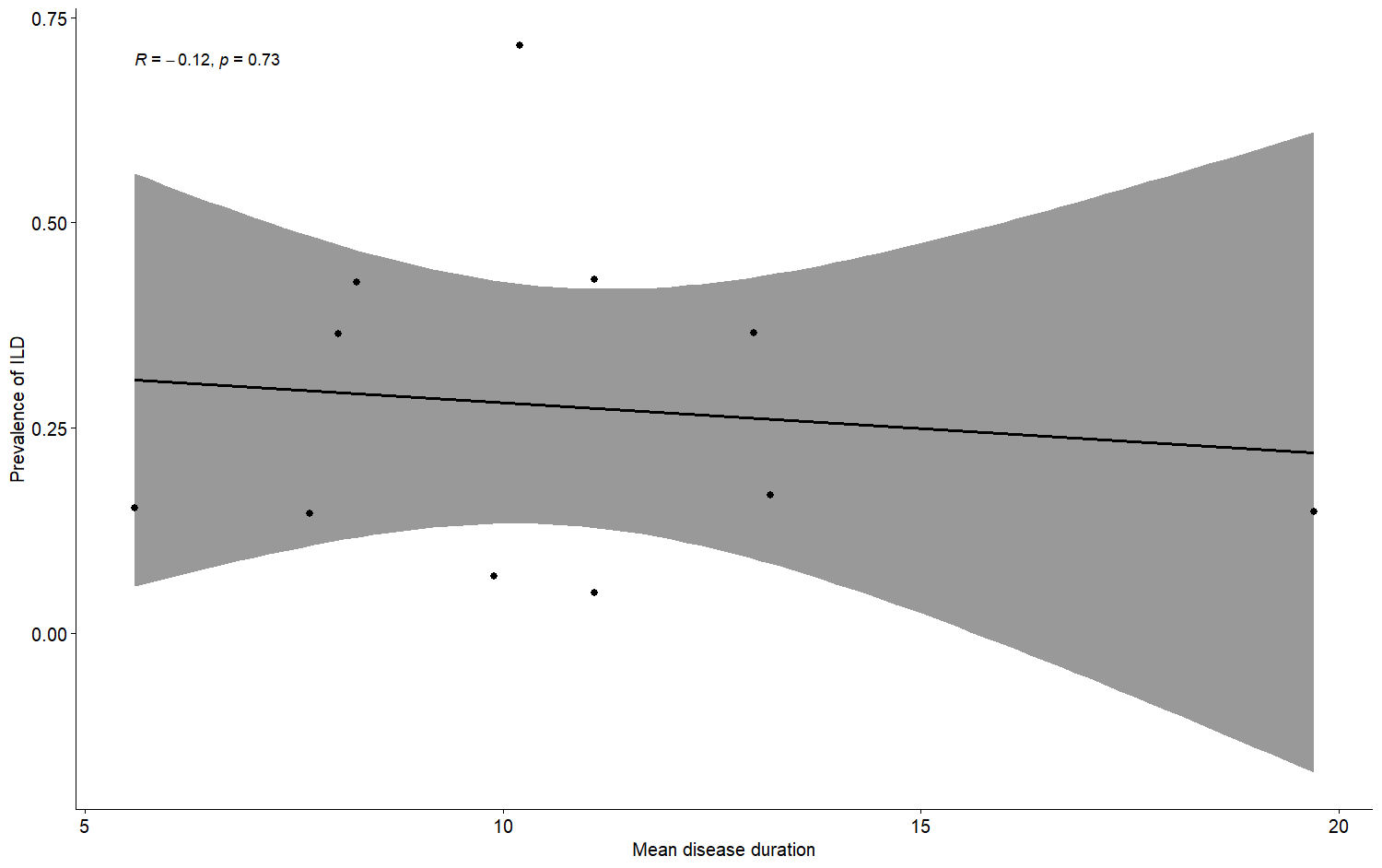


**Spearman’s rank correlation analyses**

1. Smoking


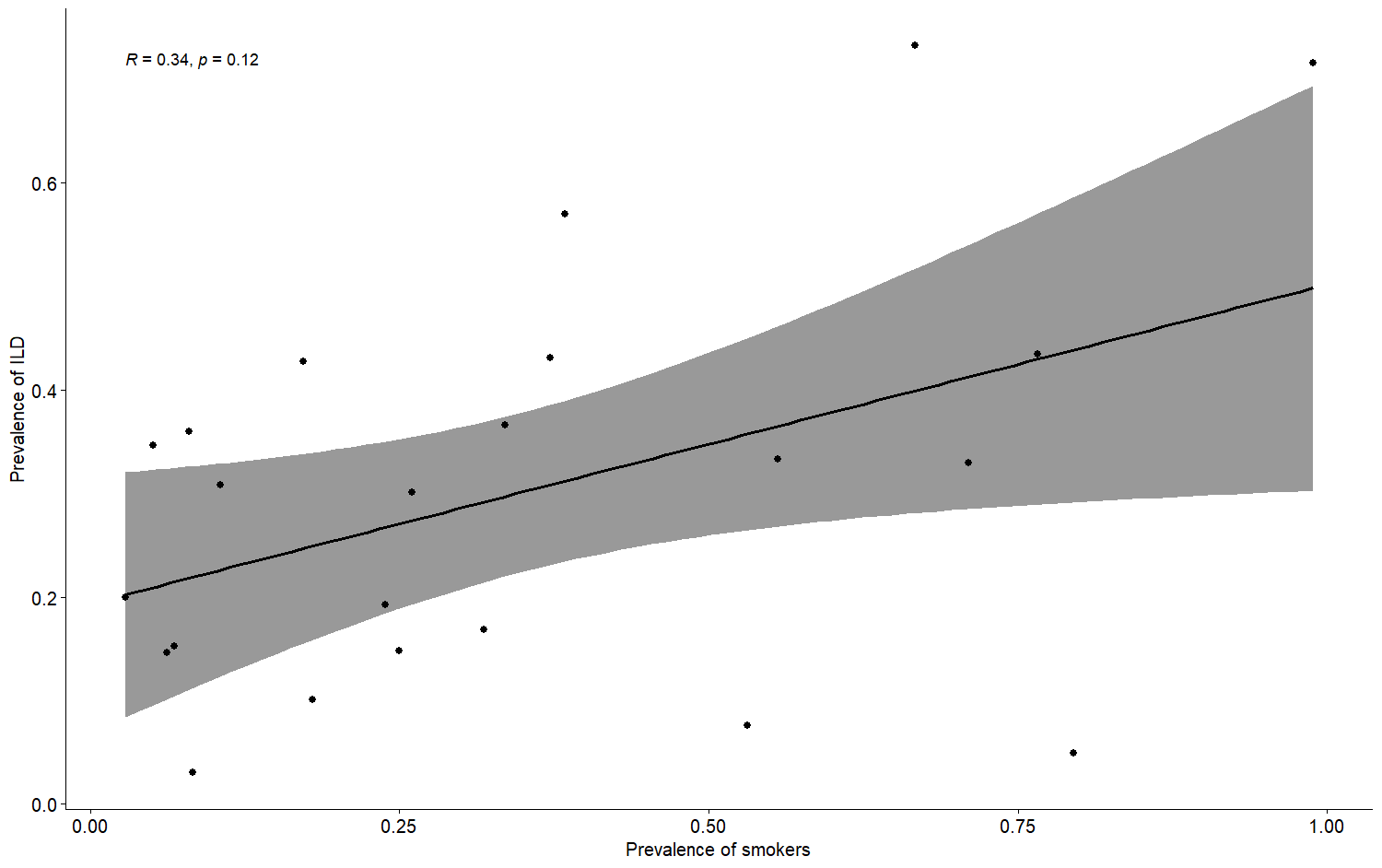


1. Methotrexate use


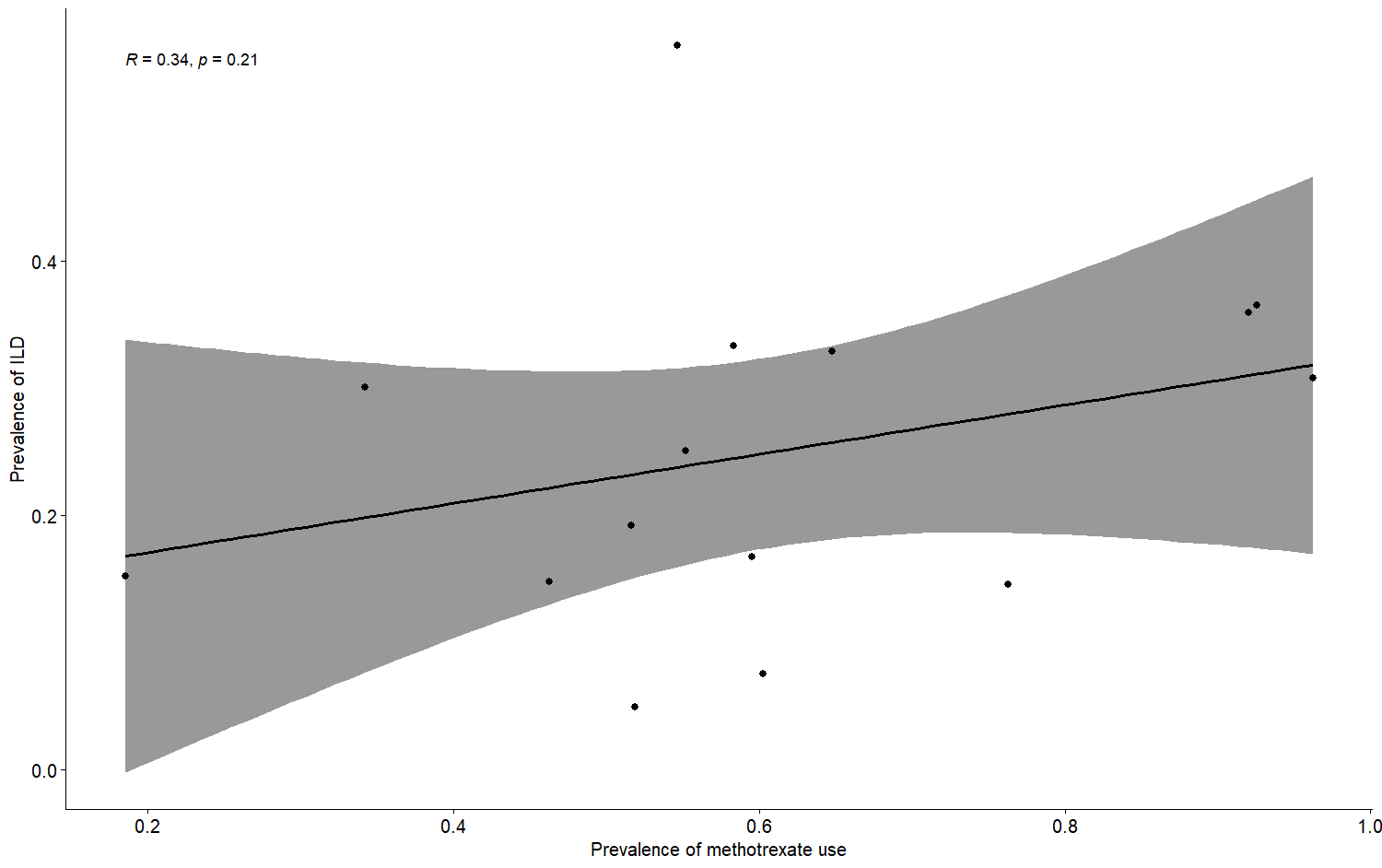


1. Leflunomide use


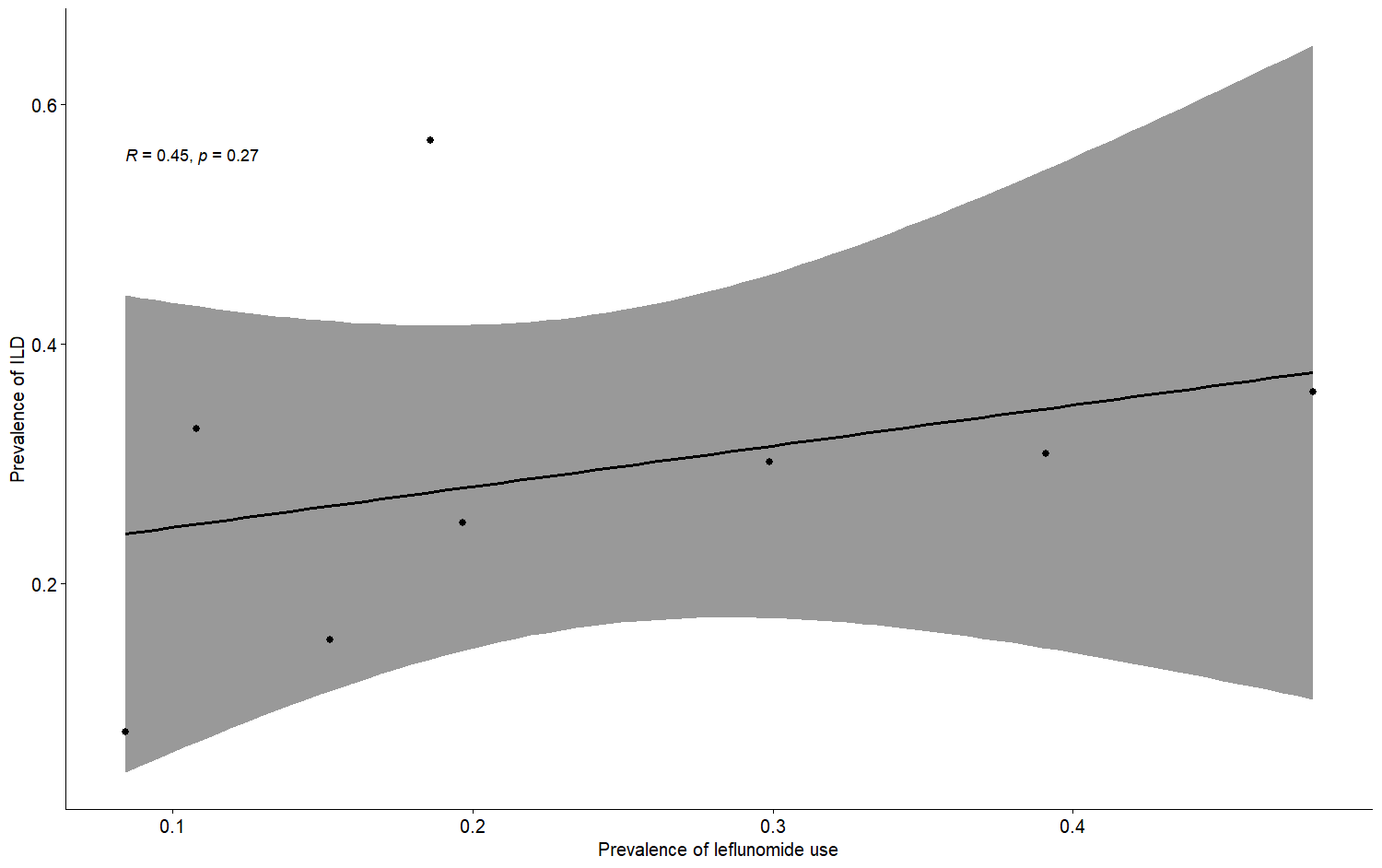


1. Mean age


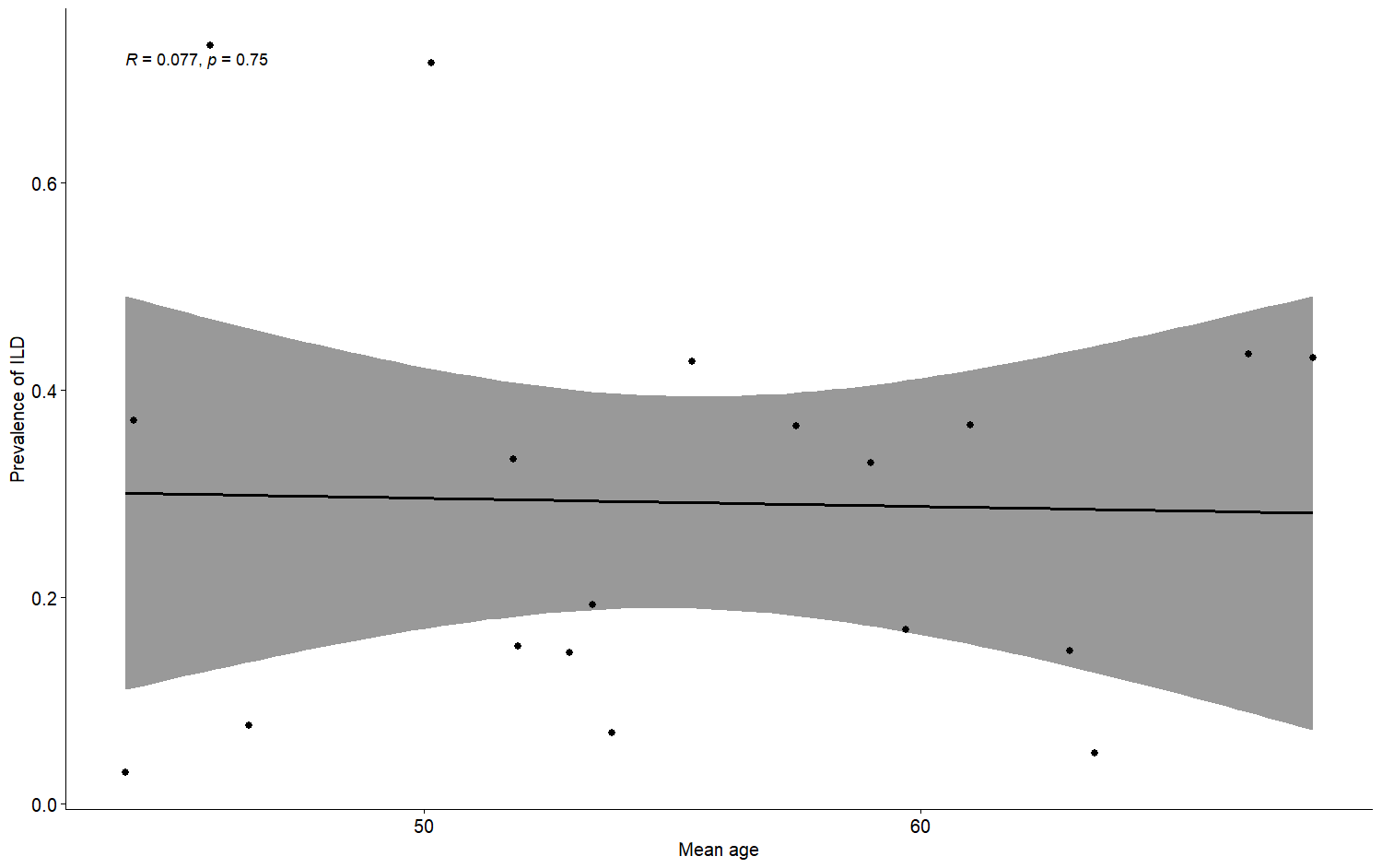


1. Prevalence of male participants


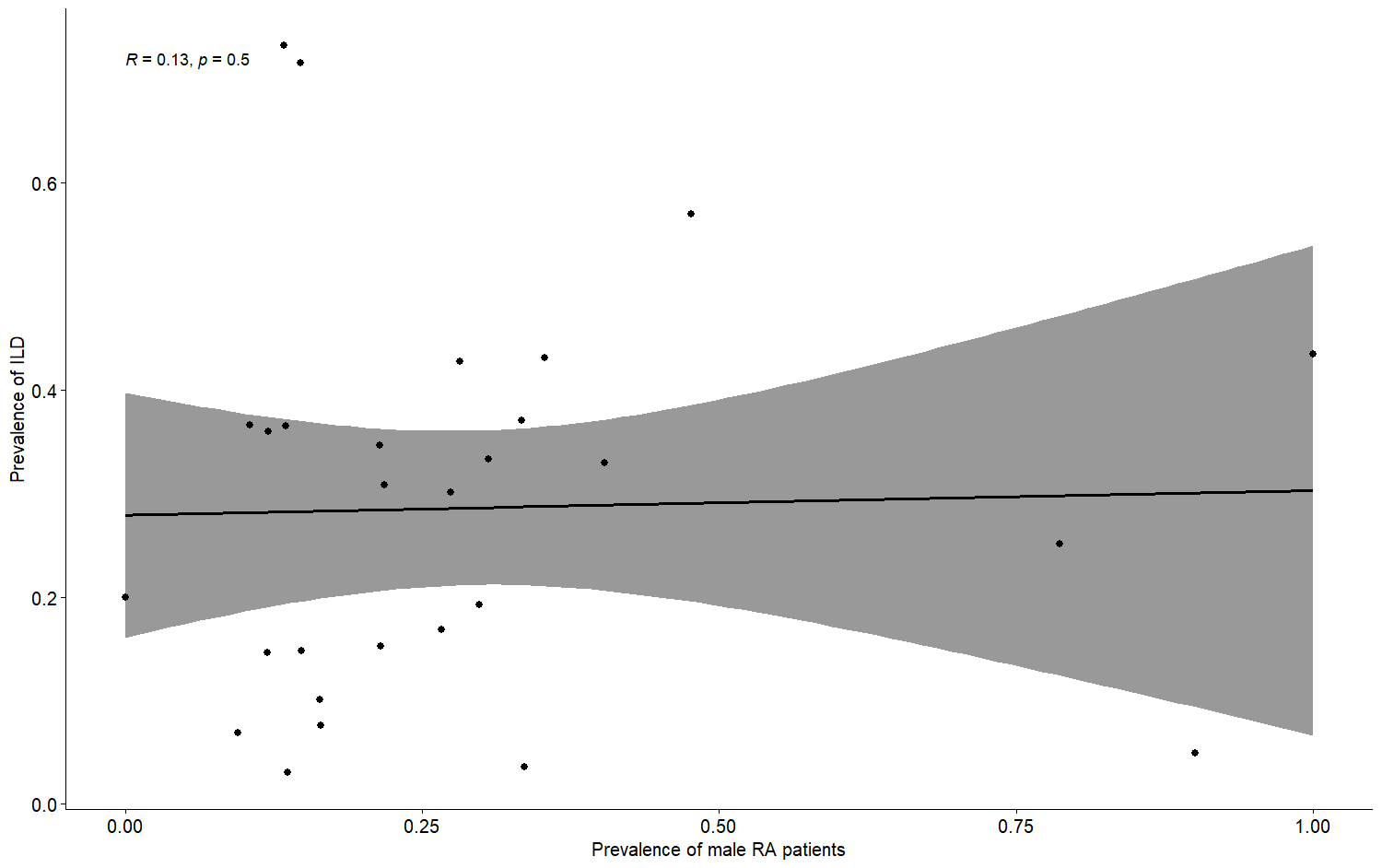


1. RA disease duration


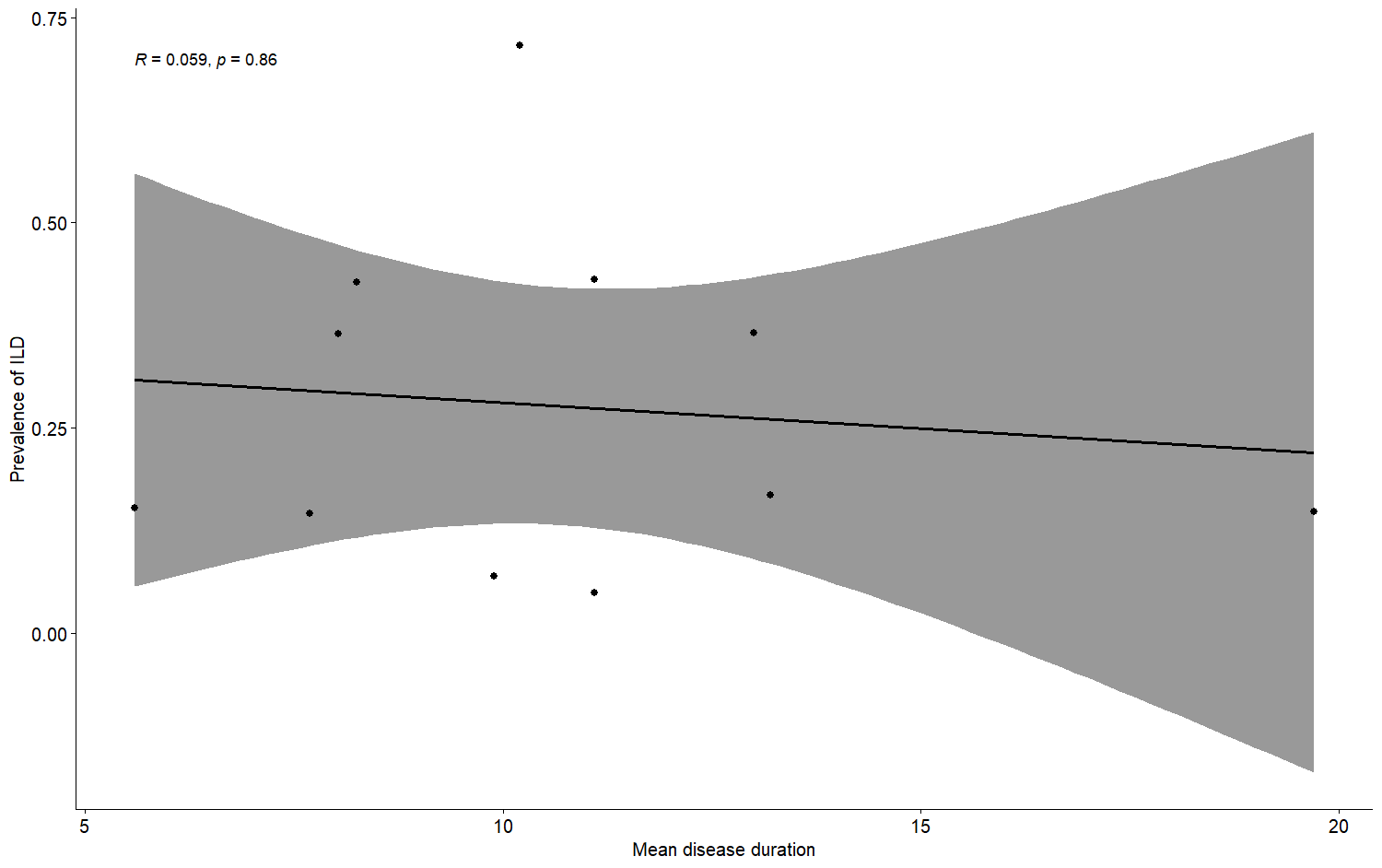


**Raw data:-**

| Author | Smoking prevalence | Prevalence of ILD |
| --- | --- | --- |
| Gabbay et al. | 0.555555556 | 0.3333 |
| Mori et al. | 0.17989418 | 0.1005 |
| Zou et al. | 0.172727273 | 0.4273 |
| Giles et al. | 0.710227273 | 0.3295 |
| Wang et al. | 0.068014706 | 0.1526 |
| Chen et al. USA | 0.38372093 | 0.5698 |
| Chen et al. China | 0.105263158 | 0.3083 |
| Okada et al. | 0.318637275 | 0.168 |
| Fadda et al. | 0.988636364 | 0.716 |
| Salaffi et al. | 0.238410596 | 0.1921 |
| Sherin et al. | 0.08 | 0.36 |
| Manfredi et al. | 0.372262774 | 0.4307 |
| England et al. | 0.795 | 0.0494 |
| Li L et al. | 0.260021668 | 0.3012 |
| Wickrematilake et al. | 0.0625 | 0.1458 |
| Paulin et al. | 0.53164557 | 0.0759 |
| Liang et al. Discovery cohort | 0.028571429 | 0.2 |
| Liang et al. Identification cohort | 0.051020408 | 0.3469 |
| Severo et al. | 0.335820896 | 0.3657 |
| Abdelwahab et al. | 0.666666667 | 0.733 |
| Sanaa et al. | 0.083333333 | 0.0303 |
| Razmjou et al. | 0.25 | 0.1481 |
| Ren et al. | 0.766233766 | 0.435 |

| Author | Mean age | Prevalence of ILD |
| --- | --- | --- |
| Gabbay et al. | 51.8 | 0.3333 |
| Zou et al. | 55.4 | 0.4273 |
| Giles et al. | 59 | 0.3295 |
| Wang et al. | 51.9 | 0.1526 |
| Okada et al. | 59.7 | 0.168 |
| Song et al. | 53.8 | 0.069 |
| Fadda et al. | 50.15 | 0.716 |
| Salaffi et al. | 53.4 | 0.1921 |
| Manfredi et al. | 67.9 | 0.4307 |
| England et al. | 63.5 | 0.0494 |
| Gautam et al. | 44.17 | 0.3704 |
| Wickrematilake et al. | 52.94 | 0.1458 |
| Paulin et al. | 46.49 | 0.0759 |
| Gutierrez et al. | 57.5 | 0.3649 |
| Severo et al. | 61 | 0.3657 |
| Abdelwahab et al. | 45.7 | 0.733 |
| Sanaa et al. | 44 | 0.0303 |
| Razmjou et al. | 63 | 0.1481 |
| Ren et al. | 66.6 | 0.435 |

| Author | Disease duration | Prevalence of ILD |
| --- | --- | --- |
| Zou et al. | 8.25 | 0.4273 |
| Wang et al. | 5.6 | 0.1526 |
| Okada et al. | 13.2 | 0.168 |
| Song et al. | 9.9 | 0.069 |
| Fadda et al. | 10.2 | 0.716 |
| Manfredi et al. | 11.1 | 0.4307 |
| England et al. | 11.1 | 0.0494 |
| Wickrematilake et al. | 7.69 | 0.1458 |
| Gutierrez et al. | 8.03 | 0.3649 |
| Severo et al. | 13 | 0.3657 |
| Razmjou et al. | 19.7 | 0.1481 |

| Author | Prevalence of males | Prevalence of ILD |
| --- | --- | --- |
| Gabbay et al. | 0.305555556 | 0.3333 |
| Koduri et al. | 0.33630137 | 0.0356 |
| Mori et al. | 0.164021164 | 0.1005 |
| Zou et al. | 0.281818182 | 0.4273 |
| Giles et al. | 0.403409091 | 0.3295 |
| Wang et al. | 0.215073529 | 0.1526 |
| Chen et al. USA | 0.476744186 | 0.5698 |
| Chen et al. China | 0.218045113 | 0.3083 |
| Okada et al. | 0.266533066 | 0.168 |
| Song et al. | 0.094827586 | 0.069 |
| Fadda et al. | 0.147727273 | 0.716 |
| Salaffi et al. | 0.298013245 | 0.1921 |
| Sherin et al. | 0.12 | 0.36 |
| Manfredi et al. | 0.35335689 | 0.4307 |
| England et al. | 0.901 | 0.0494 |
| Gautam et al. | 0.333333333 | 0.3704 |
| Li L et al. | 0.274106176 | 0.3012 |
| Wickrematilake et al. | 0.119791667 | 0.1458 |
| Paulin et al. | 0.164556962 | 0.0759 |
| Liang et al. Discovery cohort | 0 | 0.2 |
| Liang et al. Identification cohort | 0.214285714 | 0.3469 |
| Gutierrez et al. | 0.135135135 | 0.3649 |
| Severo et al. | 0.104477612 | 0.3657 |
| Abdelwahab et al. | 0.133333333 | 0.733 |
| Sanaa et al. | 0.136363636 | 0.0303 |
| Razmjou et al. | 0.148148148 | 0.1481 |
| Ren et al. | 1 | 0.435 |
| Yu et al. | 0.786610879 | 0.251 |

| Author | Methotrexate prevalence | Prevalence of ILD |
| --- | --- | --- |
| Gabbay et al. | 0.58 | 0.3333 |
| Giles et al. | 0.647727273 | 0.3295 |
| Wang et al. | 0.185661765 | 0.1526 |
| Chen et al. USA | 0.546511628 | 0.5698 |
| Chen et al. China | 0.962406015 | 0.3083 |
| Okada et al. | 0.595190381 | 0.168 |
| Salaffi et al. | 0.516556291 | 0.1921 |
| Sherin et al. | 0.92 | 0.36 |
| England et al. | 0.519 | 0.0494 |
| Li L et al. | 0.342361863 | 0.3012 |
| Wickrematilake et al. | 0.763020833 | 0.1458 |
| Paulin et al. | 0.602409639 | 0.0759 |
| Severo et al. | 0.925373134 | 0.3657 |
| Razmjou et al. | 0.462962963 | 0.1481 |
| Yu et al. | 0.552301255 | 0.251 |

| Author | Leflunomide prevalence | Prevalence of ILD |
| --- | --- | --- |
| Giles et al. | 0.107954545 | 0.3295 |
| Wang et al. | 0.152573529 | 0.1526 |
| Chen et al. USA | 0.186046512 | 0.5698 |
| Chen et al. China | 0.390977444 | 0.3083 |
| Sherin et al. | 0.48 | 0.36 |
| Li L et al. | 0.299024919 | 0.3012 |
| Paulin et al. | 0.084337349 | 0.0759 |
| Yu et al. | 0.19665272 | 0.251 |
